# Supplementary material for: Regulating the proximity effect of heterocycle-containing AIEgens
Source: Nat Commun. 2023 Jun 24;14:3772. doi: 10.1038/s41467-023-39479-1 (PMC10290688; doi:10.1038/s41467-023-39479-1)
Supplement: Supplementary file 1 — Supplementary Information [file 41467_2023_39479_MOESM1_ESM.pdf]

Supplementary Information

Regulating the proximity effect of heterocycle-containing AIEgens

Jianyu Zhang,[1,#] Yujie Tu,[1,#] Hanchen Shen,[1] Jacky W. Y. Lam,[1] Jianwei Sun,[1] Haoke Zhang,[2,3,*] and Ben Zhong Tang[1,4,5,*]

[1]Department of Chemistry, Hong Kong Branch of Chinese National Engineering Research Center for Tissue Restoration and Reconstruction, and Guangdong-Hong Kong-Macau Joint Laboratory of Optoelectronic and Magnetic Functional Materials, The Hong Kong University of Science and Technology, Clear Water Bay, Kowloon, Hong Kong 999077, China

[2]MOE Key Laboratory of Macromolecular Synthesis and Functionalization, Department of Polymer Science and Engineering, Zhejiang University, Hangzhou 310058, China

[3]Zhejiang-Israel Joint Laboratory of Self-Assembling Functional Materials, ZJU-Hangzhou Global Scientific and Technological Innovation Center, Zhejiang University, Hangzhou 311215, China

[4]School of Science and Engineering, Shenzhen Institute of Aggregate Science and Technology, The Chinese University of Hong Kong, Shenzhen, Guangdong 518172, China.

[5] AIE Institute, Guangzhou Development District, Huangpu, Guangzhou 510530, China

[#]These authors contributed equally: Jianyu Zhang, Yujie Tu.

[*]Corresponding emails: tangbenz@cuhk.edu.cn (Ben Zhong Tang); zhanghaoke@zju.edu.cn (Haoke Zhang)

Table of Contents

Supplementary Methods **3**
Synthesis **3**
Supplementary Figure 1. General synthetic routes to SQ, MeOSQ, and DPASQ 3
Supplementary Figure 2. Synthetic routes to MeMQ 4
Nuclear Magnetic Resonance (NMR) Spectra **6**
Supplementary Figure 3. (a) ^{1}H and (b) ^{13}C NMR spectra of MQ in DMSO-d_6. 6
Supplementary Figure 4. (a) ^{1}H and (b) ^{13}C NMR spectra of SQ in DMSO-d_6 6
Supplementary Figure 5. (a) ^{1}H and (b) ^{13}C NMR spectra of MeOSQ in DMSO-d_6. 7
Supplementary Figure 6. (a) ^{1}H and (b) ^{13}C NMR spectra of DPASQ in DMSO-d_6 7
Supplementary Figure 7. (a) ^{1}H and (b) ^{13}C NMR spectra of MeMQ in DMSO-d_6 8
High-Resolution Mass Spectrometry **8**
Supplementary Figure 8. High-resolution mass spectrum of MQ with negative electrospray ionization. 8
Supplementary Figure 9. High-resolution mass spectrum of SQ with negative electrospray ionization 9
Supplementary Figure 10. High-resolution mass spectrum of MeOSQ with negative electrospray ionization. 9
Supplementary Figure 11. High-resolution mass spectrum of DPASQ with negative electrospray ionization 10
Supplementary Figure 12. High-resolution mass spectrum of MeMQ with negative electrospray ionization 10
Other Characterizations **11**
Supplementary Figure 13. The normalized absorption spectra of MQ, SQ, MeOSQ, and DPASQ 11
Supplementary Figure 14. The size distribution of MQ, SQ, MeOSQ, and DPASQ in the THF/water mixtures with different water fractions 12
Supplementary Figure 15. Photoluminescence spectra, fluorescent photographs and absolute quantum yield, and powder X-ray diffraction patterns of MQ, SQ, MeOSQ, and DPASQ in the solid state. 13
Supplementary Table 1. Summary of crystallographic data and structural refinements 13
Supplementary Figure 16. Crystal packing diagram of MQ 14
Supplementary Figure 17. Crystal packing diagram of SQ 14
Supplementary Figure 18. Crystal packing diagram of MeOSQ 14
Supplementary Figure 19. Crystal packing diagram of DPASQ. 14
Supplementary Figure 20. Photophysical properties of MeOSQ and DPASQ in MeOH/water mixtures 15
Supplementary Figure 21. Time-resolved photoluminescence decay curves of MQ, SQ, MeOSQ, and DPASQ...... 16
Supplementary Figure 22. Photoluminescence spectra of MQ, SQ, MeOSQ, and DPASQ in different solvents with increasing polarity 17
Supplementary Table 2. Reichardt's parameters and normalized Reichardt's parameters of different solvents, and relative PL intensity of MQ, SQ, MeOSQ, and DPASQ in different solvents 17
Supplementary Figure 23. The photophysical properties of methyl-substituted MQ (MeMQ) 18
Supplementary Figure 24. Comparison of ^{13}C NMR spectra of MQ in DMSO-d_6 and THF-d_8 19
Supplementary Figure 25. The schematic Jablonski diagrams based on different energy levels of (n,π*) and (π,π*) states. 19
Supplementary Table 3. The calculated energy levels and hole-electron analysis of the lowest-lying (n,π*) and (π,π*) states of MQ in different solvents 20
Supplementary Table 4. The calculated energy levels and hole-electron analysis of the lowest-lying (n,π*) and (π,π*) states of SQ in different solvents. 21
Supplementary Table 5. The calculated energy levels and hole-electron analysis of the lowest-lying (n,π*) and (π,π*) states of MeOSQ in different solvents 22
Supplementary Table 6. The calculated energy levels and hole-electron analysis of the lowest-lying (n,π*) and (π,π*) states of DPASQ in different solvents 23
Supplementary Table 7. The calculated energy levels and hole-electron analysis of the lowest-lying (n,π*) and (π,π*) states of MeMQ in different solvents 24
Supplementary Figure 26. Absorption spectra of MQ, MeOSQ, and DPASQ solutions in DCM upon the addition of trifluoroacetic acid 25
Supplementary Figure 27. Frontier molecular orbitals of deprotonated and protonated compounds based on their optimized ground-state geometries 26
Supplementary Figure 28. Frontier molecular orbitals and oscillator strength of the deprotonated and protonated compounds based on their optimized excited-state geometries. 27
Cartesian Coordinates of the Optimized Molecular Geometry **28**
Supplementary References **117**

Supplementary Methods

Synthesis

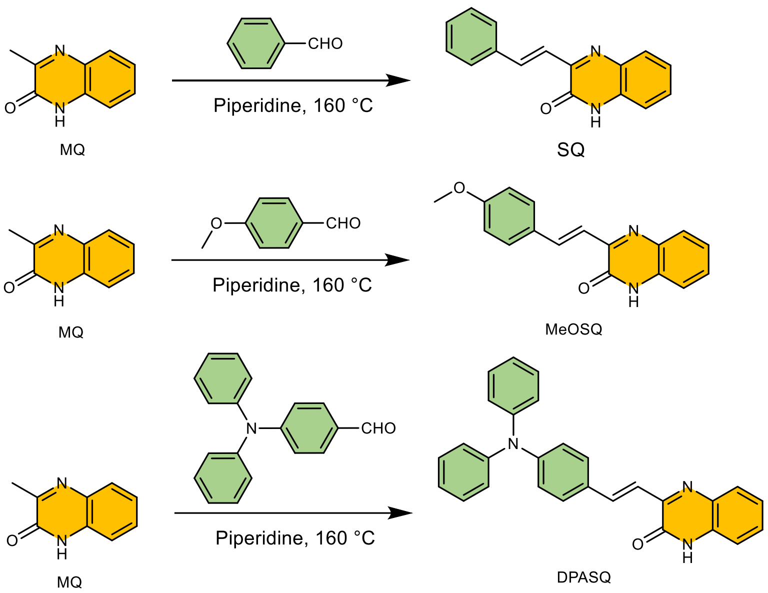

Supplementary Figure 1. General synthetic routes to SQ, MeOSQ, and DPASQ.

The purchased 3-methylquinoxalin-2(1H)-one (MQ) which appeared to be black powders was purified with column chromatography followed by recrystallization to obtain pale-yellow crystals. The synthetic route was reported in a previous work.[1]

(E)-3-styrylquinoxalin-2(1H)-one (SQ): 1.60 g (10 mmol) of MQ and 1.06 g (10 mmol) benzaldehyde were mixed in a 100 mL round-bottom flask and heated up to 160 °C, and 5 mL of piperidine was added into the flask. After reacting for 10 min, 50 mL of ethanol was added into the mixture. The suspension was filtrated and washed with ethanol three times (15 mL × 3). The washed products were purified by column chromatography with ethyl acetate as eluent. The pure product of SQ was 1.36 g with a yield of 55%. The yellow crystalline samples were obtained from recrystallization by slowly evaporating the solvent of acetone for single-crystal X-ray measurement.

(E)-3-(4-methoxystyryl)quinoxalin-2(1H)-one (MeOSQ): 1.60 g (10 mmol) of MQ and 1.36 g (10 mmol) 4-methoxybenzaldehyde were mixed in a 100 mL round-bottom flask and heated up to 160 °C, and 5 mL of piperidine was added into the flask. After reacting for 10 min, 50 mL of ethanol was added into the mixture. Then the suspension was filtrated and washed with ethanol three times (15 mL × 3). The washed products were purified by column chromatography with ethyl acetate as eluent. The pure product of MeOSQ was 1.17 g with a yield of 42%. The yellow crystalline samples were obtained from recrystallization by slowly evaporating the solvent of chloroform and dichloromethane for single-crystal X-ray measurement.

(E)-3-(4-(diphenylamino)styryl)quinoxalin-2(1H)-one (DPASQ): 1.60 g (10 mmol) of MQ and 2.73 g (10 mmol) 4-(diphenylamino)benzaldehyde were mixed in a 100 mL round-bottom flask and heated up to 160 °C, and 5 mL of piperidine was added into the flask. After reacting for 10 min, 50 mL of ethanol was added into the mixture. Then the suspension was filtrated and washed with ethanol three times (15 mL × 3). The washed products were purified by column chromatography with ethyl acetate as eluent. The pure product of DPASQ was 1.57 g with a yield of 38%. The orange crystalline samples were

obtained from recrystallization by slowly evaporating the solvent of dichloromethane for single-crystal X-ray measurement.

1-methylquinoxalin-2(1H)-one (MeMQ)

1.00 g (6.25 mmol) of MQ and 0.47 mL (1.2 equiv., 1.06 g, 7.5 mmol) iodomethane were added in a 50 mL round-bottom flask with 1.04 g K_2CO_3 (1.2 equiv., 7.5 mmol) and 15 mL DMF. The mixture was stirred under room temperature overnight. Then, brine was added, and the mixture was extracted three times with ethyl acetate. The combined organic layers were washed with a saturated solution of NH_4Cl and dried over anhydrous Na_2SO_4, filtered and evaporated in *vacuo*. The residue was purified by column chromatography on silica gel to afford pale yellow product of MeMQ (1.03 g) with a yield of 95%.

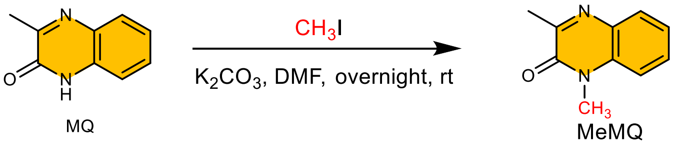

Supplementary Figure 2. Synthetic routes to MeMQ.

Below, NMR characterization data recorded in DMSO-d_6 and HRMS data are listed (this work).

3-methylquinoxalin-2(1H)-one (MQ)

^{1}H NMR (400 MHz, DMSO-d_6), δ (ppm): 12.30 (s, 1H), 7.69-7.67 (d, J = 7.7 Hz, 1H), 7.48-7.44 (t, J = 7.5 Hz, 1H), 7.28-7.24 (m, 2H), 2.39 (s, 3H). ^{13}C NMR (400 MHz, DMSO-d_6), δ (ppm):159.7, 155.4, 132.4, 132.1, 129.8, 128.3, 123.5, 115.7, 21.0. HRMS: m/z: calculated for $[C_9H_9N_2O]^+$: 161.0709 $[M+H]^+$; found: 161.0719.

(E)-3-styrylquinoxalin-2(1H)-one (SQ)

^{1}H NMR (400 MHz, DMSO-d_6), δ (ppm): 12.52 (s, 1H), 8.10-8.05 (d, J = 8.1 Hz, 1H), 7.80-7.79 (d, J = 7.8 Hz, 1H), 7.75-7.73 (d, J = 7.7 Hz, 2H), 7.65-7.61 (d, J = 7.6 Hz, 1H), 7.53-7.39 (m, 4H), 7.34-7.30 (t, J = 7.3 Hz, 2H). ^{13}C NMR (400 MHz, DMSO-d_6), δ (ppm):155.3, 153.5, 137.6, 136.5, 132.8, 132.2, 130.3, 129.9, 129.5, 128.8, 128.1, 124.0, 122.5, 115.7. HRMS: m/z: calculated for $[C_{16}H_{13}N_2O]^+$: 249.1022 $[M+H]^+$; found: 249.1023.

(E)-3-(4-methoxystyryl)quinoxalin-2(1H)-one (MeOSQ)

^{1}H NMR (400 MHz, DMSO-d_6), δ (ppm): 12.45 (s, 1H), 8.02-7.98 (d, J = 8.0 Hz, 1H), 7.75-7.73 (d, J = 7.7 Hz, 1H), 7.68-7.66 (d, J = 7.7 Hz, 1H), 7.49-7.43 (m, 2H), 7.30-7.26 (t, J = 7.3 Hz, 1H), 6.99-6.97 (d, J = 7.0 Hz, 1H), 3.79 (s, 3H). ^{13}C NMR (400 MHz, DMSO-d_6), δ (ppm): 160.9, 155.3, 153.7, 137.4, 132.9, 132.0, 129.9, 129.8, 129.1, 128.6, 123.9, 119.9, 115.7, 115.0, 55.8. HRMS: m/z: calculated for $[C_{17}H_{15}N_2O_2]^+$: 279.1128 $[M+H]^+$; found: 279.1137.

(E)-3-(4-(diphenylamino)styryl)quinoxalin-2(1H)-one (DPASQ)

^{1}H NMR (400 MHz, DMSO-d_6), δ (ppm): 12.45 (s, 1H), 8.01-7.97 (d, J = 8.0 Hz, 1H), 7.74-7.72 (d, J = 7.7 Hz, 1H), 7.62-7.60 (d, J = 7.6 Hz, 2H), 7.49-7.43 (m, 2H), 7.37-7.33 (t, J = 7.3 Hz, 4H), 7.29-7.26 (m, 2H), 7.14-7.08 (m, 6H), 6.93-6.91 (d, J = 6.9 Hz, 2H),. ^{13}C NMR (400 MHz, DMSO-d_6), δ (ppm): 155.3, 153.6, 148.9, 147.0, 137.1, 132.9, 132.0, 130.2, 129.9, 129.8, 129.5, 128.6, 125.5, 124.5, 123.9, 121.9, 120.0, 115.7. HRMS: m/z: calculated for $[C_{28}H_{21}N_3O]^+$: 415.1685 $[M+H]^+$; found: 415.1687.

1-methylquinoxalin-2(1H)-one (MeMQ)

^{1}H NMR (400 MHz, DMSO-d_6), δ (ppm): 7.75-7.73 (d, J = 8.0 Hz, 1H), 7.61-7.57 (t, J = 8.0 Hz, 1H), 7.54-7.51 (d, J = 8.0 Hz, 1H), 7.38-7.34 (m, 1H), 3.62 (s, 1H), 2.44 (s, 1H). ^{13}C NMR (400 MHz, DMSO-d_6), 158.1, 154.8, 133.6, 132.4, 130.1, 129.1, 123.7, 115.1, 29.4, 21.7. HRMS: m/z: calculated for $[C_{16}H_{13}N_2O]^+$: 175.0866 $[M+H]^+$; found: 175.0874.

Nuclear Magnetic Resonance (NMR) Spectra

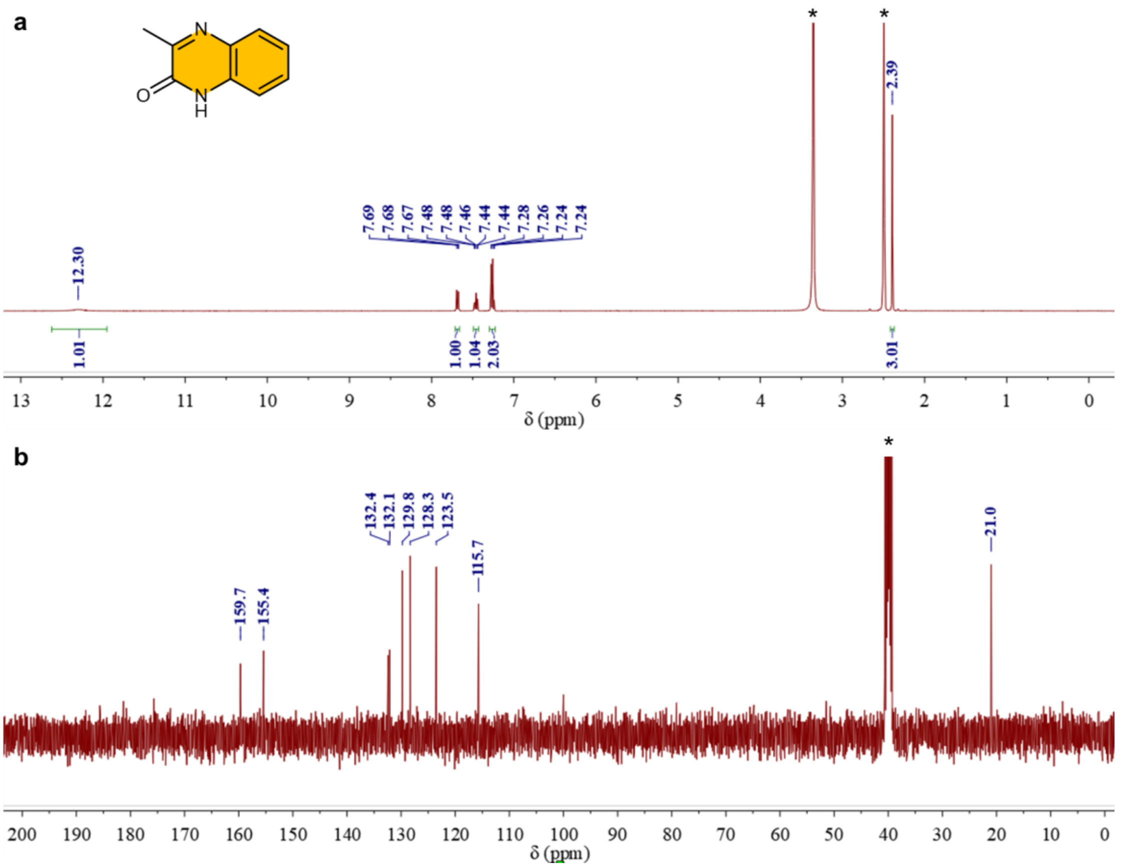

Supplementary Figure 3. (a) ^{1}H and (b) ^{13}C NMR spectra of MQ in DMSO-d_6.

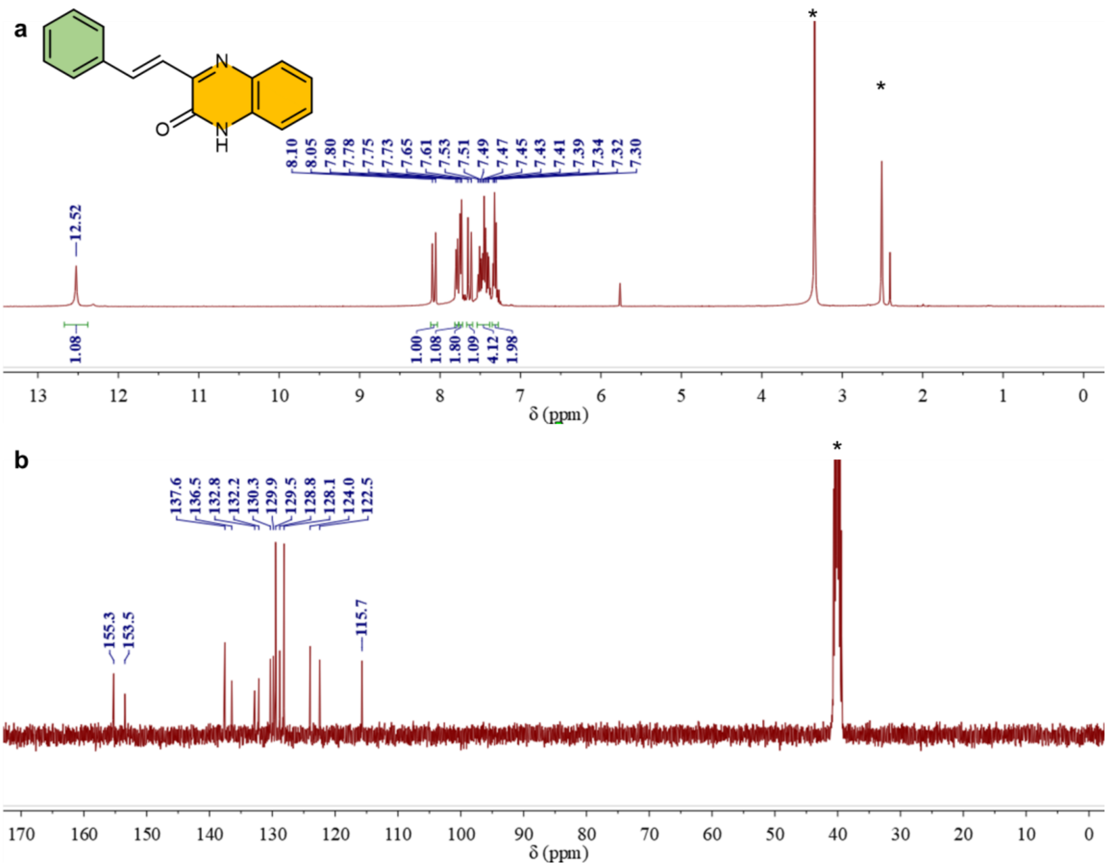

Supplementary Figure 4. (a) ^{1}H and (b) ^{13}C NMR spectra of SQ in DMSO-d_6.

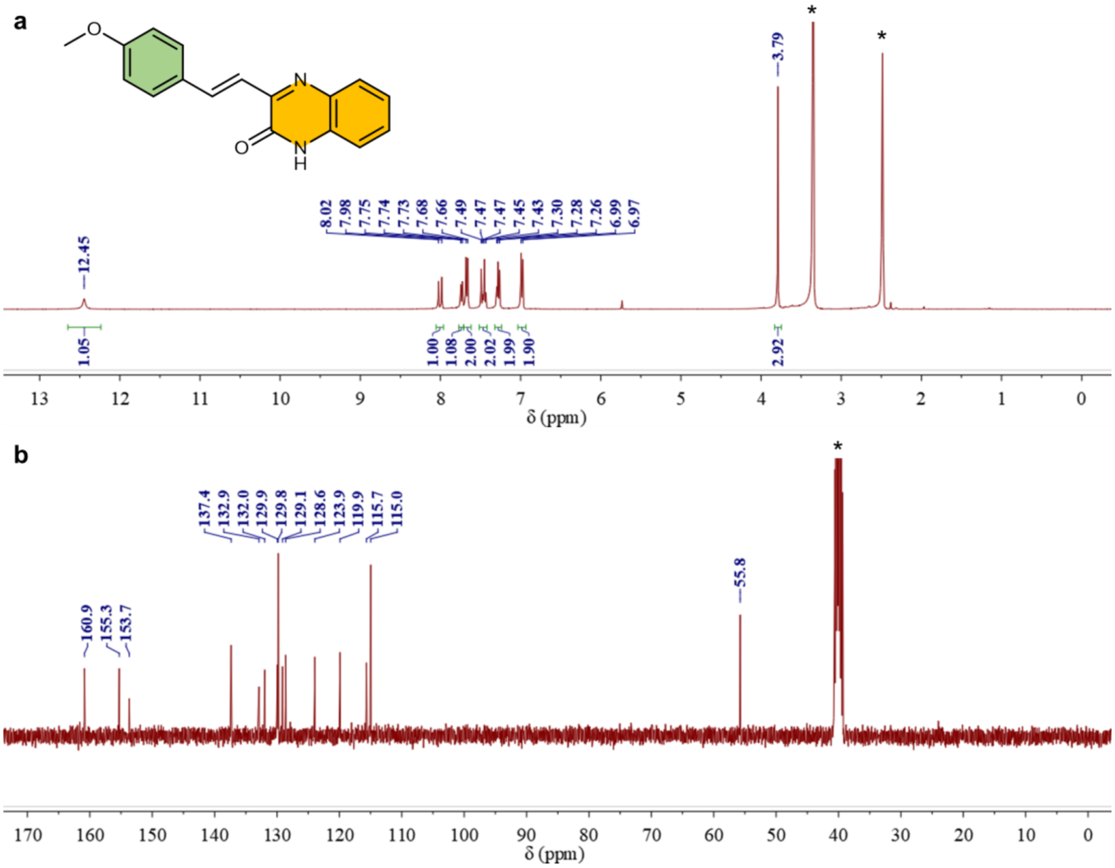

Supplementary Figure 5. (a) ^{1}H and (b) ^{13}C NMR spectra of MeOSQ in DMSO-d_6.

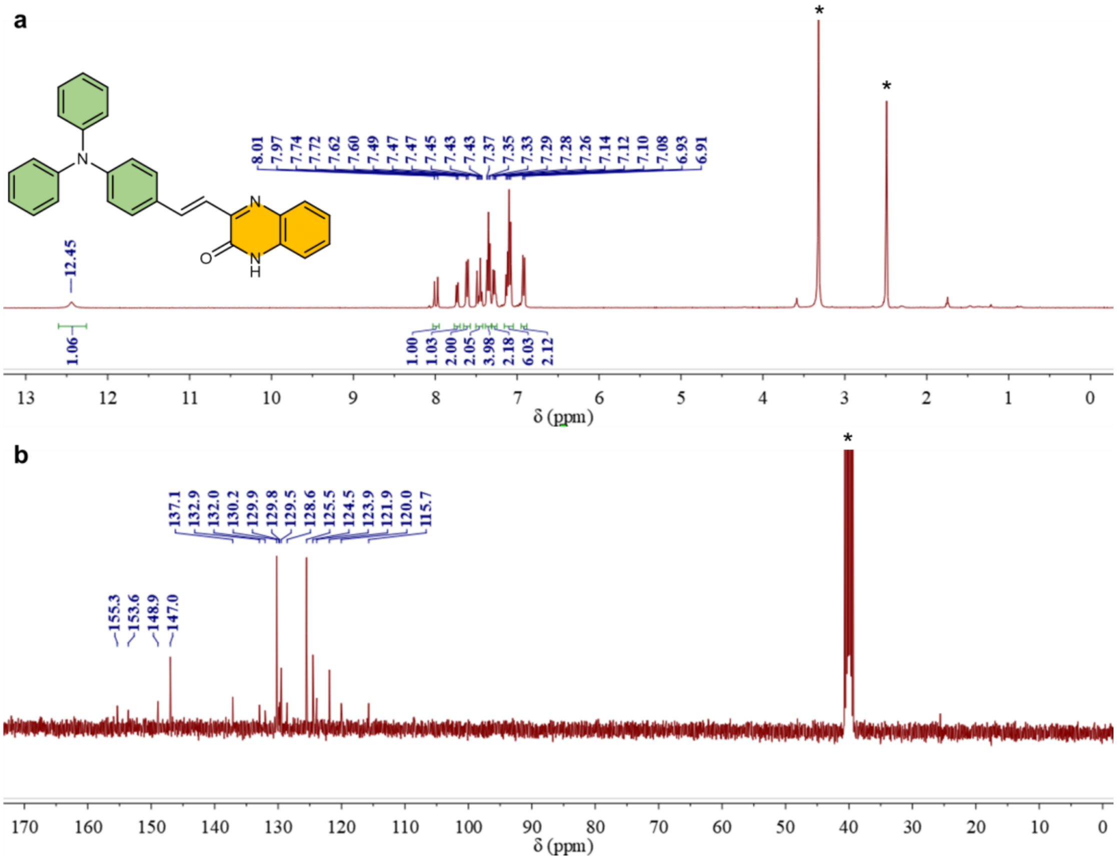

Supplementary Figure 6. (a) ^{1}H and (b) ^{13}C NMR spectra of DPASQ in DMSO-d_6.

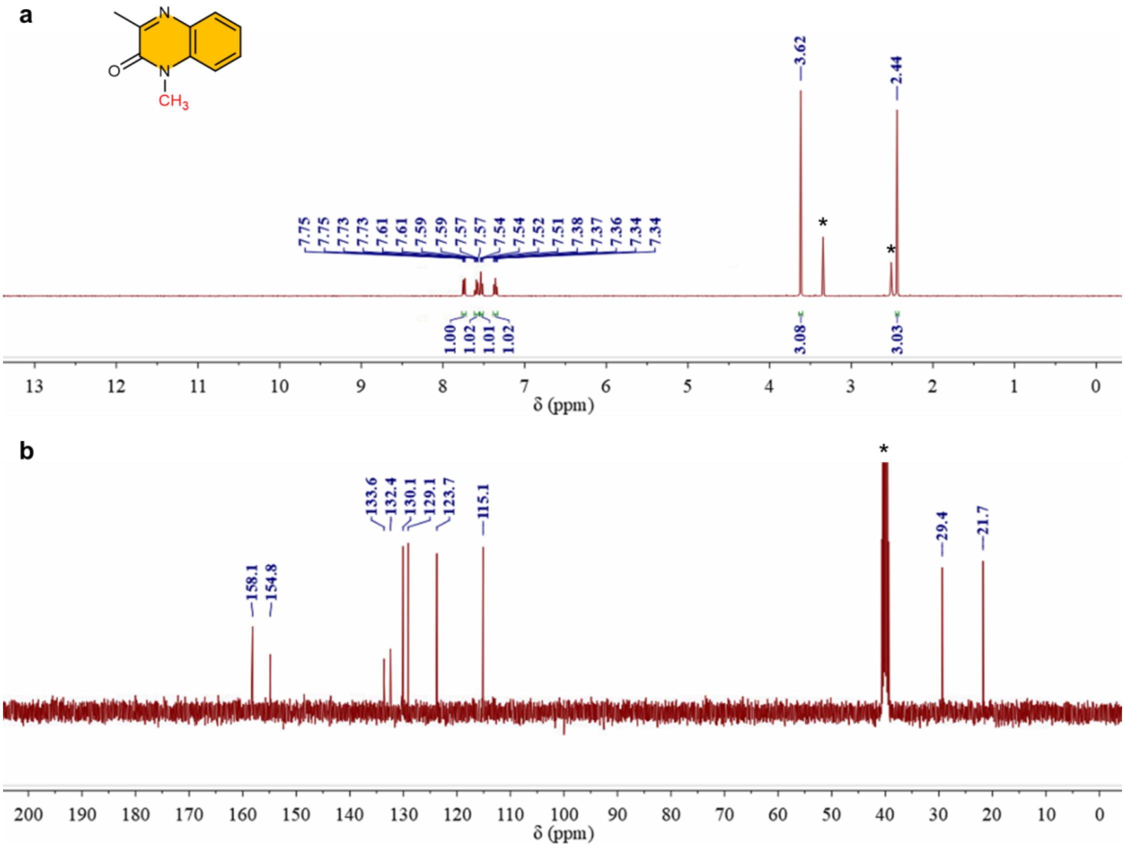

Supplementary Figure 7. (a) ^{1}H and (b) ^{13}C NMR spectra of MeMQ in DMSO-d_6.

High-Resolution Mass Spectrometry

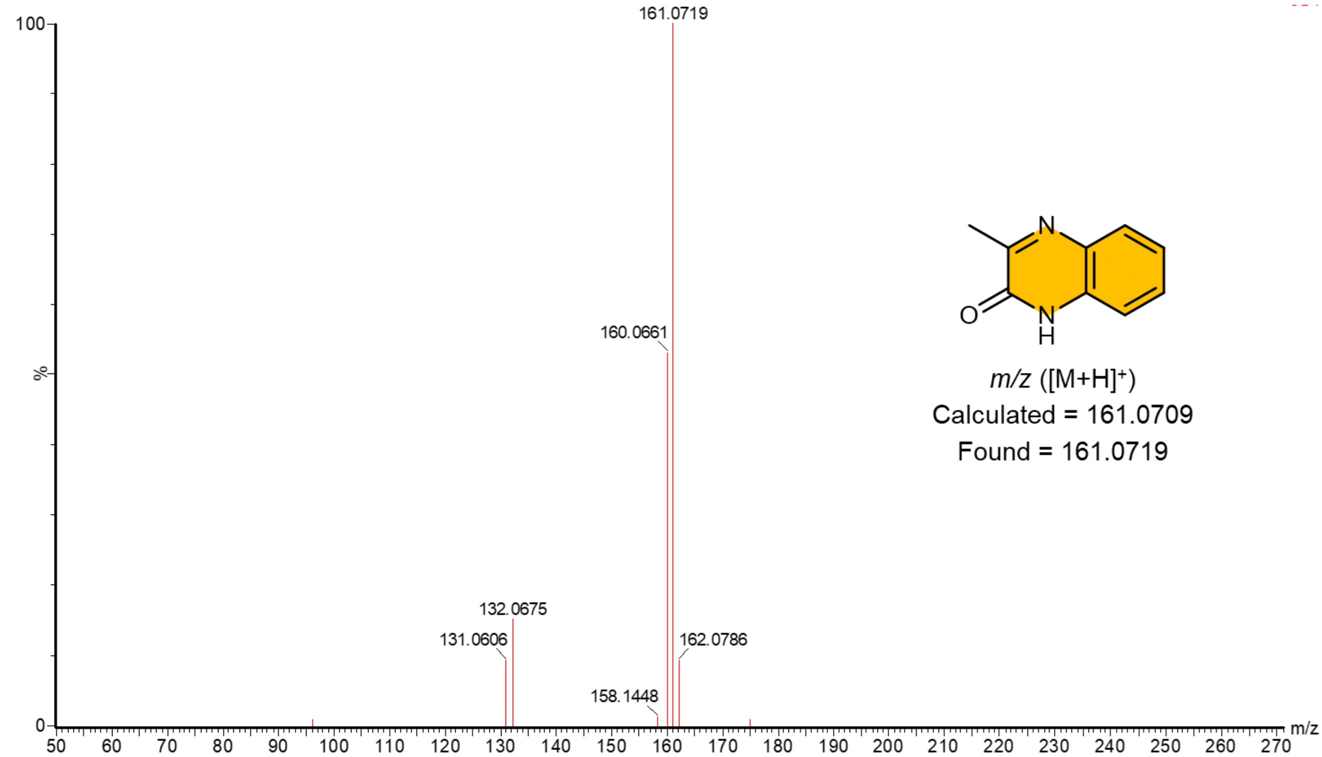

Supplementary Figure 8. High-resolution mass spectrum of MQ with negative electrospray ionization.

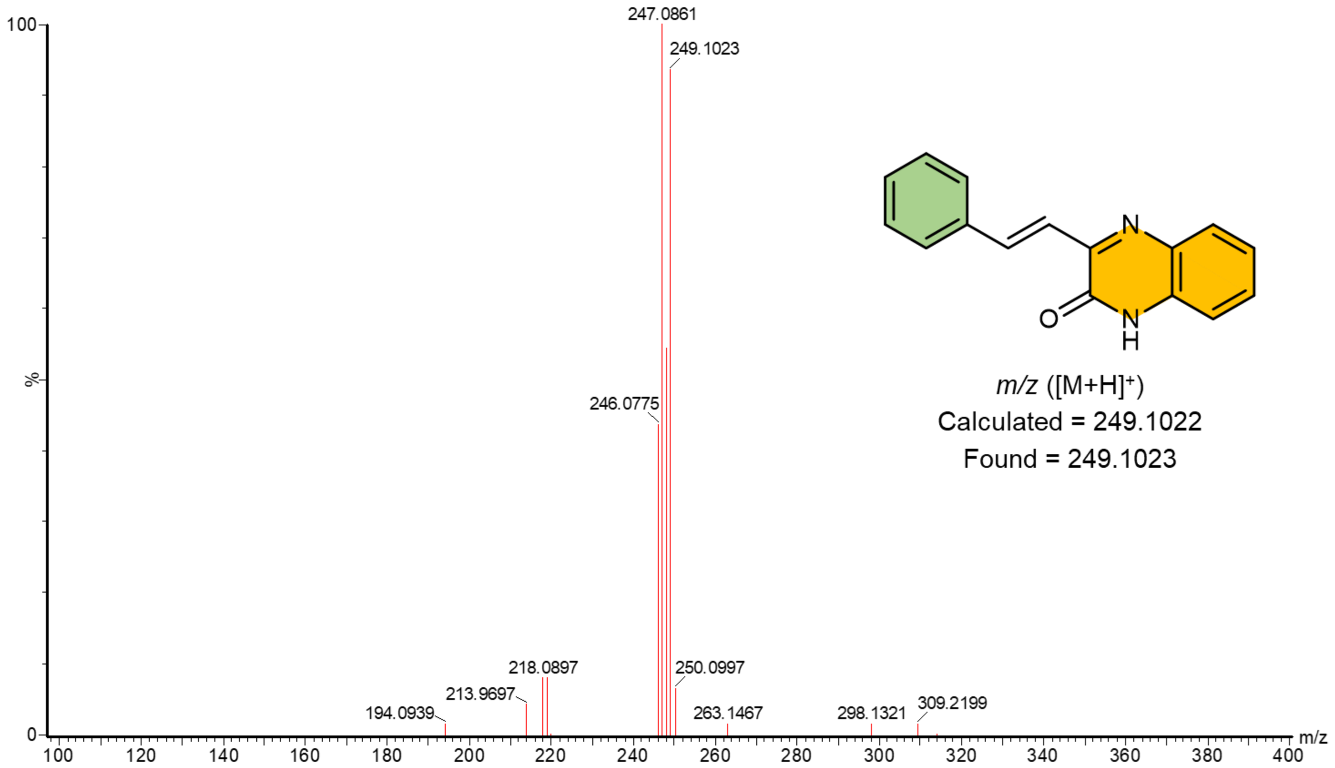

Supplementary Figure 9. High-resolution mass spectrum of SQ with negative electrospray ionization.

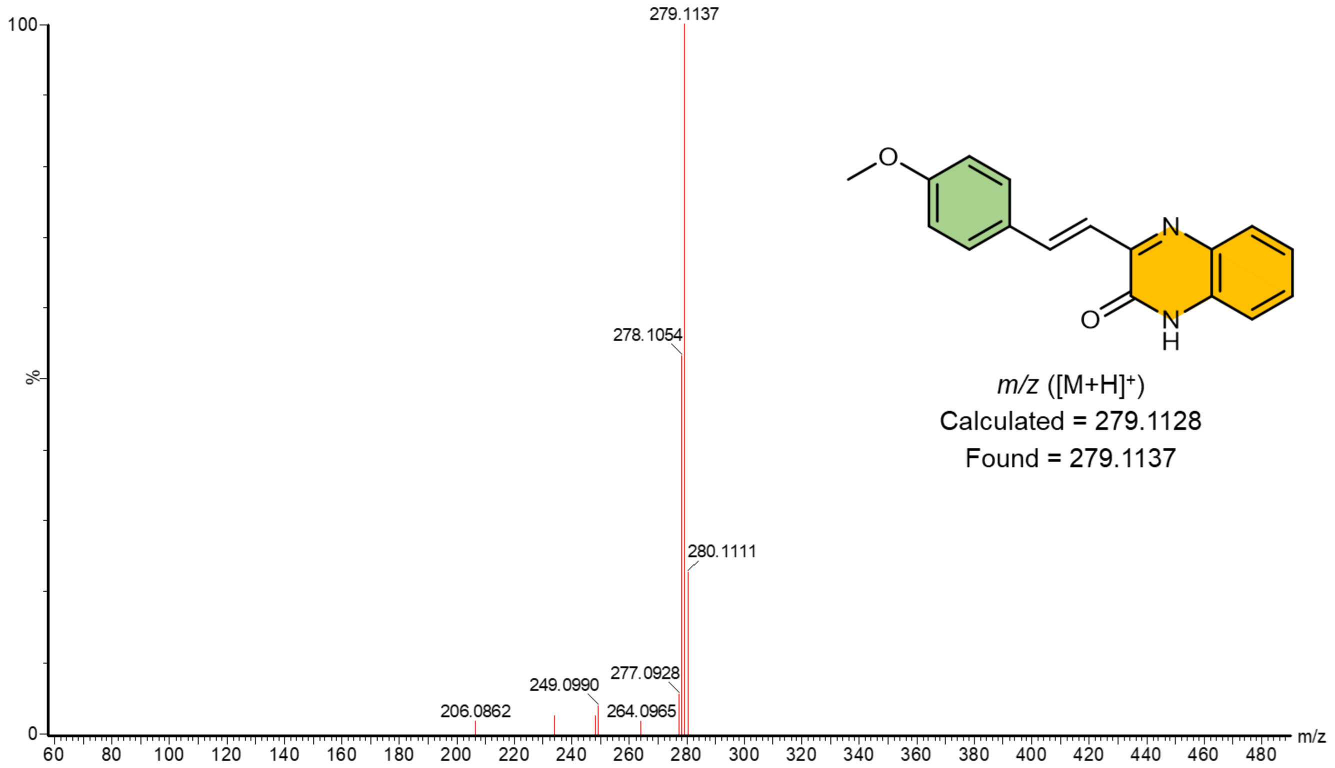

Supplementary Figure 10. High-resolution mass spectrum of MeOSQ with negative electrospray ionization.

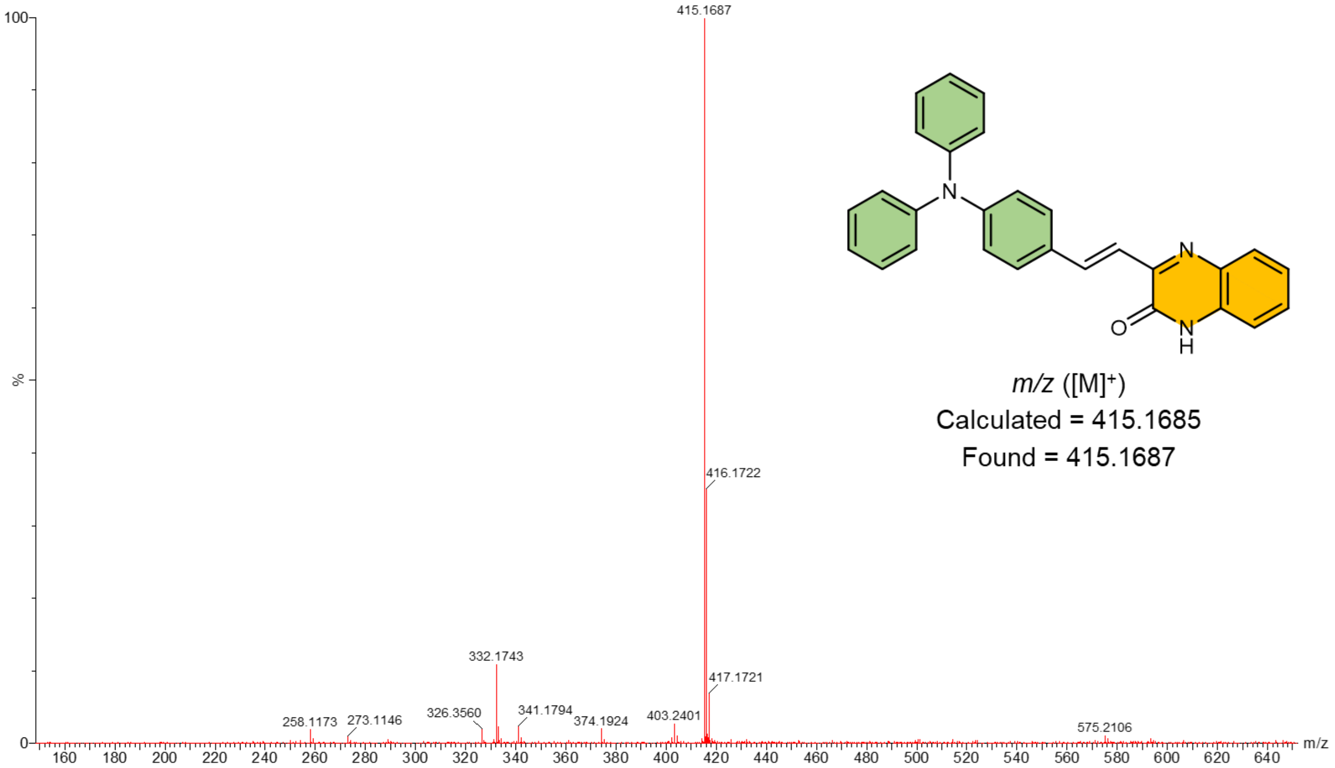

Supplementary Figure 11. High-resolution mass spectrum of DPASQ with negative electrospray ionization.

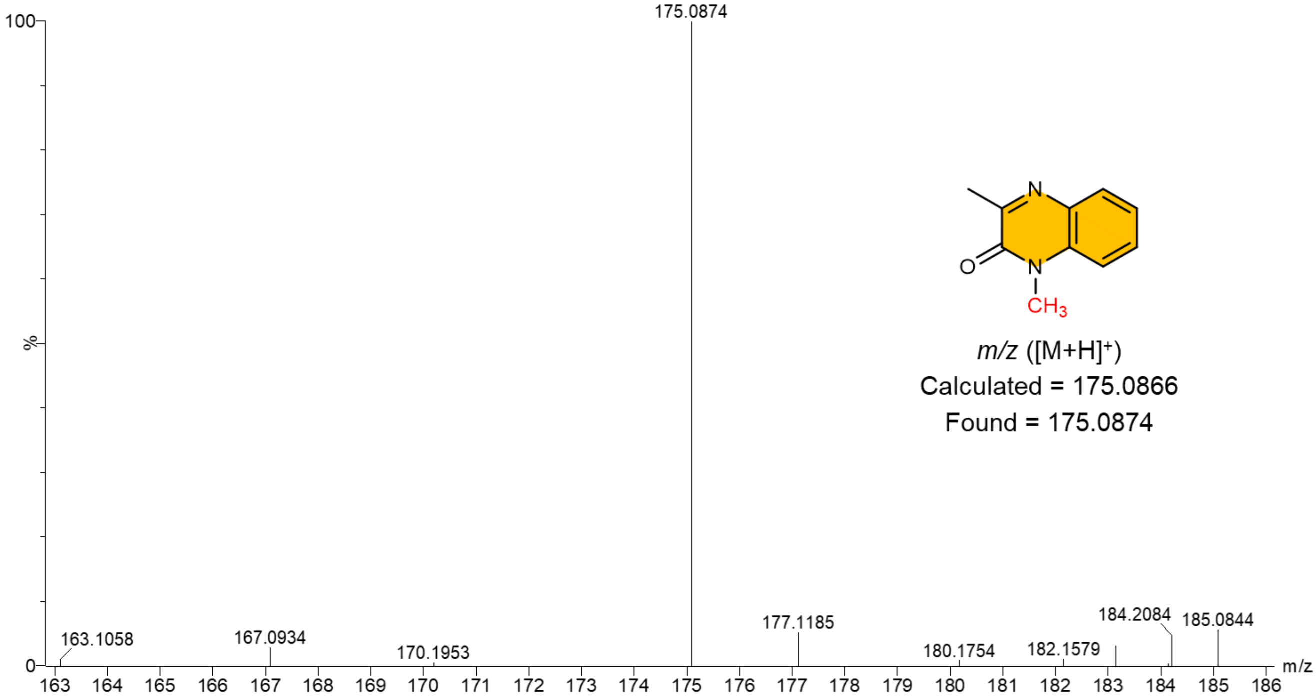

Supplementary Figure 12. High-resolution mass spectrum of MeMQ with negative electrospray ionization.

Other Characterizations

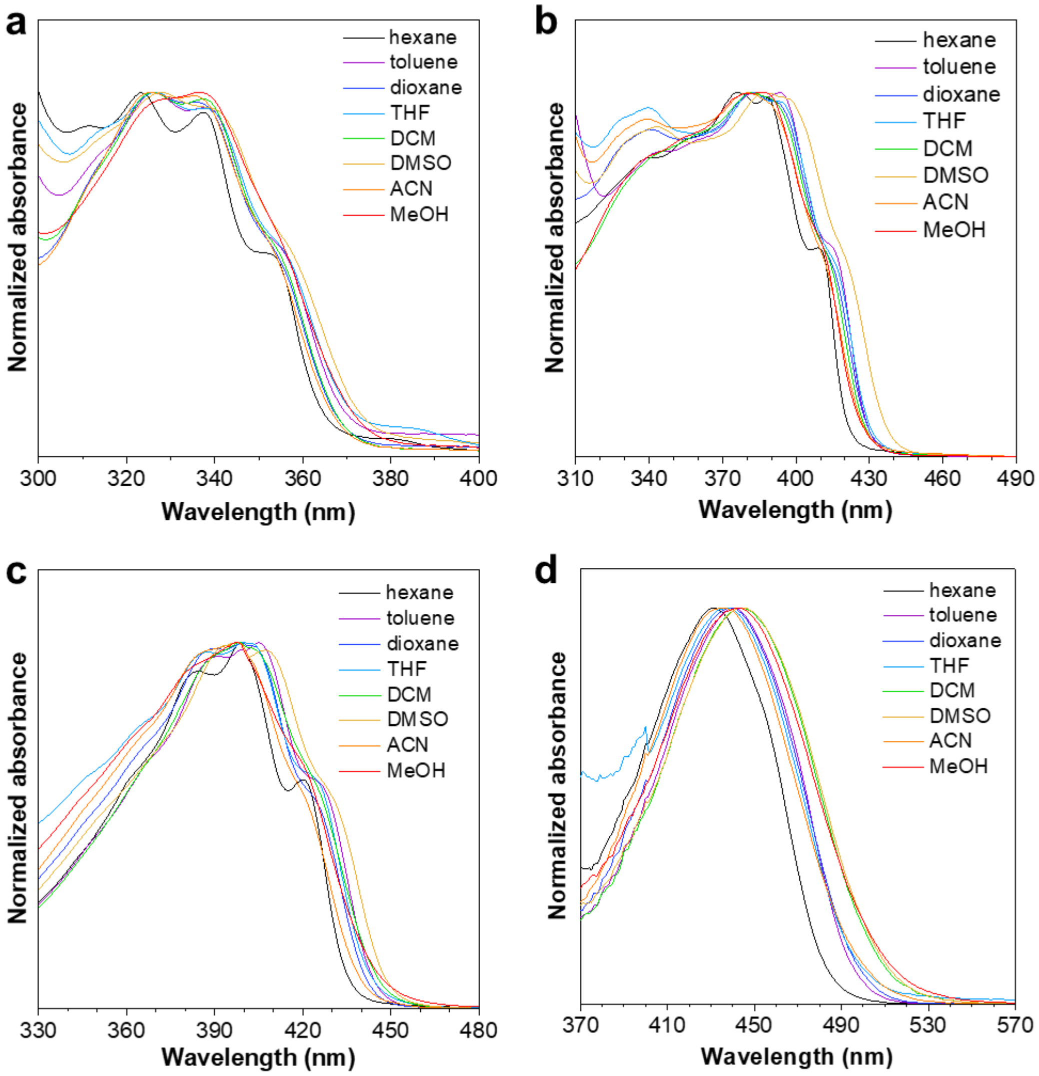

Supplementary Figure 13. The normalized absorption spectra of (a) MQ, (b) SQ, (c) MeOSQ, and (d) DPASQ in different solvents. Concentration = 10^{-5} M.

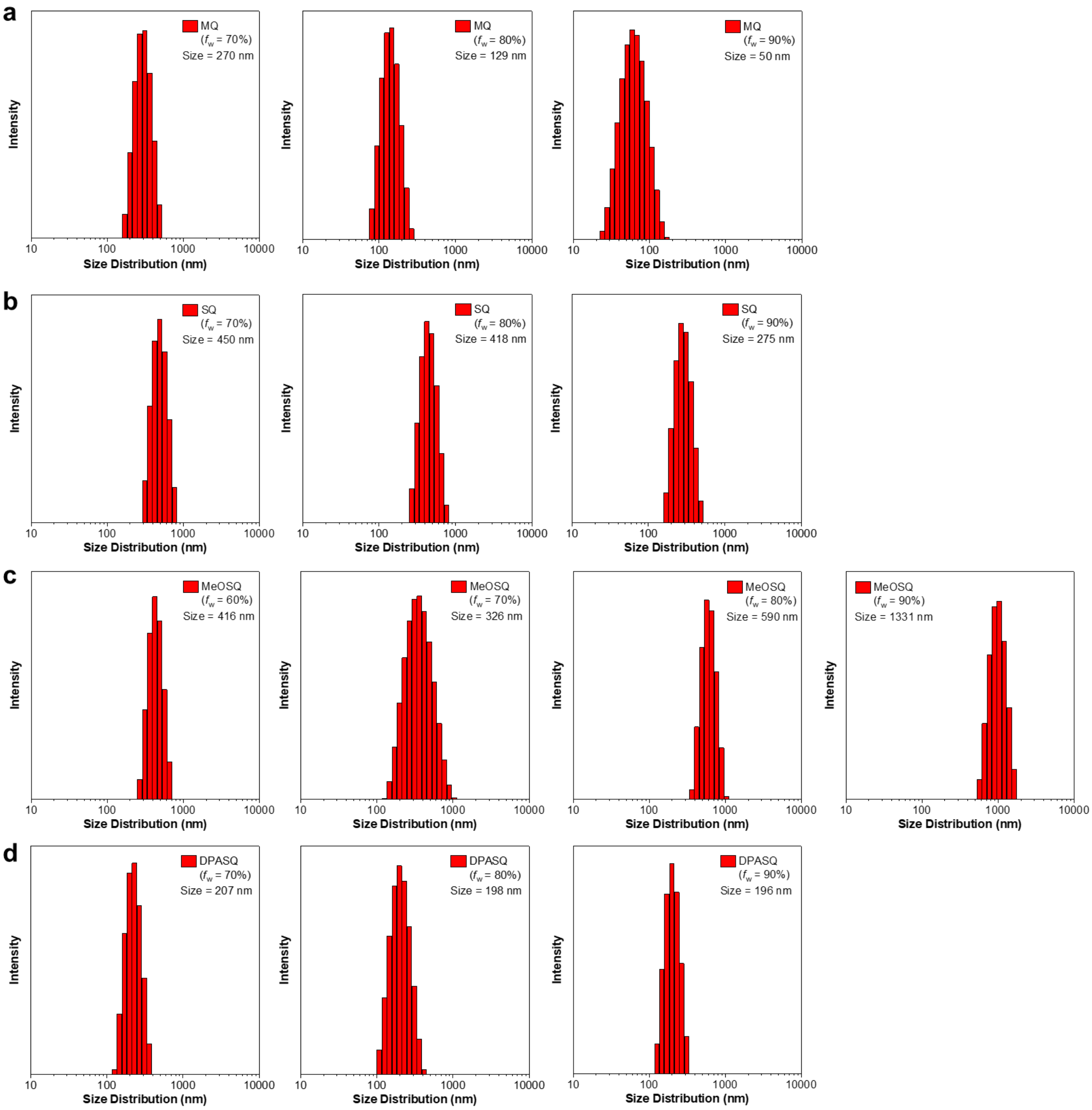

Supplementary Figure 14. The size distribution of (a) MQ, (b) SQ, (c) MeOSQ, and (d) DPASQ in the THF/water mixtures with different water fractions. Concentration = 10^{-5} M.

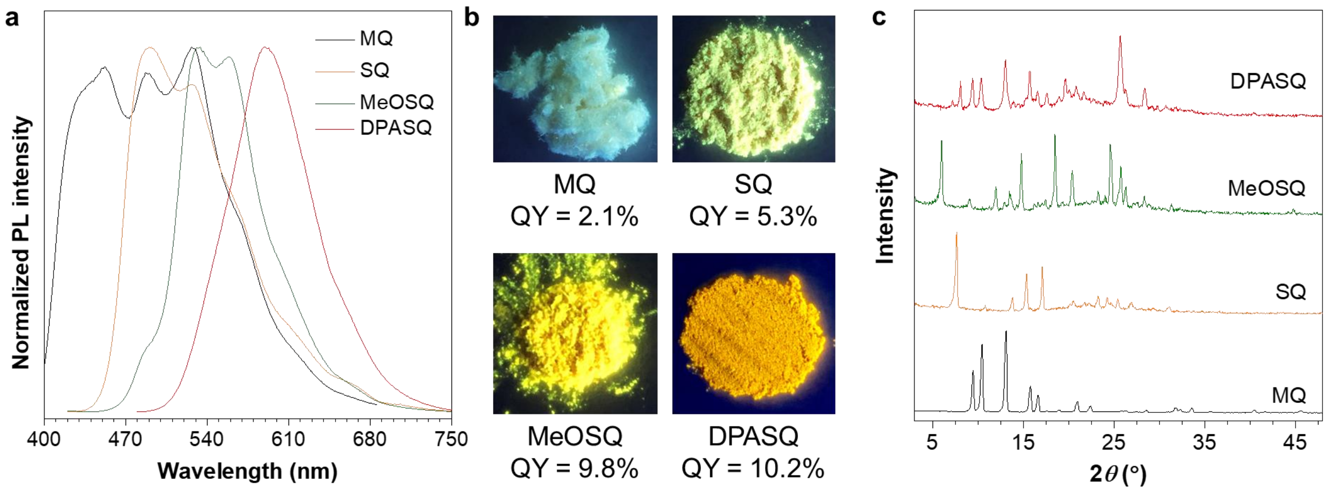

Supplementary Figure 15. (a) Photoluminescence spectra, (b) fluorescent photographs and absolute quantum yield (QY), and (c) powder X-ray diffraction patterns of MQ, SQ, MeOSQ, and DPASQ in the crystalline state.

Supplementary Table 1. Summary of crystallographic data and structural refinements

Compound	MQ	SQ	MeOSQ	DPASQ
CCDC number	2213287	2213288	2213289	2213290
Empirical formula	$C_9H_8N_2O$	$C_{16}H_{12}N_2O$	$C_{17}H_{14}N_2O_2$	$C_{28}H_{21}N_3O$
Formula weight	160.17	248.28	278.30	415.48
Temperature/K	100.00(10)	100(2)	100.01(10)	100.01(10)
Crystal system	monoclinic	orthorhombic	monoclinic	triclinic
Space group	$P2_1/c$	Pca21	$P2_1/c$	P-1
a/Å	3.94733(16)	22.7905(3)	14.8112(3)	12.7398(4)
b/Å	11.2158(4)	4.65100(10)	4.51270(7)	13.3190(6)
c/Å	16.9368(7)	23.1355(3)	20.8859(4)	14.0034(6)
α/°	90	90	90	114.045(4)
β/°	91.308(4)	90	106.620(2)	100.515(3)
γ/°	90	90	90	95.189(3)
Volume/Å^3	749.64(5)	2452.33(7)	1337.66(4)	2097.24(16)
Z	4	8	4	4
Density/ g·cm^{-3}	1.419	1.345	1.382	1.316
F(000)	336.0	1040.0	584.0	872.0
μ/mm^{-1}	0.781	0.684	0.745	0.640
Goodness-of-fit on F^2	1.031	1.040	1.054	1.020
R_1 [I>=2σ (I)][a]	0.0416	0.0418	0.0363	0.0442
wR_2 [I>=2σ (I)][b]	0.1072	0.1150	0.0985	0.1095
R_1 [all data][a]	0.0538	0.0437	0.0411	0.0636
wR_2 [all data][b]	0.1177	0.1175	0.1032	0.1189

[b]$R_1 = F_o - F_c/F_o$

[c]$wR_2 = \{w[(F_o)^2 - (F_c)^2]^2/w[(F_o)^2]^2\}^{1/2}$

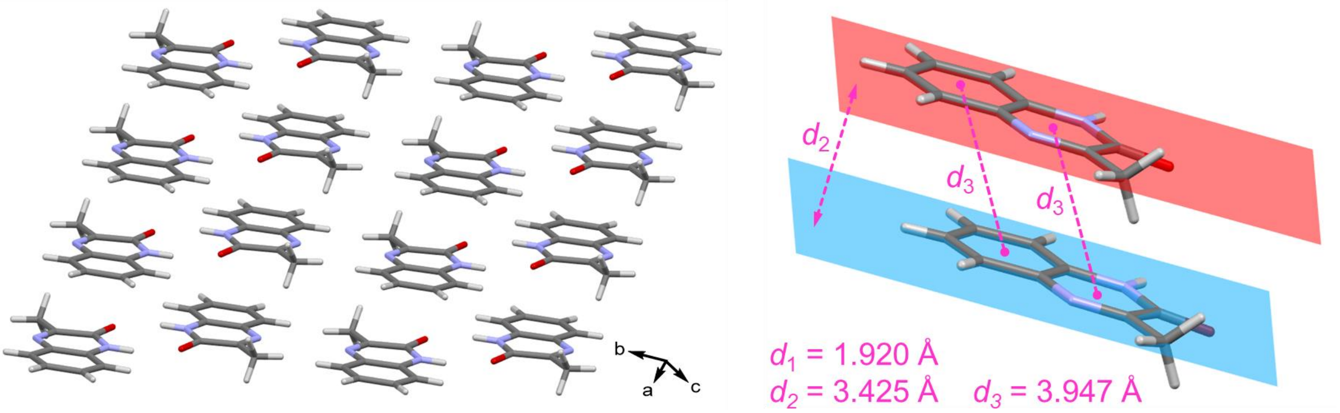

Supplementary Figure 16. Crystal packing diagram of MQ.

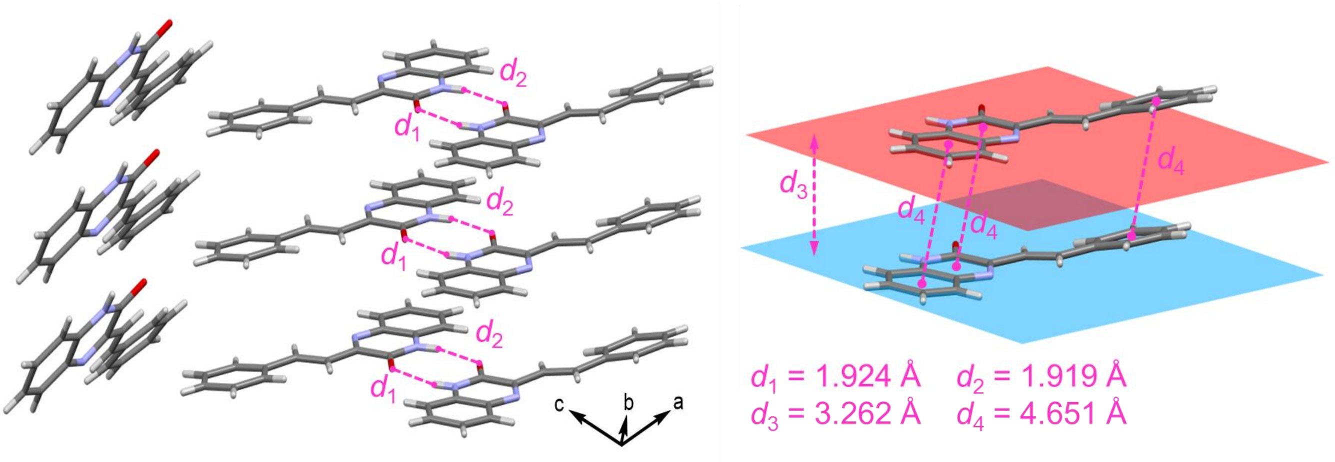

Supplementary Figure 17. Crystal packing diagram of SQ.

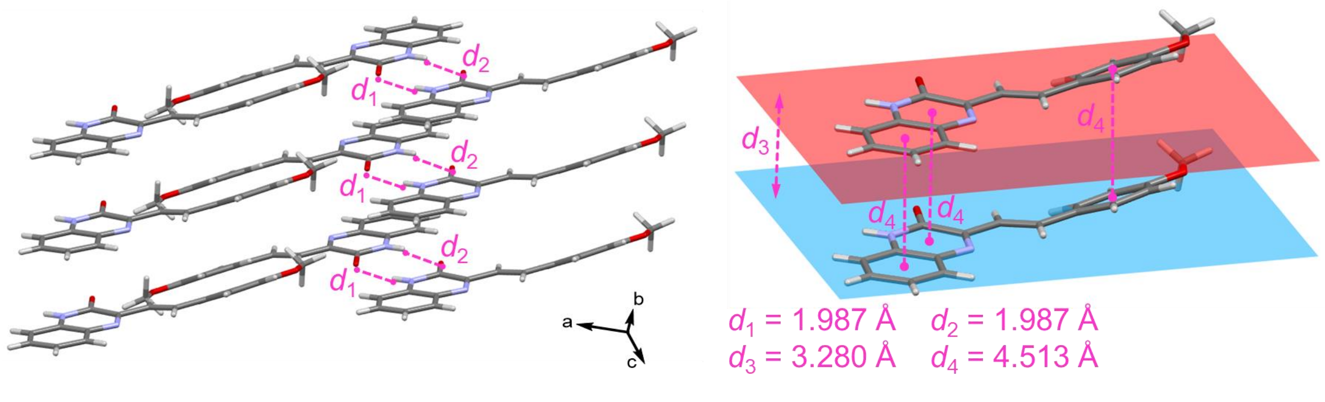

Supplementary Figure 18. Crystal packing diagram of MeOSQ.

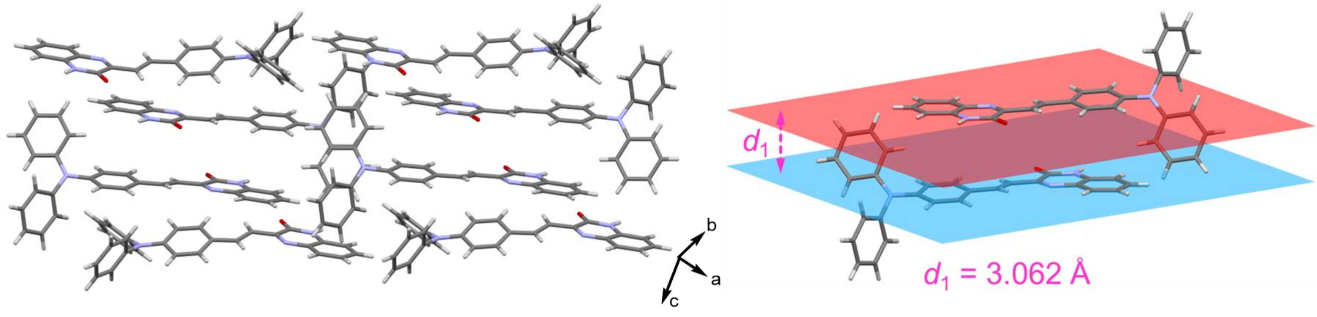

Supplementary Figure 19. Crystal packing diagram of DPASQ.

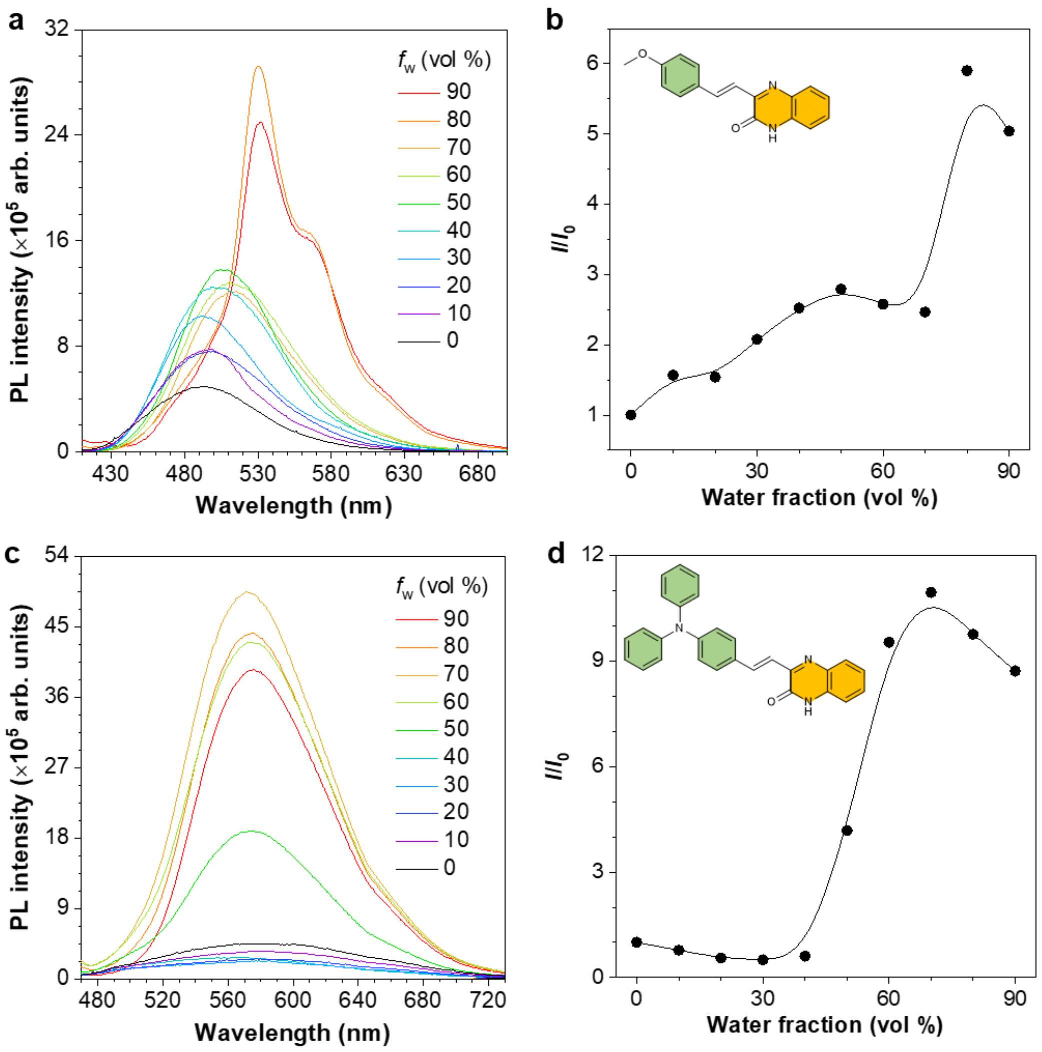

Supplementary Figure 20. Photophysical properties of MeOSQ and DPASQ in MeOH/water mixtures. **a** Photoluminescence (PL) spectra of MeOSQ in MeOH/water mixtures with different water fractions (f_w). Concentration (c) = 10^{-5} M, excitation wavelength (λ_{ex}) = 390 nm. **b** Plots of relative PL intensity (I/I_0) versus f_w. I_0 = PL intensity at f_w = 0%. **c** PL spectra of DPASQ in MeOH/water mixtures with different f_w. $c = 10^{-5}$ M, λ_{ex} = 430 nm. **d** Plots of I/I_0 versus f_w.

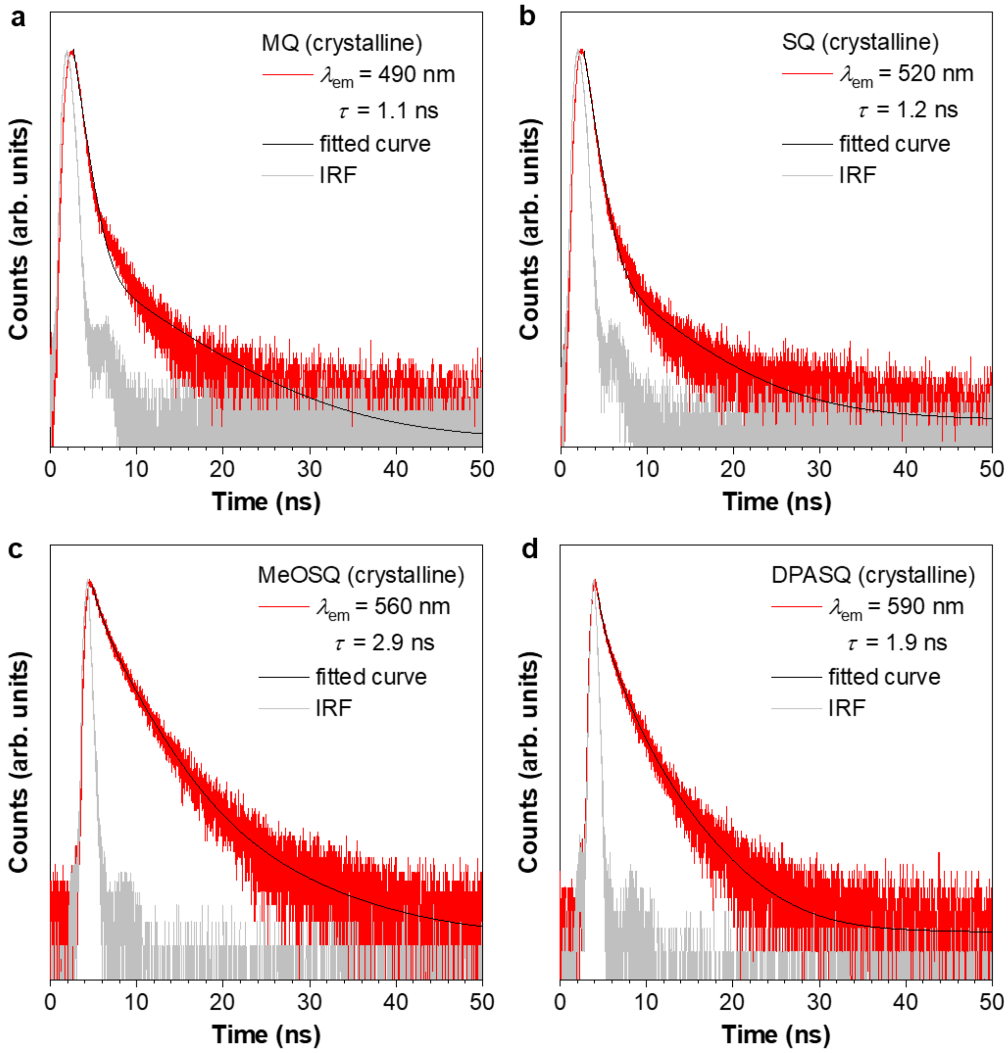

Supplementary Figure 21 Time-resolved photoluminescence decay curves of (a) MQ, (b) SQ, (c) MeOSQ, and (d) DPASQ taken at their corresponding maximum emission wavelength (λ_{em}) in the crystalline state.

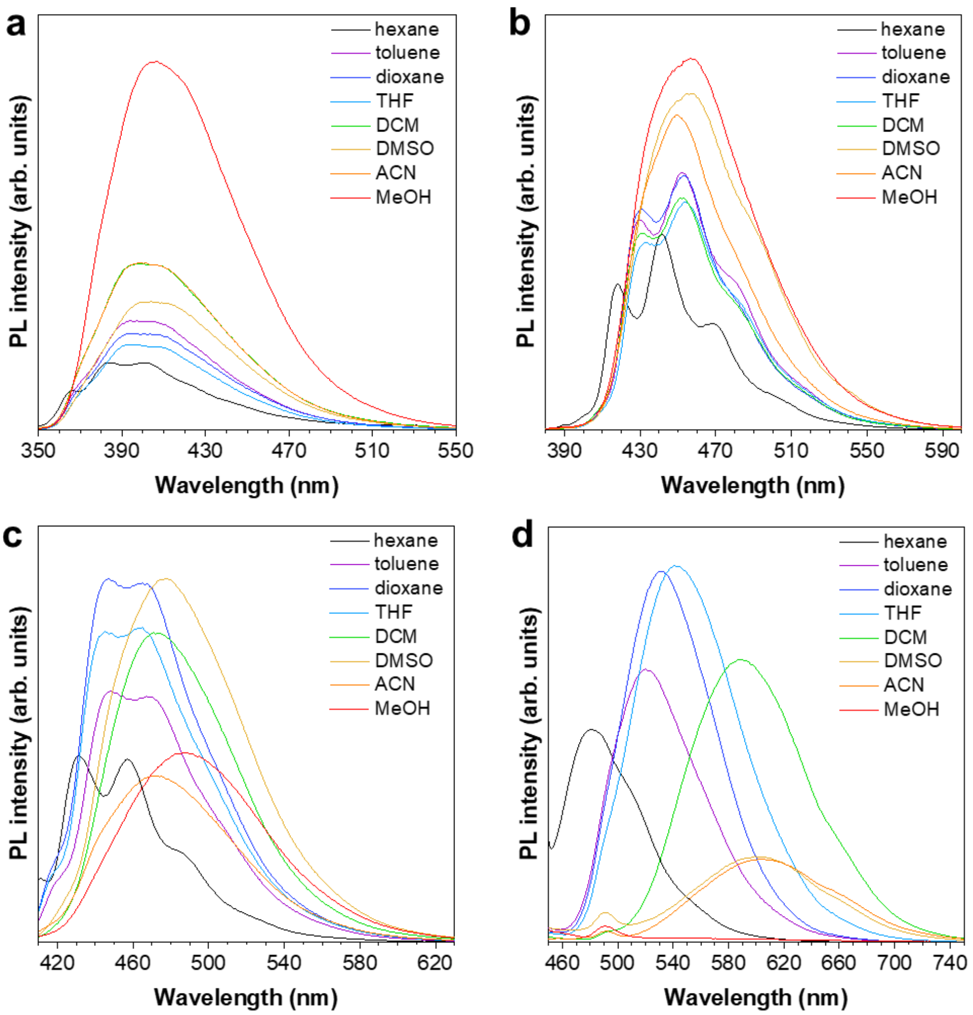

Supplementary Figure 22. Photoluminescence (PL) spectra of (a) MQ, (b) SQ, (c) MeOSQ, and (d) DPASQ in different solvents with increased polarity (*n*-hexane (hexane) < toluene < dioxane < tetrahydrofuran (THF) < dichloromethane (DCM) < dimethyl sulfoxide (DMSO) < acetonitrile (ACN) < methanol (MeOH)). Concentration = 10^{-5} M.

Supplementary Table 2. Reichardt's parameters and normalized Reichardt's parameters of different solvents, and relative PL intensity (I/I_{min}) of MQ, SQ, MeOSQ, and DPASQ in different solvents.[2]

		n-Hexane	Toluene	Dioxane	THF	DCM	DMSO	ACN	MeOH
Reichardt's parameter (E_T)		31.0	33.9	36.0	37.4	40.7	45.1	45.6	55.4
Normalized Reichardt's parameter (E_T^N)		0.009	0.099	0.164	0.207	0.309	0.444	0.460	0.762
Relative PL intensity	MQ	1.000	1.618	1.434	1.268	2.460	1.902	2.475	5.461
	SQ	1.000	1.312	1.297	1.163	1.182	1.715	1.606	1.892
	MeOSQ	1.123	2.191	2.191	1.895	1.867	2.191	1.000	1.139
	DPASQ	13.221	16.971	23.045	23.407	17.578	5.324	5.165	1.000

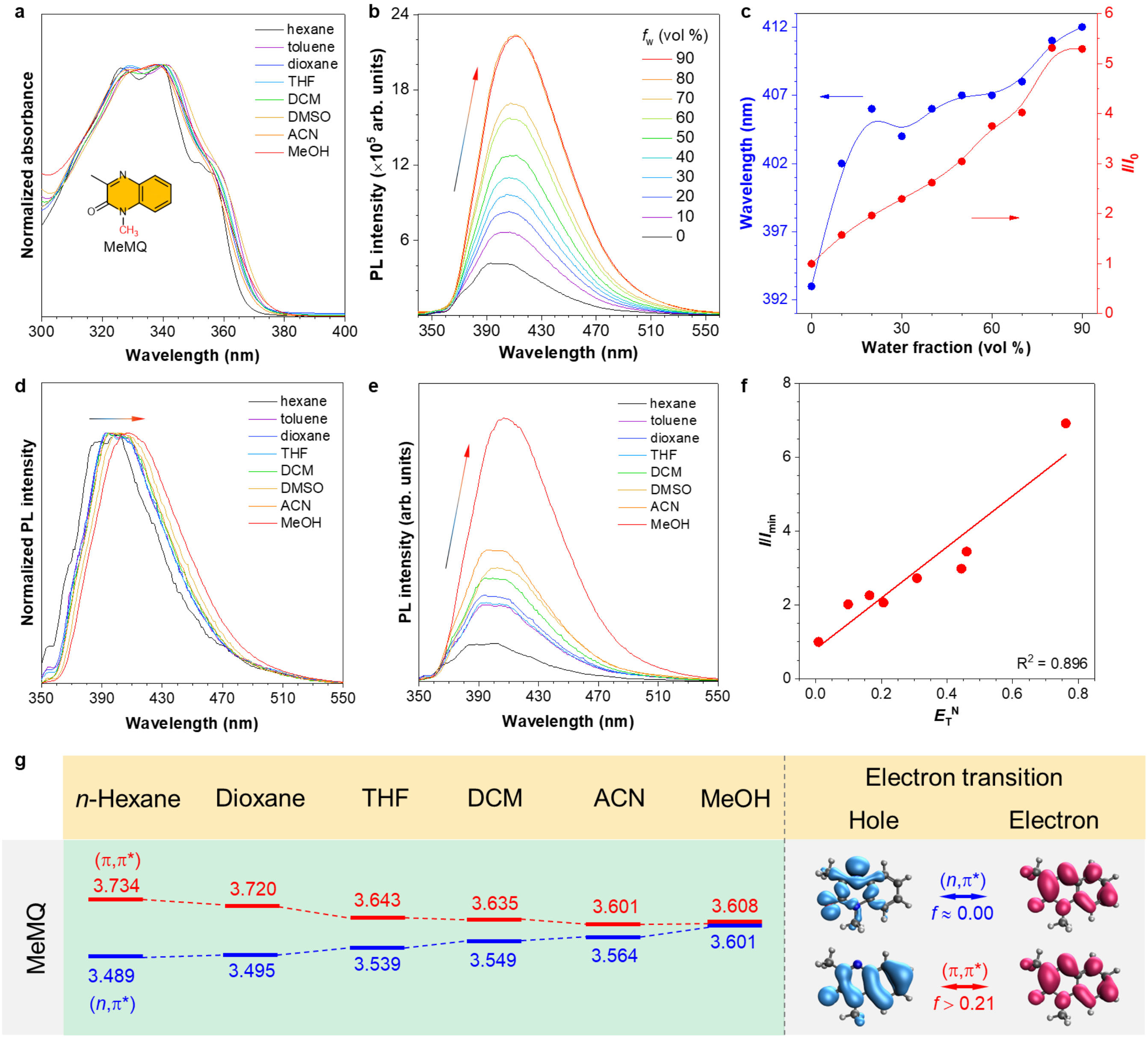

Supplementary Figure 23. The photophysical properties of methyl-substituted MQ (MeMQ). **a** The normalized absorption spectra in different solvents. Concentration = 10^{-5} M. **b** Photoluminescence (PL) spectra of MeMQ in THF/water mixtures with different water fractions (f_w). Concentration (c) = 10^{-5} M, excitation wavelength (λ_{ex}) = 320 nm. **c** Plots of relative PL intensity (I/I_0) and maximum emission wavelength versus f_w. I_0 = PL intensity at f_w = 0%. c = 10^{-5} M, λ_{ex} = 320 nm. **d** The normalized PL spectra of MeMQ in different solvents with increased polarity. **e** PL spectra of MeMQ in different solvents with increased polarity. **f** The relative PL intensity (I/I_{min}) of MeMQ in different solvents versus the normalized Reichardt's parameter (E_T^N). **g** the adiabatic energy levels of the lowest-lying (n,π*) and (π,π*) states in different solvents and hole-electron analysis.

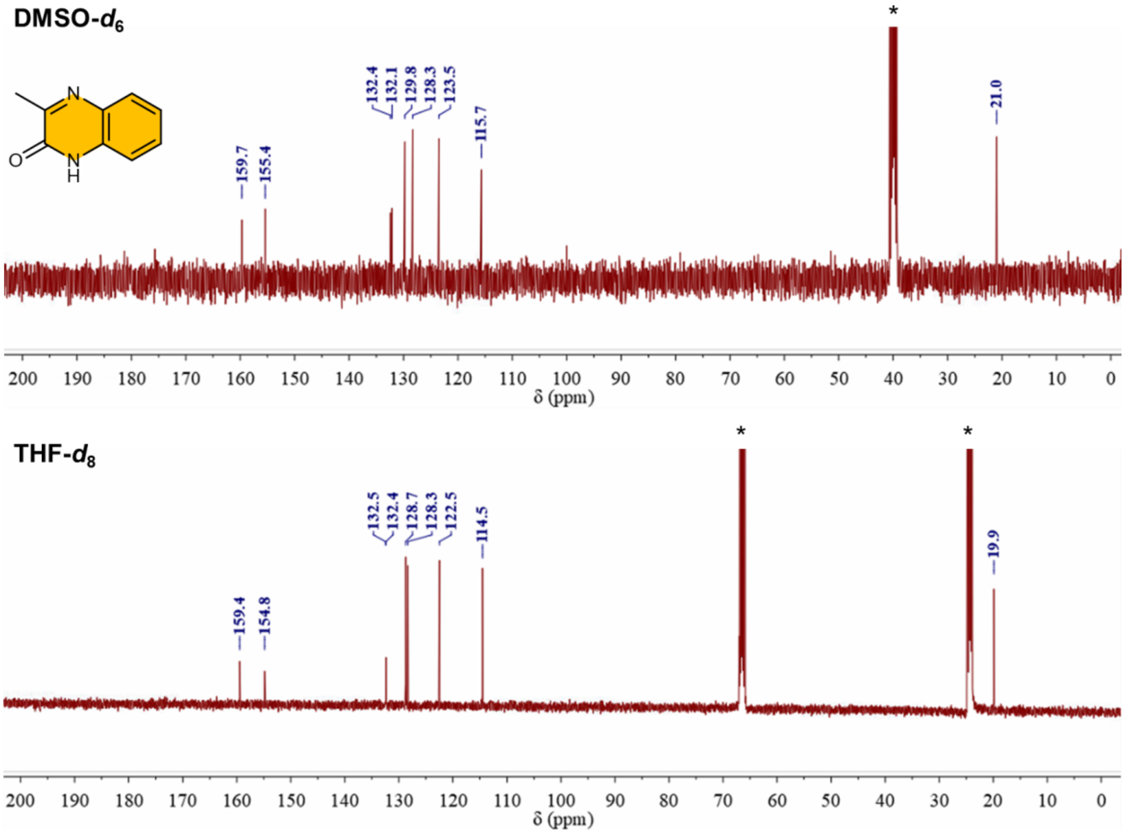

Supplementary Figure 24. Comparison of ^{13}C NMR spectra of MQ in DMSO-d_6 and THF-d_8.

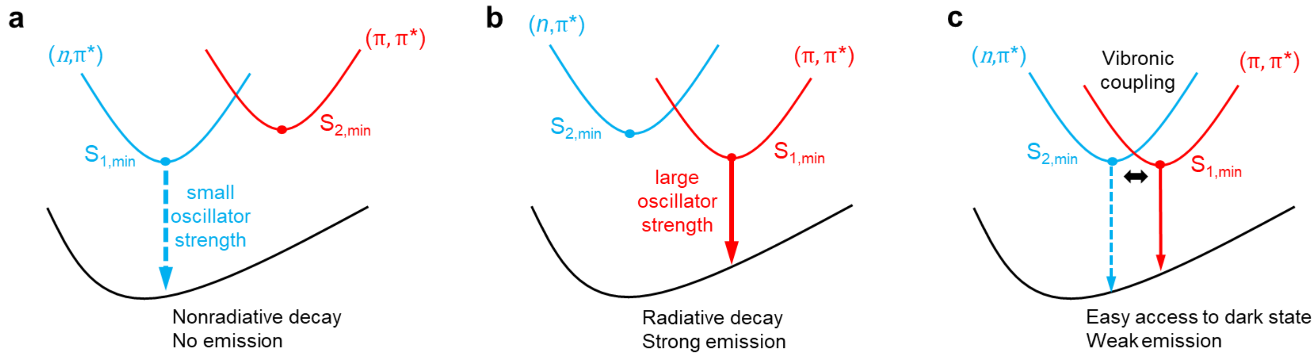

Supplementary Figure 25. The schematic Jablonski diagrams based on different energy levels of (n,π*) and (π,π*) states. **a** (n,π*) dominated lowest state. **b** (π,π*) dominated lowest state. **c** (n,π*) and (π,π*) states with close energy levels.

Supplementary Table 3. The calculated energy levels and hole-electron analysis of the lowest-lying (*n*,π*) and (π,π*) states of MQ in different solvents based on their corresponding optimized excited-state geometries.

Solvents	Electronic states	Adiabatic energy (eV)	Vertical energy (eV)	Oscillator strength (*f*)	Hole-electron analysis	
					Hole	Electron
n-Hexane	(*n*, π*)	3.4911	3.0953	0.0004	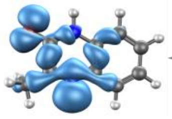	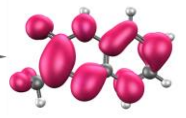
	(π, π*)	3.7871	3.5058	0.2131	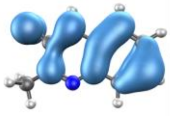	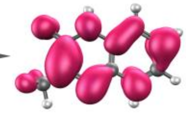
Dioxane	(*n*, π*)	3.4967	3.1028	0.0004	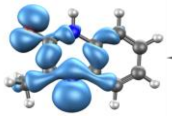	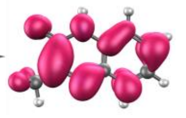
	(π, π*)	3.7780	3.4970	0.2212	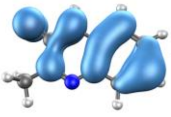	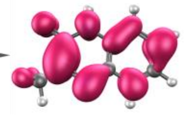
THF	(*n*, π*)	3.5222	3.0893	0.0006	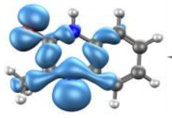	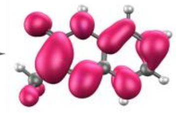
	(π, π*)	3.6909	3.4075	0.3117	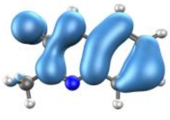	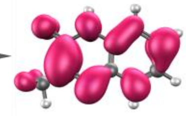
DCM	(*n*, π*)	3.5514	3.1667	0.0005	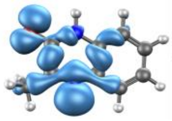	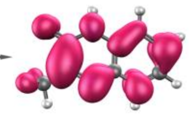
	(π, π*)	3.6801	3.3974	0.3263	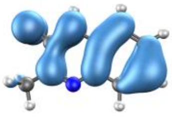	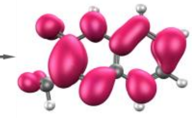
ACN	(*n*, π*)	3.5654	3.1895	0.0006	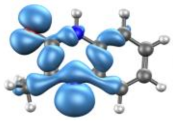	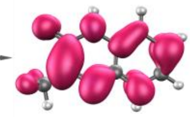
	(π, π*)	3.6466	3.3623	0.3622	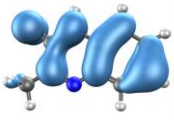	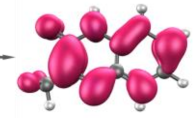
MeOH	(*n*, π*)	3.6093	3.1869	0.0007	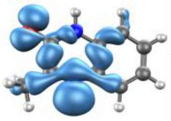	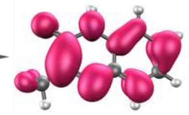
	(π, π*)	3.6503	3.3668	0.3935	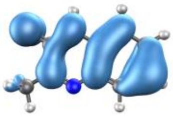	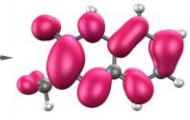

Supplementary Table 4. The calculated energy levels and hole-electron analysis of the lowest-lying (*n*,π*) and (π,π*) states of SQ in different solvents based on their corresponding optimized excited-state geometries.

Solvents	Electronic states	Adiabatic energy (eV)	Vertical energy (eV)	Oscillator strength (*f*)	Hole-electron analysis	
					Hole	Electron
n-Hexane	(π, π*)	2.8767	2.6527	1.0574	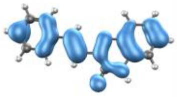	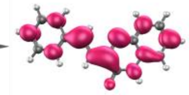
	(*n*, π*)	3.0801	2.6304	0.0003	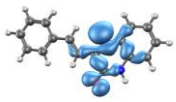	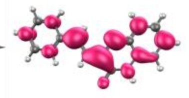
Dioxane	(π, π*)	2.8577	2.6317	1.0747	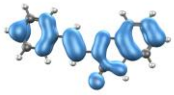	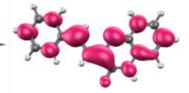
	(*n*, π*)	3.0845	2.6369	0.0003	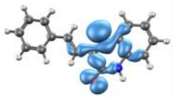	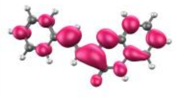
THF	(π, π*)	2.6950	2.4407	1.2175	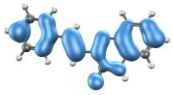	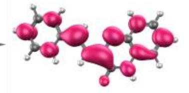
	(*n*, π*)	3.1097	2.6838	0.0003	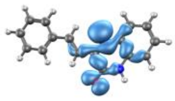	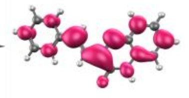
DCM	(π, π*)	2.6824	2.4257	1.2311	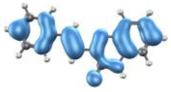	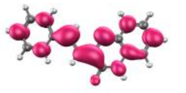
	(*n*, π*)	3.1205	2.6907	0.0003	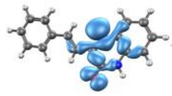	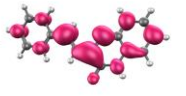
ACN	(π, π*)	2.6271	2.3595	1.2693	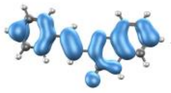	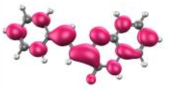
	(*n*, π*)	3.1252	2.7006	0.0004	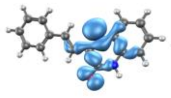	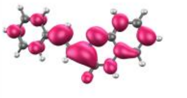
MeOH	(π, π*)	2.6370	2.3753	1.2888	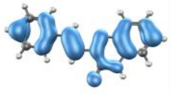	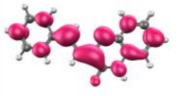
	(*n*, π*)	3.1915	2.7486	0.0004	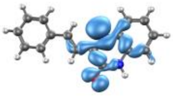	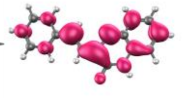

Supplementary Table 5. The calculated energy levels and hole-electron analysis of the lowest-lying (n,π*) and (π,π*) states of MeOSQ in different solvents based on their corresponding optimized excited-state geometries.

Solvents	Electronic states	Adiabatic energy (eV)	Vertical energy (eV)	Oscillator strength (f)	Hole-electron analysis	
					Hole	Electron
n-Hexane	(π, π*)	2.7724	2.5850	1.2121	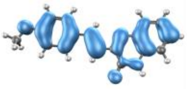	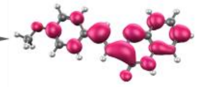
	(n, π*)	3.1196	2.6768	0.0003	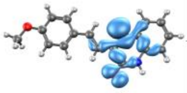	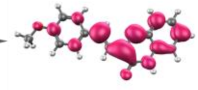
Dioxane	(π, π*)	2.7528	2.5628	1.2286	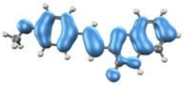	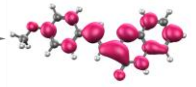
	(n, π*)	3.1244	2.6824	0.0003	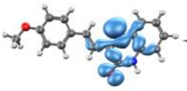	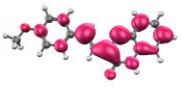
THF	(π, π*)	2.5847	2.3683	1.3699	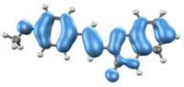	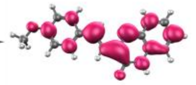
	(n, π*)	3.1550	2.7317	0.0003	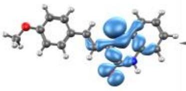	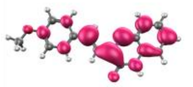
DCM	(π, π*)	2.5674	2.3487	1.3847	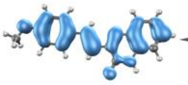	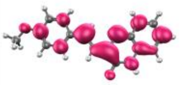
	(n, π*)	3.1656	2.7409	0.0003	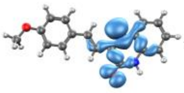	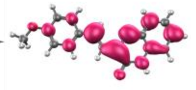
ACN	(π, π*)	2.5113	2.2810	1.4248	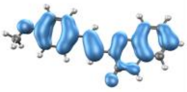	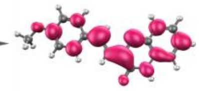
	(n, π*)	3.1726	2.7560	0.0004	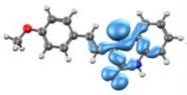	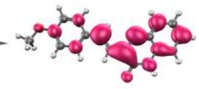
MeOH	(π, π*)	2.5052	2.2797	1.4307	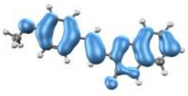	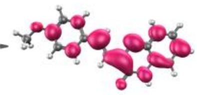
	(n, π*)	3.2319	2.7951	0.0004	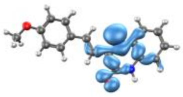	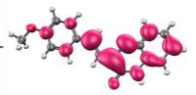

Supplementary Table 6. The calculated energy levels and hole-electron analysis of the lowest-lying (n,π*) and (π,π*) states of DPASQ in different solvents based on their corresponding optimized excited-state geometries.

Solvents	Electronic states	Adiabatic energy (eV)	Vertical energy (eV)	Oscillator strength (*f*)	Hole-electron analysis	
					Hole	Electron
n-Hexane	(π, π*)	2.4012	2.2650	1.1016	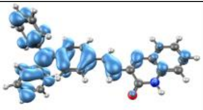	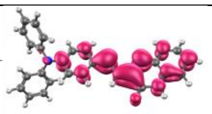
	(*n*, π*)	3.0965	2.6438	0.0002	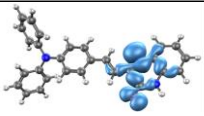	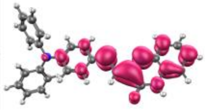
Dioxane	(π, π*)	2.3881	2.2547	1.1645	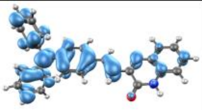	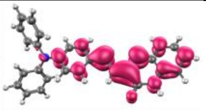
	(*n*, π*)	3.1013	2.6512	0.0002	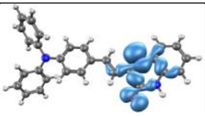	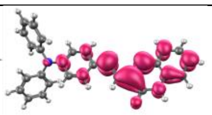
THF	(π, π*)	2.2704	2.1226	1.5242	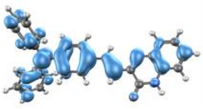	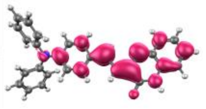
	(*n*, π*)	3.1313	2.6996	0.0003	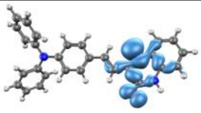	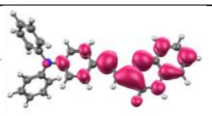
DCM	(π, π*)	2.2567	2.1068	1.5407	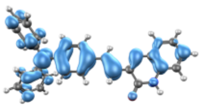	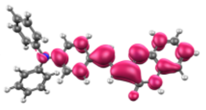
	(*n*, π*)	3.1430	2.7106	0.0004	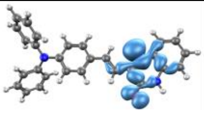	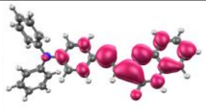
ACN	(π, π*)	2.2127	2.0508	1.6201	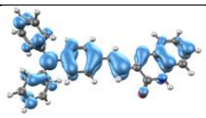	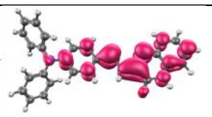
	(*n*, π*)	3.1497	2.7255	0.0004	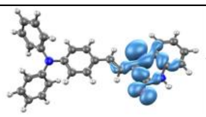	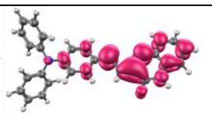
MeOH	(π, π*)	2.1857	2.0329	1.5653	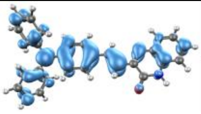	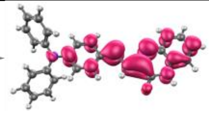
	(*n*, π*)	3.2158	2.7735	0.0004	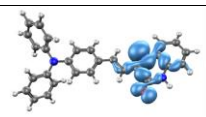	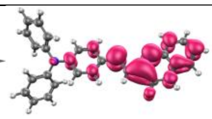

Supplementary Table 7. The calculated energy levels and hole-electron analysis of the lowest-lying (n,π*) and (π,π*) states of MeMQ in different solvents based on their corresponding optimized excited-state geometries.

Solvents	Electronic states	Adiabatic energy (eV)	Vertical energy (eV)	Oscillator strength (f)	Hole-electron analysis	
					Hole	Electron
n-Hexane	(n, π*)	3.4891	3.0792	0.0004	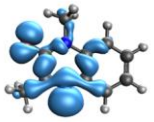	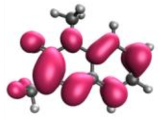
	(π, π*)	3.7345	3.4681	0.2055	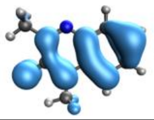	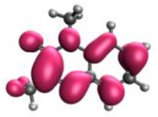
Dioxane	(n, π*)	3.4945	3.0867	0.0004	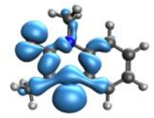	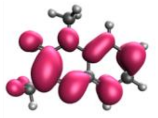
	(π, π*)	3.7199	3.4008	0.2179	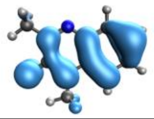	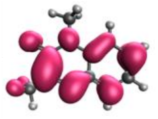
THF	(n, π*)	3.5388	3.1468	0.0005	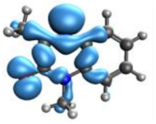	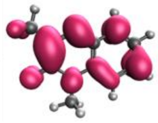
	(π, π*)	3.6428	3.3741	0.3020	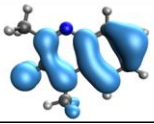	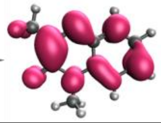
DCM	(n, π*)	3.5486	3.1523	0.0005	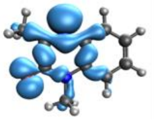	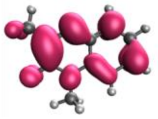
	(π, π*)	3.6346	3.3656	0.3156	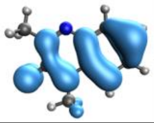	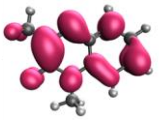
ACN	(n, π*)	3.5635	3.1747	0.0005	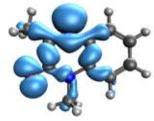	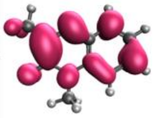
	(π, π*)	3.6013	3.3307	0.3518	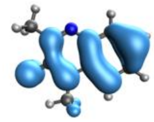	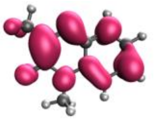
MeOH	(n, π*)	3.6014	3.1734	0.0007	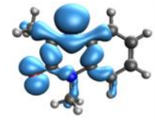	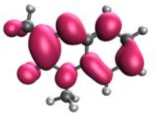
	(π, π*)	3.6083	3.3342	0.3822	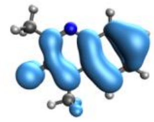	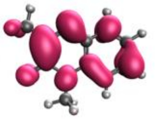

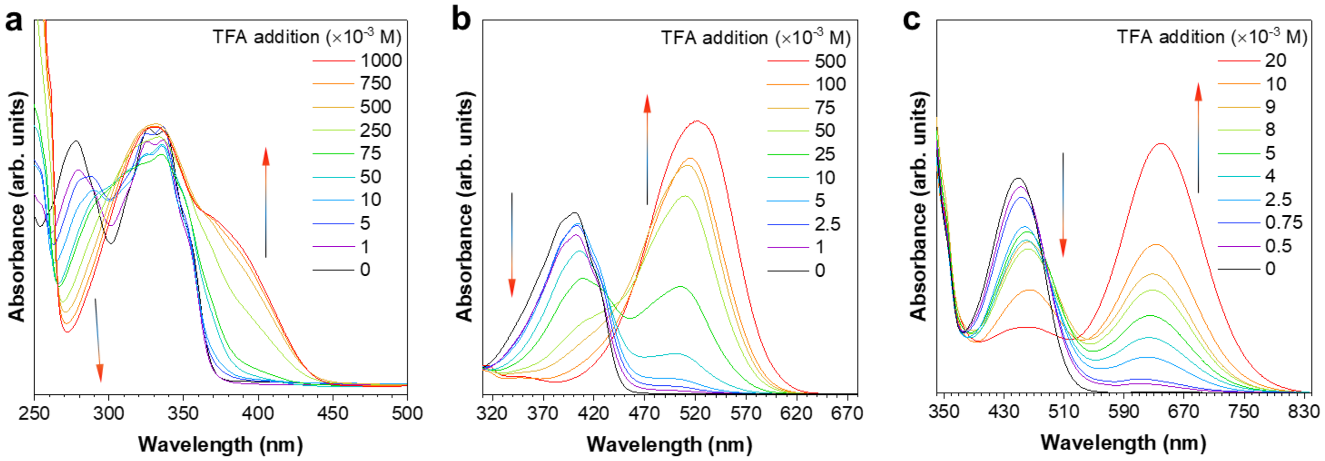

Supplementary Figure 26. Absorption spectra of (a) MQ, (b) MeOSQ, and (c) DPASQ solutions in DCM (2 mL) upon the addition of trifluoroacetic acid (TFA). The concentration of luminogens is 10^{-4} M.

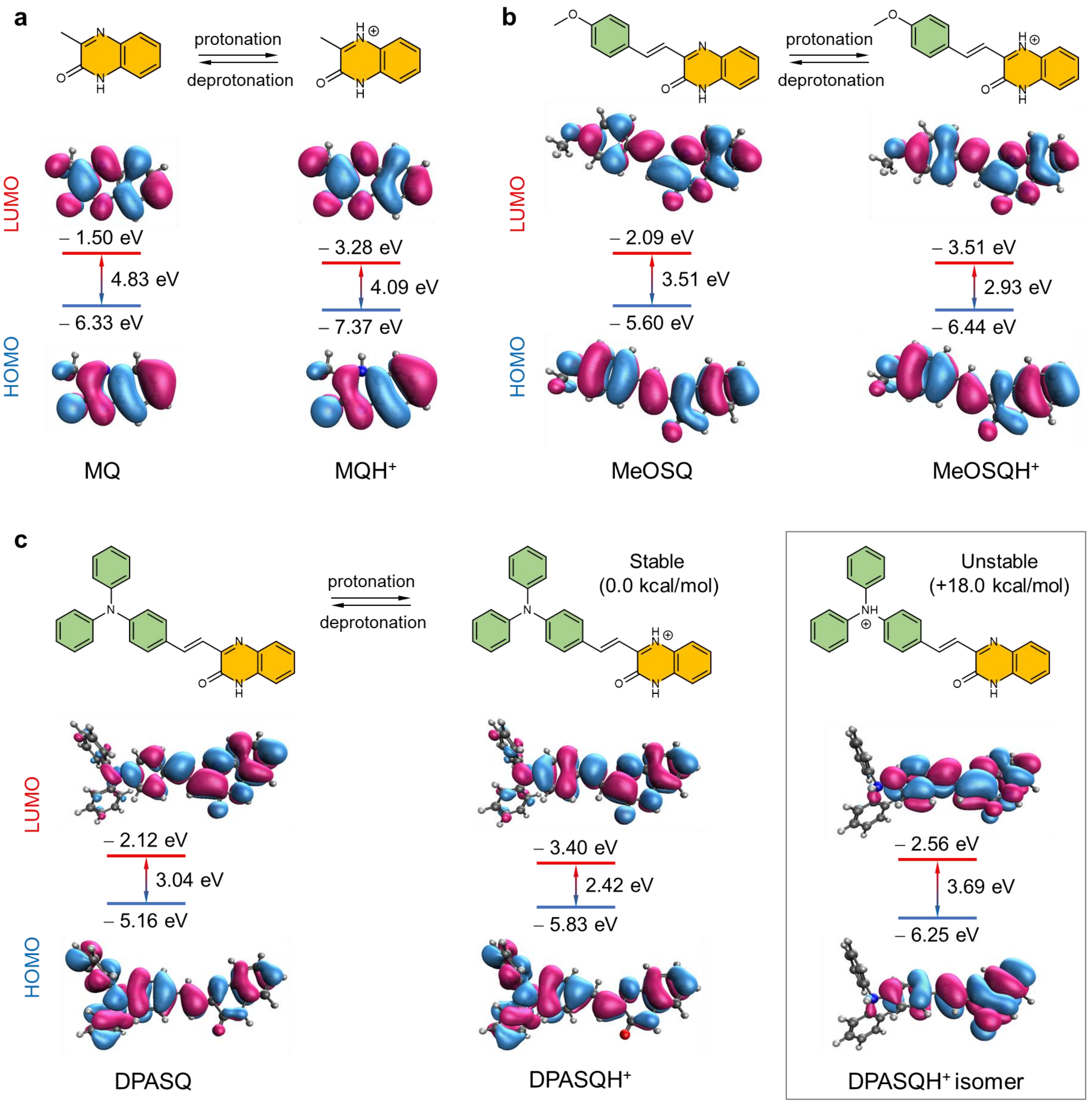

Supplementary Figure 27. Frontier molecular orbitals of deprotonated and protonated compounds of (a) MQ, (b) MeOSQ, and (c) DPASQ based on their optimized ground-state geometries. The compound in the bottom box is the unstable isomer of the protonated DPASQH$^+$, which displayed a higher electronic energy of 18.0 kcal/mol than that of the stable one.

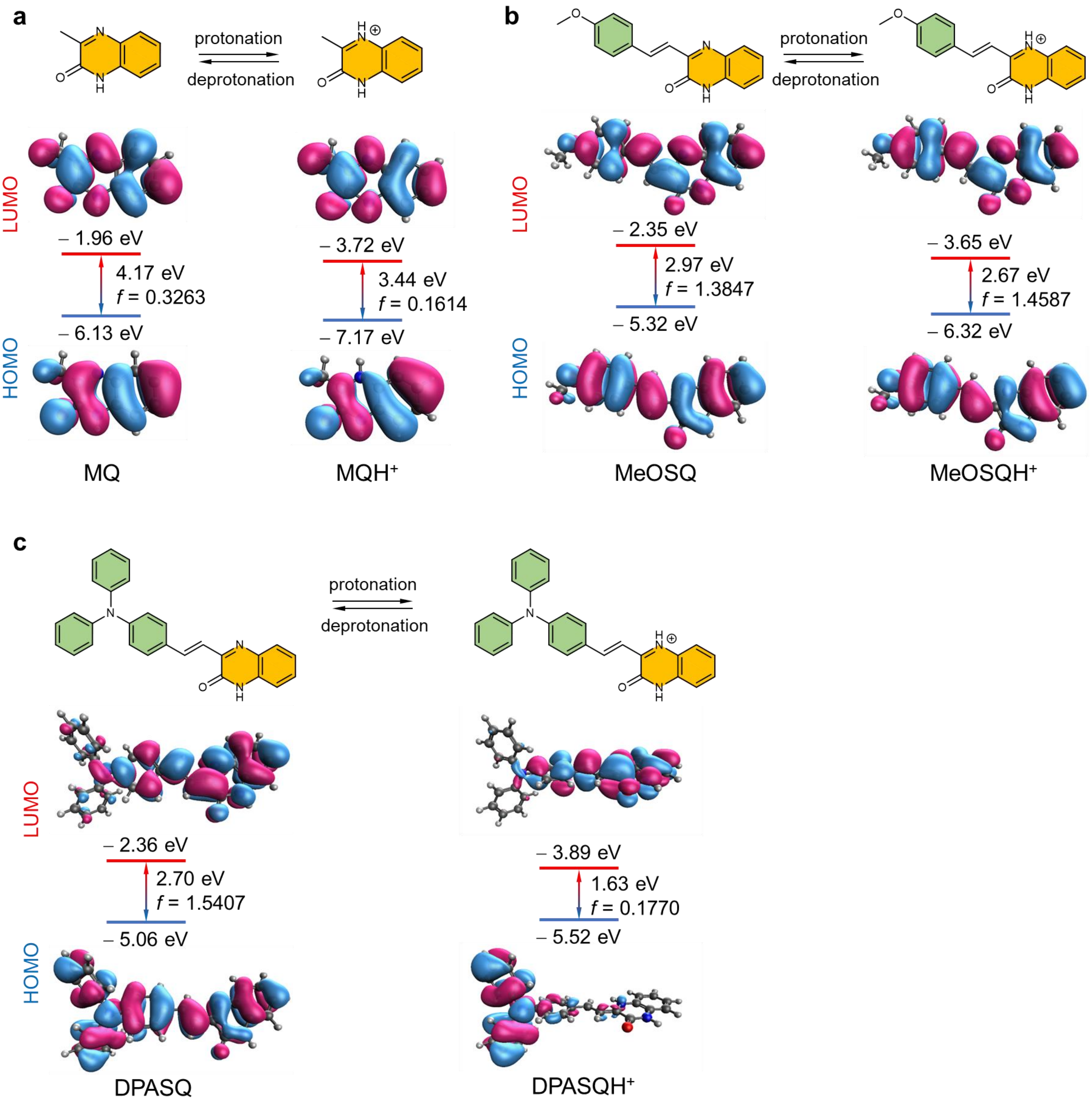

Supplementary Figure 28. Frontier molecular orbitals and oscillator strength (*f*) of the deprotonated and protonated compounds of (a) MQ, (b) MeOSQ, and (c) DPASQ based on their optimized excited-state geometries.

Cartesian Coordinates of the Optimized Molecular Geometry

Supplementary Table 8. Cartesian coordinates of optimized MQ in the ground state calculated at PBE0-D3/6-31G(d,p) level with the SMD model of *n*-hexane.

	X	Y	Z
C	3.017345	-1.353311	-0.000027
C	1.693406	-0.671762	-0.000084
C	1.728756	0.815336	-0.000097
N	0.485886	1.399784	-0.000051
C	-0.700166	0.701898	0.000005
C	-0.616855	-0.705839	0.000016
N	0.599587	-1.360479	-0.000031
C	-1.944384	1.340630	0.000052
C	-3.098967	0.575254	0.000106
C	-3.032723	-0.824171	0.000115
C	-1.801558	-1.455386	0.000072
O	2.761432	1.470793	-0.000095
H	3.602037	-1.052191	0.875908
H	2.880224	-2.435360	-0.000244
H	3.602297	-1.051827	-0.875657
H	0.472931	2.412315	-0.000048
H	-1.992555	2.426409	0.000045
H	-4.065119	1.070952	0.000142
H	-3.946122	-1.410321	0.000157
H	-1.712584	-2.537346	0.000078

Supplementary Table 9. Cartesian coordinates of optimized MQ in the excited (*n*,π*) state calculated at PBE0-D3/6-31G(d,p) level with the SMD model of *n*-hexane.

	X	Y	Z
C	3.061561	-1.385218	-0.000002
C	1.765954	-0.652928	-0.000091
C	1.744325	0.798504	-0.000226
N	0.493230	1.384061	-0.000096
C	-0.711770	0.695380	-0.000014
C	-0.649484	-0.734897	-0.000002
N	0.581311	-1.244567	-0.000003
C	-1.943547	1.328607	0.000066
C	-3.126371	0.578404	0.000123
C	-3.066225	-0.812298	0.000124
C	-1.846127	-1.476519	0.000073
O	2.782235	1.469257	-0.000014

H	3.657144	-1.123761	0.882143
H	2.882994	-2.462049	-0.000294
H	3.657464	-1.123308	-0.881790
H	0.481260	2.393382	-0.000064
H	-1.975947	2.415288	0.000065
H	-4.083450	1.088776	0.000172
H	-3.983261	-1.394046	0.000178
H	-1.785758	-2.559008	0.000092

Supplementary Table 10. Cartesian coordinates of optimized MQ in the excited (π,π*) state calculated at PBE0-D3/6-31G(d,p) level with the SMD model of *n*-hexane.

	X	Y	Z
C	3.067191	-1.325100	-0.000104
C	1.744450	-0.643304	-0.000009
C	1.721138	0.790874	0.000026
N	0.443282	1.399537	-0.000205
C	-0.709756	0.694636	-0.000143
C	-0.576609	-0.753906	-0.000082
N	0.616330	-1.392241	-0.000067
C	-1.970550	1.330820	-0.000039
C	-3.129767	0.570362	0.000063
C	-3.020996	-0.831115	0.000146
C	-1.775273	-1.475228	0.000116
O	2.691513	1.554560	0.000262
H	3.669925	-1.057048	0.877960
H	2.913263	-2.406413	-0.000467
H	3.670118	-1.056462	-0.877849
H	0.443582	2.413003	-0.000107
H	-2.008005	2.417450	-0.000117
H	-4.103051	1.047762	0.000081
H	-3.923443	-1.435777	0.000237
H	-1.710740	-2.558315	0.000224

Supplementary Table 11. Cartesian coordinates of optimized MQ in the ground state calculated at PBE0-D3/6-31G(d,p) level with the SMD model of dioxane.

	X	Y	Z
C	3.016622	-1.353714	-0.000031
C	1.693378	-0.671484	-0.000080
C	1.728536	0.815501	-0.000091
N	0.485866	1.399719	-0.000050

C	-0.699917	0.701648	0.000006
C	-0.616811	-0.705938	0.000017
N	0.599599	-1.360454	-0.000028
C	-1.943961	1.340534	0.000050
C	-3.098483	0.575379	0.000103
C	-3.032443	-0.823970	0.000113
C	-1.801532	-1.455331	0.000072
O	2.761381	1.471322	-0.000094
H	3.601774	-1.054072	0.876156
H	2.879017	-2.435798	-0.000245
H	3.602024	-1.053713	-0.875921
H	0.471520	2.412323	-0.000048
H	-1.992020	2.426355	0.000042
H	-4.064634	1.071223	0.000137
H	-3.945974	-1.410049	0.000154
H	-1.713346	-2.537440	0.000079

Supplementary Table 12. Cartesian coordinates of optimized MQ in the excited (*n*,π*) state calculated at PBE0-D3/6-31G(d,p) level with the SMD model of dioxane.

	X	Y	Z
C	3.060629	-1.385739	-0.000005
C	1.765861	-0.652518	-0.000090
C	1.744054	0.798654	-0.000218
N	0.493118	1.384253	-0.000092
C	-0.711468	0.695239	-0.000013
C	-0.649349	-0.734813	-0.000002
N	0.581430	-1.244894	-0.000004
C	-1.943261	1.328449	0.000065
C	-3.125885	0.578331	0.000120
C	-3.065884	-0.812248	0.000121
C	-1.845776	-1.476315	0.000071
O	2.782136	1.469908	-0.000013
H	3.656722	-1.125826	0.882321
H	2.881024	-2.462476	-0.000278
H	3.657023	-1.125405	-0.881998
H	0.479912	2.393661	-0.000059
H	-1.975701	2.415180	0.000065
H	-4.083001	1.088823	0.000169
H	-3.982966	-1.394061	0.000174
H	-1.785472	-2.558908	0.000089

Supplementary Table 13. Cartesian coordinates of optimized MQ in the excited (π,π*) state calculated at PBE0-D3/6-31G(d,p) level with the SMD model of dioxane.

	X	Y	Z
C	3.067191	-1.325100	-0.000104
C	1.744450	-0.643304	-0.000009
C	1.721138	0.790874	0.000026
N	0.443282	1.399537	-0.000205
C	-0.709756	0.694636	-0.000143
C	-0.576609	-0.753906	-0.000082
N	0.616330	-1.392241	-0.000067
C	-1.970550	1.330820	-0.000039
C	-3.129767	0.570362	0.000063
C	-3.020996	-0.831115	0.000146
C	-1.775273	-1.475228	0.000116
O	2.691513	1.554560	0.000262
H	3.669925	-1.057048	0.877960
H	2.913263	-2.406413	-0.000467
H	3.670118	-1.056462	-0.877849
H	0.443582	2.413003	-0.000107
H	-2.008005	2.417450	-0.000117
H	-4.103051	1.047762	0.000081
H	-3.923443	-1.435777	0.000237
H	-1.710740	-2.558315	0.000224

Supplementary Table 14. Cartesian coordinates of optimized MQ in the ground state calculated at PBE0-D3/6-31G(d,p) level with the SMD model of THF.

	X	Y	Z
C	3.017458	-1.352253	-0.000233
C	1.693600	-0.671056	0.000056
C	1.726144	0.816033	0.000563
N	0.486059	1.399868	0.000111
C	-0.698584	0.700365	0.000035
C	-0.617304	-0.707397	0.000176
N	0.599849	-1.361536	0.000128
C	-1.942305	1.341446	-0.000138
C	-3.097783	0.577252	-0.000105
C	-3.033552	-0.823307	0.000053
C	-1.803192	-1.456652	0.000175
O	2.762475	1.469767	-0.000500

H	3.601515	-1.053129	0.877072
H	2.883306	-2.435080	-0.000815
H	3.601674	-1.052160	-0.877097
H	0.463318	2.413669	-0.000143
H	-1.986660	2.427314	-0.000254
H	-4.063517	1.074221	-0.000214
H	-3.947963	-1.408368	0.000050
H	-1.719716	-2.539512	0.000248

Supplementary Table 15. Cartesian coordinates of optimized MQ in the excited (*n*,π*) state calculated at PBE0-D3/6-31G(d,p) level with the SMD model of THF.

	X	Y	Z
C	3.046820	-1.411381	-0.000113
C	1.770532	-0.647602	-0.000010
C	1.747962	0.805646	0.000007
N	0.494246	1.385555	0.000235
C	-0.707951	0.694875	0.000071
C	-0.648318	-0.734859	0.000093
N	0.586194	-1.239812	0.000080
C	-1.941443	1.328411	-0.000088
C	-3.123135	0.577510	-0.000140
C	-3.064410	-0.813968	-0.000034
C	-1.842667	-1.477725	0.000060
O	2.778534	1.490786	-0.000109
H	3.124661	-2.054629	0.885038
H	3.123769	-2.055779	-0.884486
H	3.878817	-0.704887	-0.000920
H	0.472299	2.396358	0.000305
H	-1.971638	2.415189	-0.000155
H	-4.080387	1.088475	-0.000266
H	-3.981523	-1.395998	-0.000037
H	-1.781684	-2.560659	0.000115

Supplementary Table 16. Cartesian coordinates of optimized MQ in the excited (π,π*) state calculated at PBE0-D3/6-31G(d,p) level with the SMD model of THF.

	X	Y	Z
C	-3.065823	-1.324143	-0.000486
C	-1.742954	-0.642475	0.000039
C	-1.719481	0.790864	0.000015
N	-0.443684	1.400489	-0.001447

C	0.707304	0.696204	-0.000999
C	0.579359	-0.756072	-0.000583
N	-0.613331	-1.394533	-0.000396
C	1.967171	1.334666	-0.000364
C	3.124944	0.574316	0.000276
C	3.021350	-0.830556	0.000820
C	1.780467	-1.477701	0.000655
O	-2.696630	1.548555	0.002087
H	-3.669131	-1.055712	-0.878703
H	-2.916474	-2.406475	-0.000223
H	-3.669864	-1.055315	0.877098
H	-0.433675	2.415347	-0.000542
H	2.000911	2.421141	-0.000848
H	4.098132	1.052702	0.000396
H	3.926604	-1.431046	0.001414
H	1.721614	-2.561391	0.001378

Supplementary Table 17. Cartesian coordinates of optimized MQ in the ground state calculated at PBE0-D3/6-31G(d,p) level with the SMD model of DCM.

	X	Y	Z
C	3.018075	-1.351918	-0.000047
C	1.694035	-0.670951	-0.000070
C	1.725011	0.815391	-0.000063
N	0.486256	1.399219	-0.000035
C	-0.698625	0.700043	0.000009
C	-0.617208	-0.707705	0.000020
N	0.600059	-1.361509	-0.000025
C	-1.942111	1.341712	0.000041
C	-3.097646	0.577573	0.000086
C	-3.033522	-0.823290	0.000099
C	-1.803334	-1.456994	0.000068
O	2.761506	1.470728	-0.000083
H	3.601838	-1.052835	0.877498
H	2.884109	-2.434744	-0.000202
H	3.602002	-1.052573	-0.877388
H	0.465352	2.413149	-0.000035
H	-1.985797	2.427549	0.000030
H	-4.063339	1.074575	0.000111
H	-3.948029	-1.408169	0.000133
H	-1.720449	-2.539904	0.000075

Supplementary Table 18. Cartesian coordinates of optimized MQ in the excited (n,π*) state calculated at PBE0-D3/6-31G(d,p) level with the SMD model of DCM.

	X	Y	Z
C	3.062350	-1.384230	-0.000042
C	1.766353	-0.651913	-0.000050
C	1.741093	0.798272	-0.000062
N	0.492626	1.385404	-0.000027
C	-0.710088	0.694817	0.000019
C	-0.649745	-0.735485	0.000035
N	0.582280	-1.247379	-0.000004
C	-1.942886	1.329077	0.000040
C	-3.125736	0.578836	0.000080
C	-3.067317	-0.812567	0.000094
C	-1.845693	-1.477123	0.000067
O	2.782015	1.469571	-0.000127
H	3.657399	-1.123967	0.883147
H	2.883815	-2.461238	-0.000252
H	3.657569	-1.123638	-0.883015
H	0.475584	2.396285	-0.000059
H	-1.971860	2.415860	0.000027
H	-4.082630	1.090456	0.000101
H	-3.984581	-1.394453	0.000123
H	-1.785741	-2.560140	0.000074

Supplementary Table 19. Cartesian coordinates of optimized MQ in the excited (π,π*) state calculated at PBE0-D3/6-31G(d,p) level with the SMD model of DCM.

	X	Y	Z
C	3.066286	-1.323069	-0.000057
C	1.742748	-0.642470	-0.000043
C	1.718489	0.789848	-0.000054
N	0.443743	1.400214	-0.000052
C	-0.706987	0.696324	-0.000009
C	-0.579676	-0.756260	0.000009
N	0.613253	-1.395241	-0.000013
C	-1.966903	1.335173	0.000027
C	-3.124425	0.574760	0.000076
C	-3.021465	-0.830491	0.000096
C	-1.780733	-1.477907	0.000069
O	2.696327	1.548058	-0.000044

H	3.669332	-1.053568	0.878084
H	2.918679	-2.405616	-0.000234
H	3.669457	-1.053279	-0.878018
H	0.436031	2.415088	-0.000042
H	-2.000102	2.421598	0.000008
H	-4.097618	1.053210	0.000097
H	-3.926990	-1.430532	0.000133
H	-1.722386	-2.561629	0.000091

Supplementary Table 20. Cartesian coordinates of optimized MQ in the ground state calculated at PBE0-D3/6-31G(d,p) level with the SMD model of ACN.

	X	Y	Z
C	3.017946	-1.351729	-0.000059
C	1.694031	-0.670736	-0.000055
C	1.724353	0.815769	-0.000011
N	0.486145	1.399283	-0.000023
C	-0.697995	0.699528	0.000013
C	-0.617285	-0.708282	0.000037
N	0.600225	-1.362003	-0.000012
C	-1.941265	1.341944	0.000026
C	-3.097117	0.578261	0.000071
C	-3.033795	-0.822976	0.000099
C	-1.803855	-1.457359	0.000082
O	2.761682	1.470488	-0.000138
H	3.601318	-1.053137	0.877931
H	2.884757	-2.434724	-0.000290
H	3.601509	-1.052751	-0.877783
H	0.461998	2.413653	-0.000053
H	-1.983657	2.427743	0.000003
H	-4.062563	1.075805	0.000082
H	-3.948608	-1.407426	0.000131
H	-1.722912	-2.540548	0.000097

Supplementary Table 21. Cartesian coordinates of optimized MQ in the excited (n,π*) state calculated at PBE0-D3/6-31G(d,p) level with the SMD model of ACN.

	X	Y	Z
C	3.062215	-1.383446	-0.000062
C	1.766089	-0.651304	-0.000019
C	1.739313	0.798912	0.000060
N	0.492018	1.386359	0.000014

C	-0.709592	0.694537	0.000047
C	-0.648886	-0.735562	0.000080
N	0.582989	-1.249941	0.000006
C	-1.942606	1.329200	0.000011
C	-3.125310	0.578732	0.000051
C	-3.067067	-0.812887	0.000090
C	-1.844869	-1.477183	0.000087
O	2.781394	1.469854	-0.000266
H	3.657482	-1.124371	0.883389
H	2.883098	-2.460407	-0.000379
H	3.657676	-1.123871	-0.883231
H	0.472237	2.397759	-0.000085
H	-1.970838	2.415950	-0.000026
H	-4.082257	1.090477	0.000043
H	-3.984286	-1.394954	0.000106
H	-1.785034	-2.560342	0.000094

Supplementary Table 22. Cartesian coordinates of optimized MQ in the excited (π,π*) state calculated at PBE0-D3/6-31G(d,p) level with the SMD model of ACN.

	X	Y	Z
C	3.064841	-1.323308	-0.000043
C	1.741960	-0.642027	-0.000050
C	1.718049	0.790319	-0.000065
N	0.444000	1.400864	0.000005
C	-0.705901	0.697032	0.000038
C	-0.580535	-0.756884	0.000055
N	0.611852	-1.395887	0.000020
C	-1.965230	1.336469	0.000040
C	-3.122372	0.576133	0.000074
C	-3.021484	-0.830186	0.000089
C	-1.782672	-1.478590	0.000071
O	2.698691	1.545453	-0.000194
H	3.668625	-1.054784	0.878122
H	2.917922	-2.406077	-0.000279
H	3.668820	-1.054400	-0.877951
H	0.431450	2.416393	-0.000054
H	-1.997305	2.422794	0.000028
H	-4.095481	1.055002	0.000088
H	-3.928091	-1.428590	0.000111
H	-1.726373	-2.562535	0.000072

Supplementary Table 23. Cartesian coordinates of optimized MQ in the ground state calculated at PBE0-D3/6-31G(d,p) level with the SMD model of MeOH.

	X	Y	Z
C	3.019081	-1.346273	-0.000055
C	1.693364	-0.670506	-0.000007
C	1.717365	0.811133	-0.000147
N	0.487379	1.396980	-0.000054
C	-0.698095	0.697831	0.000000
C	-0.616950	-0.709453	-0.000004
N	0.599183	-1.362707	0.000008
C	-1.939388	1.342795	0.000048
C	-3.095127	0.580058	0.000078
C	-3.032452	-0.821525	0.000072
C	-1.804388	-1.457742	0.000033
O	2.761126	1.469062	0.000013
H	3.601349	-1.046577	0.878788
H	2.890963	-2.429832	-0.000086
H	3.601348	-1.046517	-0.878879
H	0.465839	2.412089	-0.000027
H	-1.980715	2.428657	0.000052
H	-4.060499	1.077872	0.000110
H	-3.948089	-1.404911	0.000103
H	-1.725595	-2.541084	0.000038

Supplementary Table 24. Cartesian coordinates of optimized MQ in the excited (n,π*) state calculated at PBE0-D3/6-31G(d,p) level with the SMD model of MeOH.

	X	Y	Z
C	-3.059862	-1.389719	0.000025
C	-1.768071	-0.650863	0.000070
C	-1.743966	0.793833	0.000058
N	-0.495249	1.382404	-0.000022
C	0.708364	0.695208	-0.000065
C	0.655234	-0.736777	-0.000050
N	-0.580163	-1.237884	0.000032
C	1.941366	1.329202	-0.000071
C	3.124861	0.581030	-0.000094
C	3.070300	-0.810100	-0.000078
C	1.849720	-1.477400	-0.000038
O	-2.787522	1.469196	0.000198

H	-3.653905	-1.129903	-0.884039
H	-2.880126	-2.466415	0.000120
H	-3.654029	-1.129763	0.883962
H	-0.479842	2.394316	0.000077
H	1.967792	2.416091	-0.000077
H	4.080727	1.094923	-0.000121
H	3.988596	-1.390439	-0.000092
H	1.791170	-2.560507	-0.000013

Supplementary Table 25. Cartesian coordinates of optimized MQ in the excited (π,π*) state calculated at PBE0-D3/6-31G(d,p) level with the SMD model of MeOH.

	X	Y	Z
C	-3.064897	-1.316783	0.000054
C	-1.738851	-0.641531	0.000044
C	-1.713404	0.785130	0.000065
N	-0.443010	1.402070	0.000130
C	0.705083	0.696479	0.000069
C	0.581423	-0.756036	0.000064
N	-0.612922	-1.399241	0.000076
C	1.965879	1.336140	-0.000022
C	3.121741	0.576158	-0.000096
C	3.021614	-0.829476	-0.000114
C	1.780182	-1.476927	-0.000059
O	-2.702145	1.540132	-0.000148
H	-3.665148	-1.042540	-0.878586
H	-2.925837	-2.400324	0.000109
H	-3.665187	-1.042450	0.878637
H	-0.432883	2.417395	-0.000020
H	1.998353	2.422463	0.000001
H	4.094916	1.055254	-0.000142
H	3.927706	-1.428635	-0.000175
H	1.724133	-2.560935	-0.000106

Supplementary Table 26. Cartesian coordinates of optimized SQ in the ground state calculated at PBE0-D3/6-31G(d,p) level with the SMD model of *n*-hexane.

	X	Y	Z
O	0.826998	2.946950	-0.002572
N	2.698820	1.661804	-0.001379
H	3.247075	2.513275	-0.002126
C	4.725477	0.320972	-0.000274

H	5.342851	1.215511	-0.000994
C	1.337399	1.835048	-0.001569
N	1.142362	-0.613939	0.000595
C	0.570355	0.555654	-0.000423
C	3.119204	-1.973423	0.001623
H	2.469745	-2.843336	0.002344
C	-0.875469	0.692010	-0.000489
H	-1.238595	1.714712	-0.001567
C	3.333088	0.440946	-0.000361
C	-5.256775	-1.575219	-0.003857
H	-5.778302	-2.528061	-0.007087
C	5.297384	-0.941085	0.000739
H	6.379159	-1.034782	0.000800
C	-1.710971	-0.365188	0.000368
H	-1.257296	-1.355949	0.000700
C	-5.976484	-0.383637	-0.000088
C	2.510539	-0.707556	0.000610
C	-3.909138	0.863931	0.004549
H	-3.395841	1.820887	0.008373
C	-5.295668	0.834769	0.004181
H	-5.852815	1.767329	0.007379
C	-3.166827	-0.330111	0.000524
C	4.496707	-2.091591	0.001678
H	4.959308	-3.073396	0.002451
C	-3.867363	-1.546868	-0.003454
H	-3.305707	-2.477830	-0.006439
H	-7.062589	-0.400929	-0.000321

Supplementary Table 27. Cartesian coordinates of optimized SQ in the excited (π,π*) state calculated at PBE0-D3/6-31G(d,p) level with the SMD model of *n*-hexane.

	X	Y	Z
O	0.880891	2.992667	-0.001377
N	2.709225	1.661066	-0.000077
H	3.267733	2.505875	-0.000155
C	4.703877	0.292965	0.000768
H	5.331011	1.181135	0.000970
C	1.334791	1.852885	-0.000614
N	1.112259	-0.621988	-0.000547
C	0.545664	0.615319	-0.000736
C	3.068139	-1.997147	0.000167

H	2.414371	-2.863725	-0.000099
C	-0.856010	0.736747	-0.000720
H	-1.247247	1.749203	-0.000762
C	3.314226	0.435913	0.000296
C	-5.223250	-1.583282	-0.001667
H	-5.748875	-2.534060	-0.003205
C	5.263203	-0.974893	0.001000
H	6.343195	-1.085071	0.001411
C	-1.700515	-0.367633	-0.000834
H	-1.219053	-1.343495	-0.001647
C	-5.946351	-0.385313	0.000673
C	2.445939	-0.718004	0.000011
C	-3.881355	0.869449	0.002408
H	-3.370680	1.827453	0.004359
C	-5.262858	0.835717	0.002754
H	-5.822440	1.767051	0.004717
C	-3.123381	-0.334699	-0.000153
C	4.438409	-2.120258	0.000685
H	4.893414	-3.106317	0.000839
C	-3.841637	-1.560894	-0.002049
H	-3.279865	-2.491686	-0.003912
H	-7.032424	-0.402473	0.000940

Supplementary Table 28. Cartesian coordinates of optimized SQ in the excited (n,π*) state calculated at PBE0-D3/6-31G(d,p) level with the SMD model of n-hexane.

	X	Y	Z
O	0.817496	2.948098	-0.000928
N	2.688577	1.656853	0.000227
H	3.237630	2.505709	0.000349
C	4.732644	0.324887	0.000823
H	5.333169	1.231109	0.001163
C	1.337700	1.842402	-0.000613
N	1.228297	-0.519526	-0.000565
C	0.507466	0.587252	-0.000710
C	3.163121	-2.001650	-0.000236
H	2.539474	-2.888468	-0.000674
C	-0.894040	0.686808	-0.000458
H	-1.268542	1.704600	-0.000485
C	3.350746	0.433176	0.000472
C	-5.290134	-1.577296	-0.001459

H	-5.816917	-2.527771	-0.002761
C	5.336676	-0.932751	0.000809
H	6.418624	-1.007924	0.001192
C	-1.739914	-0.394362	-0.000396
H	-1.296715	-1.388373	-0.000846
C	-6.006724	-0.381529	0.000368
C	2.543240	-0.740136	-0.000040
C	-3.926119	0.850424	0.001941
H	-3.411619	1.806863	0.003539
C	-5.312088	0.829506	0.002079
H	-5.860455	1.767781	0.003620
C	-3.181755	-0.349014	-0.000027
C	4.547642	-2.082864	0.000276
H	5.018142	-3.061370	0.000242
C	-3.902870	-1.561241	-0.001651
H	-3.351741	-2.498786	-0.003135
H	-7.092683	-0.391106	0.000516

Supplementary Table 29. Cartesian coordinates of optimized SQ in the ground state calculated at PBE0-D3/6-31G(d,p) level with the SMD model of dioxane.

	X	Y	Z
O	0.826878	2.947189	-0.007796
N	2.698527	1.661701	-0.004202
H	3.247983	2.512517	-0.006469
C	4.724745	0.321008	-0.000855
H	5.342086	1.215619	-0.003053
C	1.337408	1.834981	-0.004774
N	1.142017	-0.613971	0.001811
C	0.570268	0.555832	-0.001296
C	3.118786	-1.973433	0.004933
H	2.469841	-2.843831	0.007131
C	-0.875448	0.691651	-0.001453
H	-1.239691	1.713924	-0.004770
C	3.332426	0.440822	-0.001105
C	-5.256179	-1.574899	-0.011752
H	-5.777873	-2.527683	-0.021585
C	5.296635	-0.940860	0.002218
H	6.378484	-1.034496	0.002390
C	-1.710699	-0.365639	0.001221
H	-1.257659	-1.356713	0.002297

C	-5.975670	-0.383366	-0.000335
C	2.510108	-0.707650	0.001858
C	-3.908318	0.863861	0.013837
H	-3.395268	1.820966	0.025473
C	-5.294755	0.834793	0.012660
H	-5.851859	1.767417	0.022352
C	-3.166315	-0.330208	0.001647
C	4.496140	-2.091393	0.005083
H	4.958862	-3.073217	0.007426
C	-3.866867	-1.546781	-0.010468
H	-3.305493	-2.477940	-0.019512
H	-7.061848	-0.400504	-0.001082

Supplementary Table 30. Cartesian coordinates of optimized SQ in the excited (π,π*) state calculated at PBE0-D3/6-31G(d,p) level with the SMD model of dioxane.

	X	Y	Z
O	0.879299	2.992084	-0.004369
N	2.708164	1.661116	-0.000836
H	3.267317	2.505619	-0.000723
C	4.703066	0.294149	0.002751
H	5.329698	1.182709	0.003460
C	1.334121	1.852398	-0.003436
N	1.112677	-0.622971	-0.002012
C	0.545396	0.614559	-0.002954
C	3.069160	-1.996984	0.000768
H	2.416202	-2.864270	-0.000012
C	-0.855752	0.735000	-0.002831
H	-1.247946	1.747077	-0.003460
C	3.313561	0.436225	0.000736
C	-5.223320	-1.582615	-0.003625
H	-5.749479	-2.533151	-0.007435
C	5.263097	-0.973297	0.003836
H	6.343236	-1.082797	0.005500
C	-1.700700	-0.369202	-0.002558
H	-1.220809	-1.345808	-0.004486
C	-5.945685	-0.384337	0.002631
C	2.446148	-0.718079	-0.000230
C	-3.880226	0.869464	0.006469
H	-3.369651	1.827568	0.011444
C	-5.261633	0.836276	0.007801

H	-5.820827	1.767895	0.013071
C	-3.123042	-0.335100	-0.000393
C	4.439240	-2.119205	0.002793
H	4.895011	-3.104978	0.003616
C	-3.841836	-1.560989	-0.005044
H	-3.280748	-2.492227	-0.010028
H	-7.031840	-0.400892	0.003658

Supplementary Table 31. Cartesian coordinates of optimized SQ in the excited (n,π*) state calculated at PBE0-D3/6-31G(d,p) level with the SMD model of dioxane.

	X	Y	Z
O	0.818999	2.949815	-0.002117
N	2.689047	1.656790	0.000149
H	3.239928	2.504612	0.000364
C	4.731556	0.323250	0.001636
H	5.332845	1.229017	0.002309
C	1.338460	1.843435	-0.001517
N	1.226968	-0.518521	-0.001487
C	0.507636	0.589307	-0.001646
C	3.160428	-2.002031	-0.000521
H	2.536170	-2.888519	-0.001407
C	-0.893935	0.688377	-0.001002
H	-1.269684	1.705713	-0.000935
C	3.349778	0.432638	0.000795
C	-5.288138	-1.577806	0.000070
H	-5.814487	-2.528607	-0.000395
C	5.334461	-0.934734	0.001736
H	6.416444	-1.010681	0.002614
C	-1.738745	-0.393378	-0.000657
H	-1.295112	-1.387254	-0.001108
C	-6.005274	-0.382507	0.001263
C	2.541683	-0.740163	-0.000243
C	-3.925343	0.850417	0.001296
H	-3.411660	1.807388	0.001913
C	-5.311244	0.828742	0.001871
H	-5.860125	1.766803	0.002849
C	-3.180531	-0.348614	0.000023
C	4.544788	-2.084257	0.000650
H	5.014645	-3.063151	0.000687
C	-3.900947	-1.561103	-0.000556

H	-3.349557	-2.498560	-0.001514
H	-7.091305	-0.392600	0.001743

Supplementary Table 32. Cartesian coordinates of optimized SQ in the ground state calculated at PBE0-D3/6-31G(d,p) level with the SMD model of THF.

	X	Y	Z
O	0.819504	2.941845	0.000122
N	2.696738	1.662577	0.000321
H	3.251451	2.511807	0.000641
C	4.725749	0.328138	0.000453
H	5.338149	1.225953	0.000776
C	1.337294	1.831059	0.000029
N	1.145605	-0.618206	-0.000511
C	0.571786	0.551214	-0.000375
C	3.126421	-1.974084	-0.000400
H	2.482493	-2.848659	-0.000726
C	-0.875122	0.684274	-0.000341
H	-1.242299	1.705717	-0.000403
C	3.332132	0.442913	0.000180
C	-5.261997	-1.572727	0.000541
H	-5.785879	-2.524508	0.000893
C	5.301303	-0.932238	0.000298
H	6.383589	-1.022546	0.000512
C	-1.712706	-0.371950	-0.000151
H	-1.267817	-1.366782	-0.000043
C	-5.979229	-0.378796	0.000247
C	2.514185	-0.708912	-0.000248
C	-3.908118	0.865250	-0.000366
H	-3.393280	1.821705	-0.000736
C	-5.295460	0.838792	-0.000210
H	-5.850891	1.772620	-0.000454
C	-3.169104	-0.331599	-0.000056
C	4.504439	-2.086875	-0.000129
H	4.970955	-3.067092	-0.000242
C	-3.871833	-1.547808	0.000386
H	-3.311772	-2.479909	0.000615
H	-7.065582	-0.393570	0.000368

Supplementary Table 33. Cartesian coordinates of optimized SQ in the excited (π,π*) state calculated at PBE0-D3/6-31G(d,p) level with the SMD model of THF.

	X	Y	Z
O	0.863238	2.981453	-0.000095
N	2.701443	1.662908	-0.000014
H	3.263727	2.507102	-0.000035
C	4.703581	0.309348	0.000034
H	5.322162	1.203293	0.000027
C	1.329437	1.845882	-0.000037
N	1.121747	-0.634402	0.000001
C	0.544015	0.602366	-0.000024
C	3.087022	-1.996883	0.000049
H	2.442638	-2.870997	0.000052
C	-0.852479	0.719744	-0.000031
H	-1.246403	1.731320	-0.000044
C	3.313453	0.441230	0.000015
C	-5.234288	-1.577494	0.000021
H	-5.765890	-2.525089	0.000040
C	5.272876	-0.954841	0.000061
H	6.353974	-1.056454	0.000077
C	-1.707605	-0.383184	-0.000018
H	-1.240149	-1.365738	-0.000006
C	-5.950855	-0.374313	0.000007
C	2.453725	-0.720327	0.000023
C	-3.878507	0.871264	-0.000030
H	-3.364006	1.827374	-0.000051
C	-5.260152	0.844065	-0.000019
H	-5.815417	1.778131	-0.000030
C	-3.126273	-0.339198	-0.000014
C	4.457620	-2.108489	0.000069
H	4.921530	-3.090575	0.000089
C	-3.852778	-1.563610	0.000010
H	-3.296007	-2.497541	0.000021
H	-7.037140	-0.385348	0.000017

Supplementary Table 34. Cartesian coordinates of optimized SQ in the excited (n,π*) state calculated at PBE0-D3/6-31G(d,p) level with the SMD model of THF.

	X	Y	Z
O	0.818746	2.950585	-0.000389
N	2.691893	1.658693	-0.000024
H	3.251009	2.503360	0.000012
C	4.730772	0.321770	0.000265

H	5.331117	1.228020	0.000345
C	1.343807	1.845153	-0.000293
N	1.223724	-0.519309	-0.000205
C	0.511016	0.592372	-0.000284
C	3.155821	-2.003737	-0.000015
H	2.529711	-2.889325	-0.000129
C	-0.892391	0.691338	-0.000193
H	-1.271376	1.707926	-0.000202
C	3.347731	0.432781	0.000120
C	-5.286697	-1.579040	0.000081
H	-5.812076	-2.530589	0.000066
C	5.330968	-0.937410	0.000317
H	6.413187	-1.014441	0.000458
C	-1.735496	-0.392271	-0.000104
H	-1.292738	-1.386865	-0.000139
C	-6.005695	-0.383791	0.000200
C	2.539917	-0.740148	-0.000013
C	-3.925093	0.851810	0.000125
H	-3.412407	1.809631	0.000157
C	-5.312129	0.828636	0.000220
H	-5.861855	1.766424	0.000316
C	-3.178991	-0.347336	-0.000001
C	4.540587	-2.087935	0.000175
H	5.009923	-3.067265	0.000210
C	-3.898496	-1.561253	-0.000022
H	-3.345673	-2.498017	-0.000117
H	-7.091889	-0.394870	0.000278

Supplementary Table 35. Cartesian coordinates of optimized SQ in the ground state calculated at PBE0-D3/6-31G(d,p) level with the SMD model of DCM.

	X	Y	Z
O	0.822145	2.943647	0.000082
N	2.697693	1.662037	0.000135
H	3.254227	2.510228	0.000265
C	4.725811	0.326636	0.000186
H	5.338333	1.224323	0.000322
C	1.339302	1.831434	0.000020
N	1.144981	-0.616894	-0.000217
C	0.572074	0.553239	-0.000156
C	3.124714	-1.975078	-0.000171

H	2.480566	-2.849497	-0.000307
C	-0.874955	0.687188	-0.000134
H	-1.242459	1.708607	-0.000133
C	3.332127	0.441824	0.000072
C	-5.260624	-1.574248	0.000253
H	-5.783223	-2.526736	0.000423
C	5.300514	-0.934137	0.000121
H	6.382731	-1.025205	0.000211
C	-1.712654	-0.369172	-0.000078
H	-1.267829	-1.364015	-0.000056
C	-5.979464	-0.381089	0.000110
C	2.513357	-0.709238	-0.000108
C	-3.909771	0.865926	-0.000186
H	-3.395994	1.822959	-0.000370
C	-5.297253	0.837566	-0.000112
H	-5.853982	1.770614	-0.000235
C	-3.169235	-0.330130	-0.000032
C	4.502717	-2.088487	-0.000057
H	4.968748	-3.068937	-0.000103
C	-3.870368	-1.547474	0.000181
H	-3.308950	-2.478764	0.000292
H	-7.065796	-0.397317	0.000166

Supplementary Table 36. Cartesian coordinates of optimized SQ in the excited (π,π*) state calculated at PBE0-D3/6-31G(d,p) level with the SMD model of DCM.

	X	Y	Z
O	0.865559	2.983296	-0.000050
N	2.701852	1.662527	-0.000007
H	3.265122	2.506079	-0.000037
C	4.703254	0.308164	-0.000013
H	5.321913	1.202006	-0.000033
C	1.330935	1.846417	0.000020
N	1.121305	-0.633483	0.000026
C	0.544213	0.604243	0.000015
C	3.085754	-1.997827	0.000034
H	2.441235	-2.871864	0.000050
C	-0.852088	0.722192	0.000007
H	-1.246422	1.733723	0.000005
C	3.313312	0.440307	0.000001
C	-5.232973	-1.578976	0.000004

H	-5.763584	-2.527111	0.000016
C	5.272108	-0.956497	-0.000001
H	6.353177	-1.058478	-0.000012
C	-1.707803	-0.381044	0.000007
H	-1.240640	-1.363708	0.000016
C	-5.950843	-0.376324	-0.000017
C	2.453055	-0.720627	0.000026
C	-3.879848	0.872011	-0.000027
H	-3.366186	1.828564	-0.000043
C	-5.261527	0.843108	-0.000033
H	-5.818022	1.776417	-0.000052
C	-3.126238	-0.337926	-0.000003
C	4.456379	-2.109981	0.000023
H	4.919986	-3.092203	0.000031
C	-3.851461	-1.563537	0.000011
H	-3.293419	-2.496708	0.000028
H	-7.037108	-0.388605	-0.000021

Supplementary Table 37. Cartesian coordinates of optimized SQ in the excited (n,π*) state calculated at PBE0-D3/6-31G(d,p) level with the SMD model of DCM.

	X	Y	Z
O	-0.822495	2.954590	-0.000122
N	-2.693701	1.658299	-0.000065
H	-3.256257	2.500865	0.000099
C	-4.730243	0.317673	0.000291
H	-5.331488	1.223313	0.000434
C	-1.346475	1.847923	-0.000428
N	-1.222314	-0.515429	-0.000261
C	-0.511261	0.597786	-0.000326
C	-3.151268	-2.005485	-0.000116
H	-2.523715	-2.890018	-0.000251
C	0.892381	0.697107	-0.000213
H	1.272079	1.713524	-0.000249
C	-3.347103	0.431201	0.000061
C	5.283136	-1.581947	0.000215
H	5.806301	-2.534723	0.000276
C	-5.328332	-0.942488	0.000314
H	-6.410452	-1.021262	0.000496
C	1.734210	-0.387638	-0.000085
H	1.290418	-1.381779	-0.000079

C	6.004848	-0.388173	0.000247
C	-2.537870	-0.740751	-0.000128
C	3.926718	0.852197	0.000062
H	3.415949	1.811053	0.000010
C	5.313885	0.825872	0.000169
H	5.865659	1.762453	0.000195
C	3.178056	-0.345377	0.000023
C	-4.536107	-2.091870	0.000108
H	-5.003910	-3.071914	0.000139
C	3.894863	-1.561051	0.000103
H	3.339851	-2.496520	0.000078
H	7.091002	-0.401691	0.000333

Supplementary Table 38. Cartesian coordinates of optimized SQ in the ground state calculated at PBE0-D3/6-31G(d,p) level with the SMD model of ACN.

	X	Y	Z
O	0.821930	2.943914	0.000167
N	2.697937	1.662214	-0.000109
H	3.258414	2.508308	0.000134
C	4.725368	0.326900	0.000255
H	5.337382	1.224786	0.000398
C	1.340420	1.831806	-0.000549
N	1.144814	-0.616985	-0.000229
C	0.572632	0.553697	-0.000215
C	3.124404	-1.975845	-0.000129
H	2.481268	-2.851157	-0.000243
C	-0.874530	0.687266	-0.000173
H	-1.242830	1.708515	-0.000334
C	3.331447	0.441790	0.000011
C	-5.260639	-1.574363	-0.000048
H	-5.783161	-2.526923	-0.000158
C	5.300048	-0.933922	0.000268
H	6.382324	-1.024743	0.000438
C	-1.712471	-0.369262	0.000023
H	-1.269194	-1.364947	0.000132
C	-5.979699	-0.381060	0.000103
C	2.513255	-0.709677	-0.000167
C	-3.909733	0.866325	0.000246
H	-3.396181	1.823507	0.000394
C	-5.297446	0.837827	0.000254

H	-5.854316	1.770820	0.000390
C	-3.169114	-0.330007	0.000075
C	4.502517	-2.088859	0.000073
H	4.968873	-3.069205	0.000103
C	-3.870183	-1.547689	-0.000054
H	-3.308880	-2.479080	-0.000172
H	-7.066046	-0.397367	0.000113

Supplementary Table 39. Cartesian coordinates of optimized SQ in the excited (π,π*) state calculated at PBE0-D3/6-31G(d,p) level with the SMD model of ACN.

	X	Y	Z
O	0.862575	2.981073	0.000049
N	2.700643	1.662674	0.000039
H	3.266449	2.505154	0.000066
C	4.703231	0.311567	0.000040
H	5.319954	1.206548	0.000070
C	1.330460	1.845221	0.000018
N	1.123243	-0.636426	-0.000030
C	0.543583	0.601703	-0.000006
C	3.089543	-1.998179	-0.000039
H	2.447484	-2.874172	-0.000068
C	-0.850903	0.719151	-0.000006
H	-1.245807	1.730577	0.000011
C	3.313033	0.441367	0.000023
C	-5.235484	-1.577696	-0.000057
H	-5.767260	-2.525194	-0.000094
C	5.273993	-0.952209	0.000019
H	6.355256	-1.052474	0.000033
C	-1.709492	-0.384041	-0.000030
H	-1.246008	-1.368578	-0.000063
C	-5.952133	-0.373987	-0.000003
C	2.454357	-0.721457	-0.000017
C	-3.879442	0.872538	0.000041
H	-3.365110	1.828699	0.000084
C	-5.261218	0.844912	0.000047
H	-5.816831	1.778760	0.000091
C	-3.126829	-0.338719	-0.000017
C	4.460188	-2.107727	-0.000021
H	4.925919	-3.088968	-0.000037
C	-3.853943	-1.564055	-0.000064

H	-3.297108	-2.497991	-0.000107
H	-7.038403	-0.385020	0.000002

Supplementary Table 40. Cartesian coordinates of optimized SQ in the excited (*n*,π*) state calculated at PBE0-D3/6-31G(d,p) level with the SMD model of ACN.

	X	Y	Z
O	0.827270	2.958181	0.000321
N	2.696427	1.658390	0.000054
H	3.263328	2.498686	0.000160
C	4.729389	0.313891	0.000010
H	5.332022	1.218456	0.000068
C	1.350222	1.850706	0.000040
N	1.219682	-0.513326	0.000001
C	0.512264	0.601802	0.000052
C	3.145390	-2.006955	-0.000094
H	2.515959	-2.890289	-0.000113
C	-0.891667	0.701767	0.000022
H	-1.272363	1.718059	-0.000001
C	3.346211	0.430033	-0.000030
C	-5.280259	-1.583698	-0.000006
H	-5.801911	-2.537352	0.000019
C	5.324488	-0.947677	-0.000053
H	6.406536	-1.028418	-0.000049
C	-1.732298	-0.384301	0.000010
H	-1.287609	-1.378267	0.000044
C	-6.004077	-0.390839	-0.000058
C	2.534945	-0.740716	-0.000081
C	-3.927158	0.852925	-0.000070
H	-3.417902	1.812660	-0.000096
C	-5.314700	0.824418	-0.000089
H	-5.867921	1.760193	-0.000130
C	-3.176563	-0.343804	-0.000017
C	4.530190	-2.096066	-0.000105
H	4.996149	-3.077067	-0.000139
C	-3.891668	-1.560823	0.000018
H	-3.335218	-2.495504	0.000061
H	-7.090252	-0.406028	-0.000075

Supplementary Table 41. Cartesian coordinates of optimized SQ in the ground state calculated at PBE0-D3/6-31G(d,p) level with the SMD model of MeOH.

	X	Y	Z
O	0.825512	2.944962	-0.000730
N	2.695242	1.660251	-0.000388
H	3.252979	2.509086	-0.000402
C	4.724295	0.328352	0.000217
H	5.335512	1.226802	0.000159
C	1.343035	1.825032	-0.000724
N	1.145018	-0.617297	-0.000127
C	0.571802	0.554054	-0.000410
C	3.124287	-1.976390	0.000349
H	2.482830	-2.852926	0.000404
C	-0.874764	0.687630	-0.000493
H	-1.247949	1.707004	-0.001457
C	3.330691	0.440120	-0.000029
C	-5.259936	-1.573796	-0.001347
H	-5.782447	-2.526495	-0.002476
C	5.299246	-0.931709	0.000505
H	6.381584	-1.022420	0.000682
C	-1.712593	-0.369035	0.000480
H	-1.270185	-1.364972	0.001226
C	-5.978949	-0.380656	-0.000245
C	2.512206	-0.710320	0.000053
C	-3.909447	0.866973	0.001630
H	-3.396740	1.824680	0.003031
C	-5.296976	0.838208	0.001279
H	-5.854045	1.771214	0.002274
C	-3.168874	-0.329274	0.000398
C	4.501849	-2.087116	0.000566
H	4.969305	-3.067063	0.000791
C	-3.869664	-1.547002	-0.000973
H	-3.308594	-2.478639	-0.001829
H	-7.065426	-0.397058	-0.000495

Supplementary Table 42. Cartesian coordinates of optimized SQ in the excited (π,π*) state calculated at PBE0-D3/6-31G(d,p) level with the SMD model of MeOH.

	X	Y	Z
O	0.858347	2.981810	-0.000354
N	2.695555	1.664204	0.000128
H	3.258519	2.508952	-0.000055
C	4.699299	0.313853	0.000085

H	5.315860	1.209034	0.000042
C	1.329820	1.839993	0.000367
N	1.123203	-0.637365	0.000076
C	0.544912	0.602186	0.000096
C	3.090607	-1.997614	0.000150
H	2.449714	-2.874596	0.000135
C	-0.851098	0.717719	-0.000008
H	-1.251202	1.727003	0.000086
C	3.310933	0.440879	0.000141
C	-5.233034	-1.578150	-0.000649
H	-5.765704	-2.525172	-0.001056
C	5.272481	-0.950207	0.000108
H	6.354101	-1.048066	0.000080
C	-1.708360	-0.386086	-0.000220
H	-1.245176	-1.370585	-0.000492
C	-5.948547	-0.373781	-0.000118
C	2.454621	-0.721261	0.000186
C	-3.876896	0.872817	0.000424
H	-3.363017	1.829202	0.000900
C	-5.258112	0.845412	0.000427
H	-5.814013	1.779115	0.000865
C	-3.125204	-0.339256	-0.000147
C	4.461377	-2.106049	0.000143
H	4.928070	-3.086950	0.000134
C	-3.851986	-1.564937	-0.000660
H	-3.295162	-2.498901	-0.001076
H	-7.034948	-0.384493	-0.000115

Supplementary Table 43. Cartesian coordinates of optimized SQ in the excited (n,π*) state calculated at PBE0-D3/6-31G(d,p) level with the SMD model of MeOH.

	X	Y	Z
O	0.830174	2.964616	0.000170
N	2.693531	1.656831	0.000037
H	3.256141	2.500595	0.000053
C	4.727378	0.311163	0.000073
H	5.330264	1.215709	0.000092
C	1.345822	1.849084	0.000072
N	1.222801	-0.505795	0.000084
C	0.511610	0.609839	0.000069
C	3.143533	-2.009490	0.000054

H	2.513951	-2.892674	0.000051
C	-0.893378	0.703993	0.000019
H	-1.275934	1.719773	-0.000058
C	3.344875	0.429929	0.000030
C	-5.276714	-1.586297	-0.000646
H	-5.797359	-2.540580	-0.001048
C	5.322354	-0.950356	0.000082
H	6.404476	-1.031142	0.000104
C	-1.730668	-0.382294	-0.000002
H	-1.285367	-1.376168	-0.000027
C	-6.001352	-0.394425	-0.000244
C	2.534552	-0.743603	0.000039
C	-3.926745	0.851968	0.000409
H	-3.419022	1.812579	0.000911
C	-5.314113	0.821794	0.000292
H	-5.868707	1.756839	0.000649
C	-3.175842	-0.343743	-0.000042
C	4.528446	-2.098202	0.000049
H	4.993999	-3.079488	0.000023
C	-3.888084	-1.561537	-0.000547
H	-3.330553	-2.495647	-0.000878
H	-7.087655	-0.410924	-0.000326

Supplementary Table 44. Cartesian coordinates of optimized MeOSQ in the ground state calculated at PBE0-D3/6-31G(d,p) level with the SMD model of *n*-hexane.

	X	Y	Z
O	1.653451	2.942937	-0.018473
N	3.538426	1.678946	-0.009584
H	4.076229	2.537065	-0.014750
O	-6.462391	-0.604138	-0.008512
C	5.580606	0.361253	-0.001104
H	6.187380	1.263066	-0.006152
C	2.175536	1.836190	-0.011195
N	2.007808	-0.615301	0.004272
C	1.421300	0.548386	-0.003157
C	4.001372	-1.950612	0.012149
H	3.362412	-2.828293	0.017191
C	-0.022941	0.671917	-0.003930
H	-0.395490	1.691179	-0.010816
C	4.187101	0.464947	-0.002101

C	-4.379024	-1.648986	-0.026802
H	-4.912829	-2.593700	-0.047647
C	6.168082	-0.893903	0.006359
H	7.250906	-0.974860	0.007094
C	-0.850550	-0.394140	0.000926
H	-0.384646	-1.379475	0.002252
C	-5.120363	-0.461468	-0.002917
C	3.377034	-0.692401	0.004668
C	-3.064851	0.799378	0.028178
H	-2.567758	1.764634	0.053444
C	-4.451507	0.769182	0.025733
H	-5.002850	1.702358	0.047256
C	-2.301883	-0.378307	0.002179
C	5.380448	-2.052995	0.012927
H	5.853925	-3.029666	0.018663
C	-2.998105	-1.601314	-0.023480
H	-2.432800	-2.529783	-0.042580
C	-7.257122	0.564372	0.011535
H	-8.293821	0.224153	-0.000374
H	-7.077451	1.192506	-0.870119
H	-7.086125	1.155917	0.919788

Supplementary Table 45. Cartesian coordinates of optimized MeOSQ in the excited (π,π*) state calculated at PBE0-D3/6-31G(d,p) level with the SMD model of *n*-hexane.

	X	Y	Z
O	1.687063	2.990424	-0.016261
N	3.538553	1.682765	-0.004702
H	4.085390	2.534298	-0.006506
O	-6.425825	-0.599490	-0.003054
C	5.552560	0.338080	0.005873
H	6.168624	1.234080	0.005471
C	2.160589	1.856833	-0.010335
N	1.978611	-0.619572	-0.002927
C	1.399366	0.607977	-0.007496
C	3.953175	-1.971165	0.006507
H	3.310046	-2.845936	0.006577
C	-0.012054	0.701320	-0.007384
H	-0.416724	1.709141	-0.010875
C	4.163030	0.463114	0.001267
C	-4.351940	-1.658045	-0.018560

H	-4.894614	-2.597955	-0.033116
C	6.132025	-0.924035	0.011011
H	7.213875	-1.016597	0.014827
C	-0.839023	-0.410969	-0.005446
H	-0.348272	-1.381792	-0.009832
C	-5.091682	-0.458760	0.000060
C	3.315439	-0.700272	0.001641
C	-3.036061	0.802060	0.020696
H	-2.535926	1.765143	0.039795
C	-4.416369	0.774483	0.020842
H	-4.967499	1.708155	0.037860
C	-2.263132	-0.390776	-0.000974
C	5.326319	-2.078505	0.011223
H	5.793508	-3.058967	0.015070
C	-2.978908	-1.620557	-0.018660
H	-2.415781	-2.549908	-0.033896
C	-7.233174	0.564624	0.012205
H	-8.265494	0.212946	0.003305
H	-7.056023	1.186465	-0.873622
H	-7.062137	1.158660	0.918043

Supplementary Table 46. Cartesian coordinates of optimized MeOSQ in the excited (n,π*) state calculated at PBE0-D3/6-31G(d,p) level with the SMD model of *n*-hexane.

	X	Y	Z
O	1.644639	2.944335	-0.001509
N	3.530200	1.674685	-0.000303
H	4.067469	2.531014	-0.000426
O	-6.493654	-0.599534	-0.000078
C	5.590622	0.367253	0.000576
H	6.179888	1.280898	0.000560
C	2.175771	1.842983	-0.000911
N	2.097464	-0.519567	-0.000279
C	1.362461	0.580855	-0.000706
C	4.050652	-1.978076	0.000496
H	3.438395	-2.872811	0.000437
C	-0.039842	0.662282	-0.000651
H	-0.426031	1.675824	-0.000902
C	4.207877	0.459090	0.000243
C	-4.410196	-1.655518	-0.001401
H	-4.949102	-2.597931	-0.002448

C	6.211432	-0.882999	0.000982
H	7.294269	-0.944358	0.001313
C	-0.874646	-0.427857	-0.000439
H	-0.419864	-1.416742	-0.000643
C	-5.146517	-0.464181	0.000011
C	3.414115	-0.724616	0.000214
C	-3.079443	0.782290	0.001384
H	-2.581522	1.747504	0.002722
C	-4.467043	0.759389	0.001448
H	-5.010760	1.697631	0.002700
C	-2.315555	-0.400438	-0.000168
C	5.436554	-2.042107	0.000937
H	5.918683	-3.015028	0.001236
C	-3.029974	-1.619127	-0.001474
H	-2.474479	-2.553926	-0.002632
C	-7.275205	0.575145	0.001184
H	-8.316218	0.247111	0.000738
H	-7.094250	1.186194	-0.892761
H	-7.094388	1.184181	0.896528

Supplementary Table 47. Cartesian coordinates of optimized MeOSQ in the ground state calculated at PBE0-D3/6-31G(d,p) level with the SMD model of dioxane.

	X	Y	Z
O	1.652372	2.942831	-0.021777
N	3.537536	1.679052	-0.011663
H	4.076266	2.536709	-0.018108
O	-6.462131	-0.603834	-0.010467
C	5.579617	0.361999	-0.001956
H	6.186142	1.264018	-0.008277
C	2.174901	1.835945	-0.013172
N	2.007439	-0.615542	0.005612
C	1.420880	0.548206	-0.003280
C	4.001299	-1.950281	0.014663
H	3.363089	-2.828605	0.020991
C	-0.023256	0.670909	-0.004047
H	-0.397161	1.689645	-0.012039
C	4.186160	0.465188	-0.002749
C	-4.378659	-1.649351	-0.031882
H	-4.911742	-2.594447	-0.056802
C	6.167406	-0.892813	0.006957

H	7.250325	-0.973420	0.007524
C	-0.850469	-0.395379	0.001501
H	-0.385074	-1.380983	0.002913
C	-5.120021	-0.461993	-0.003563
C	3.376613	-0.692329	0.005732
C	-3.064294	0.798300	0.033761
H	-2.567479	1.763642	0.063897
C	-4.450825	0.768342	0.030665
H	-5.001414	1.701900	0.056270
C	-2.301537	-0.379294	0.002900
C	5.380253	-2.052120	0.015196
H	5.854094	-3.028686	0.022041
C	-2.997831	-1.601965	-0.027742
H	-2.432930	-2.530669	-0.050434
C	-7.254205	0.567009	0.013029
H	-8.291876	0.229698	-0.001334
H	-7.072167	1.197820	-0.866191
H	-7.083046	1.154593	0.923778

Supplementary Table 48. Cartesian coordinates of optimized MeOSQ in the excited (π,π*) state calculated at PBE0-D3/6-31G(d,p) level with the SMD model of dioxane.

	X	Y	Z
O	1.685372	2.989884	-0.018812
N	3.537192	1.682922	-0.005704
H	4.084816	2.534055	-0.007964
O	-6.425507	-0.599036	-0.003974
C	5.551510	0.339500	0.006317
H	6.167013	1.235940	0.005627
C	2.159607	1.856376	-0.011935
N	1.978687	-0.620520	-0.003034
C	1.398510	0.607137	-0.008352
C	3.954037	-1.970739	0.007641
H	3.312002	-2.846423	0.007963
C	-0.012065	0.699932	-0.008092
H	-0.417847	1.707259	-0.012190
C	4.162113	0.463507	0.001226
C	-4.351782	-1.658560	-0.021524
H	-4.893861	-2.598833	-0.038644
C	6.131898	-0.922090	0.012300
H	7.213881	-1.013868	0.016514

C	-0.839531	-0.412596	-0.005812
H	-0.349732	-1.383896	-0.010964
C	-5.091453	-0.459246	0.000025
C	3.315266	-0.700304	0.001969
C	-3.035632	0.801139	0.024555
H	-2.535827	1.764386	0.047008
C	-4.415698	0.773797	0.024484
H	-4.966111	1.707876	0.044365
C	-2.262860	-0.391830	-0.000662
C	5.327077	-2.077043	0.012841
H	5.794962	-3.057249	0.017323
C	-2.978976	-1.621438	-0.021372
H	-2.416327	-2.551103	-0.039069
C	-7.230294	0.567487	0.013261
H	-8.263575	0.218579	0.002523
H	-7.050926	1.190773	-0.871049
H	-7.058900	1.158740	0.920790

Supplementary Table 49. Cartesian coordinates of optimized MeOSQ in the excited (n,π*) state calculated at PBE0-D3/6-31G(d,p) level with the SMD model of dioxane.

	X	Y	Z
O	1.644689	2.945332	-0.002700
N	3.529846	1.674836	-0.000322
H	4.068309	2.530594	-0.000174
O	-6.492706	-0.599926	0.000526
C	5.589268	0.366616	0.001718
H	6.178936	1.280046	0.002109
C	2.175662	1.843660	-0.002072
N	2.096249	-0.518928	-0.001123
C	1.361929	0.581793	-0.001822
C	4.048592	-1.978113	0.000358
H	3.436010	-2.872737	-0.000190
C	-0.040313	0.662805	-0.001498
H	-0.427539	1.675953	-0.001777
C	4.206665	0.458963	0.000718
C	-4.408877	-1.656377	-0.001427
H	-4.946970	-2.599326	-0.002515
C	6.209397	-0.883775	0.002280
H	7.292295	-0.945508	0.003215
C	-0.874134	-0.427843	-0.001002

H	-0.418911	-1.416608	-0.001315
C	-5.145571	-0.465304	0.000345
C	3.412749	-0.724491	0.000066
C	-3.078955	0.781620	0.001508
H	-2.581516	1.747190	0.002943
C	-4.466439	0.758364	0.001849
H	-5.010147	1.696698	0.003386
C	-2.314878	-0.400842	-0.000427
C	5.434304	-2.042605	0.001586
H	5.916159	-3.015747	0.002002
C	-3.028763	-1.619640	-0.001803
H	-2.473133	-2.554435	-0.003247
C	-7.270948	0.577301	0.002170
H	-8.313094	0.252755	0.001901
H	-7.088652	1.188037	-0.891730
H	-7.088394	1.185707	0.897603

Supplementary Table 50. Cartesian coordinates of optimized MeOSQ in the ground state calculated at PBE0-D3/6-31G(d,p) level with the SMD model of THF.

	X	Y	Z
O	1.644798	2.937689	-0.018190
N	3.535615	1.680370	-0.009513
H	4.079691	2.536421	-0.014513
O	-6.463798	-0.600785	-0.008493
C	5.580573	0.369949	-0.001036
H	6.182041	1.275177	-0.006036
C	2.174732	1.832419	-0.011104
N	2.011171	-0.619532	0.004468
C	1.422228	0.543789	-0.002890
C	4.009454	-1.950248	0.012039
H	3.376572	-2.832889	0.017006
C	-0.022861	0.663670	-0.003442
H	-0.400155	1.681363	-0.009255
C	4.185911	0.467754	-0.002031
C	-4.382573	-1.650909	-0.026299
H	-4.915159	-2.596765	-0.046478
C	6.172363	-0.883238	0.006284
H	7.255687	-0.960210	0.006987
C	-0.852380	-0.401997	0.000500
H	-0.394251	-1.391129	0.000999

C	-5.123228	-0.461555	-0.003105
C	3.380953	-0.693134	0.004703
C	-3.065234	0.797509	0.026951
H	-2.567842	1.762995	0.051273
C	-4.452145	0.769223	0.024667
H	-5.002642	1.703076	0.045553
C	-2.303812	-0.382385	0.001778
C	5.389113	-2.046692	0.012738
H	5.866839	-3.021554	0.018343
C	-3.001085	-1.605494	-0.023082
H	-2.436756	-2.534789	-0.041490
C	-7.257028	0.574182	0.012345
H	-8.294513	0.236641	0.001440
H	-7.073964	1.200007	-0.869479
H	-7.080929	1.163498	0.920321

Supplementary Table 51. Cartesian coordinates of optimized MeOSQ in the excited (π,π*) state calculated at PBE0-D3/6-31G(d,p) level with the SMD model of THF.

	X	Y	Z
O	1.666228	2.976427	-0.020529
N	3.527686	1.685285	-0.005553
H	4.075858	2.537832	-0.006553
O	-6.428073	-0.589354	-0.001296
C	5.552361	0.359953	0.009475
H	6.157401	1.263338	0.009866
C	2.152429	1.847434	-0.012908
N	1.989026	-0.634313	-0.005065
C	1.394893	0.591730	-0.010304
C	3.977055	-1.967690	0.007750
H	3.346249	-2.851989	0.006984
C	-0.009740	0.681681	-0.010684
H	-0.418128	1.687999	-0.013601
C	4.162077	0.470134	0.002118
C	-4.361502	-1.660826	-0.022705
H	-4.906620	-2.599620	-0.039612
C	6.145559	-0.896573	0.016169
H	7.228484	-0.978109	0.022114
C	-0.847816	-0.430341	-0.009978
H	-0.369985	-1.407578	-0.016913
C	-5.096669	-0.456557	0.000608

C	3.324444	-0.701867	0.001392
C	-3.035861	0.797528	0.022670
H	-2.533352	1.759505	0.044594
C	-4.415242	0.775529	0.024607
H	-4.963483	1.710918	0.045585
C	-2.266754	-0.401027	-0.003780
C	5.351219	-2.060217	0.015211
H	5.828781	-3.035921	0.020334
C	-2.989194	-1.630138	-0.024263
H	-2.430099	-2.562060	-0.042977
C	-7.231983	0.583108	0.019107
H	-8.265495	0.235553	0.011046
H	-7.050318	1.204389	-0.865308
H	-7.053203	1.171178	0.926462

Supplementary Table 52. Cartesian coordinates of optimized MeOSQ in the excited (n,π*) state calculated at PBE0-D3/6-31G(d,p) level with the SMD model of THF.

	X	Y	Z
O	1.649507	2.949497	-0.004031
N	3.535265	1.676157	-0.002355
H	4.083944	2.527496	-0.003436
O	-6.491500	-0.604496	-0.002040
C	5.588833	0.360878	-0.000442
H	6.179289	1.273679	-0.001801
C	2.183710	1.847575	-0.002399
N	2.092071	-0.516738	0.001286
C	1.365640	0.589116	-0.000638
C	4.040179	-1.981403	0.003336
H	3.424400	-2.874219	0.004796
C	-0.038591	0.670665	-0.001292
H	-0.429494	1.682857	-0.002970
C	4.205200	0.457347	-0.000674
C	-4.405221	-1.657023	-0.006287
H	-4.939064	-2.602809	-0.010984
C	6.204272	-0.891938	0.001460
H	7.287307	-0.956586	0.001498
C	-0.870216	-0.422049	-0.000482
H	-0.415160	-1.411274	-0.000635
C	-5.145712	-0.467191	-0.000786
C	3.409320	-0.725006	0.001253

C	-3.080099	0.784832	0.006197
H	-2.585370	1.752121	0.012272
C	-4.468502	0.758599	0.005716
H	-5.014689	1.695601	0.010772
C	-2.313037	-0.396544	0.000009
C	5.426427	-2.050205	0.003307
H	5.905933	-3.024702	0.004775
C	-3.024035	-1.617535	-0.005780
H	-2.466058	-2.551103	-0.010295
C	-7.275570	0.573768	0.003646
H	-8.316249	0.244976	0.001417
H	-7.092444	1.186980	-0.887646
H	-7.093340	1.177712	0.901416

Supplementary Table 53. Cartesian coordinates of optimized MeOSQ in the ground state calculated at PBE0-D3/6-31G(d,p) level with the SMD model of DCM.

	X	Y	Z
O	1.654366	2.943313	-0.005198
N	3.540384	1.678643	-0.002737
H	4.088652	2.532129	-0.004199
O	-6.462275	-0.607060	-0.002276
C	5.581277	0.362164	-0.000255
H	6.185126	1.265769	-0.001688
C	2.180894	1.835223	-0.003160
N	2.008782	-0.615370	0.001294
C	1.423499	0.550094	-0.000860
C	4.002518	-1.953631	0.003511
H	3.367174	-2.834522	0.004935
C	-0.021581	0.673471	-0.001091
H	-0.398110	1.691523	-0.002783
C	4.186797	0.463861	-0.000563
C	-4.378213	-1.649987	-0.007611
H	-4.908635	-2.597326	-0.013384
C	6.169037	-0.892949	0.001873
H	7.252092	-0.973424	0.002095
C	-0.851866	-0.391840	-0.000016
H	-0.393648	-1.380911	0.000098
C	-5.122028	-0.462319	-0.000869
C	3.378107	-0.694215	0.001383
C	-3.068164	0.803669	0.007670

H	-2.573372	1.770824	0.014713
C	-4.455190	0.771098	0.007058
H	-5.009527	1.702921	0.013072
C	-2.303376	-0.374582	0.000371
C	5.381910	-2.054169	0.003726
H	5.856666	-3.030492	0.005344
C	-2.996843	-1.600321	-0.006777
H	-2.429409	-2.527886	-0.012112
C	-7.263952	0.562704	0.003681
H	-8.298701	0.216815	0.000724
H	-7.087224	1.176127	-0.887784
H	-7.088935	1.165870	0.902449

Supplementary Table 54. Cartesian coordinates of optimized MeOSQ in the excited (π,π*) state calculated at PBE0-D3/6-31G(d,p) level with the SMD model of DCM.

	X	Y	Z
O	1.673776	2.980559	-0.007781
N	3.531166	1.683887	-0.001652
H	4.082145	2.534684	-0.001693
O	-6.426164	-0.594362	0.000932
C	5.552914	0.354000	0.004618
H	6.159631	1.256219	0.005196
C	2.157347	1.849234	-0.004748
N	1.987473	-0.631560	-0.002597
C	1.396035	0.596387	-0.004524
C	3.972313	-1.970370	0.002846
H	3.339787	-2.853468	0.002111
C	-0.008311	0.689291	-0.005140
H	-0.416247	1.695838	-0.006084
C	4.162968	0.466912	0.001263
C	-4.357852	-1.659640	-0.008604
H	-4.901383	-2.599515	-0.014396
C	6.143458	-0.904075	0.007183
H	7.226205	-0.988031	0.009895
C	-0.847988	-0.422257	-0.005241
H	-0.370886	-1.399842	-0.007928
C	-5.095700	-0.456467	0.000437
C	3.322642	-0.702796	0.000411
C	-3.038469	0.803562	0.007103
H	-2.537955	1.766869	0.014760

C	-4.417761	0.778098	0.008543
H	-4.969626	1.711590	0.016323
C	-2.266455	-0.394045	-0.002765
C	5.346433	-2.065987	0.006265
H	5.821798	-3.042779	0.008231
C	-2.985895	-1.625617	-0.009985
H	-2.424116	-2.556067	-0.017103
C	-7.239636	0.571951	0.009237
H	-8.269882	0.214898	0.007549
H	-7.064132	1.183285	-0.882925
H	-7.062969	1.171374	0.909212

Supplementary Table 55. Cartesian coordinates of optimized MeOSQ in the excited (n,π*) state calculated at PBE0-D3/6-31G(d,p) level with the SMD model of DCM.

	X	Y	Z
O	1.660801	2.957709	-0.002917
N	3.540922	1.674516	-0.001776
H	4.094867	2.522540	-0.002559
O	-6.489018	-0.611937	-0.001497
C	5.588758	0.350345	-0.000444
H	6.182656	1.260886	-0.001462
C	2.190076	1.852707	-0.001759
N	2.088923	-0.510398	0.000970
C	1.366980	0.598765	-0.000479
C	4.029866	-1.985647	0.002399
H	3.410248	-2.875792	0.003493
C	-0.037518	0.683078	-0.001027
H	-0.427692	1.695707	-0.002369
C	4.205370	0.452945	-0.000571
C	-4.399339	-1.656329	-0.005344
H	-4.930580	-2.603661	-0.009373
C	6.198759	-0.905192	0.000935
H	7.281520	-0.974416	0.000926
C	-0.868566	-0.410093	-0.000400
H	-0.412366	-1.398867	-0.000563
C	-5.143423	-0.468708	-0.000555
C	3.404858	-0.726406	0.000883
C	-3.082542	0.790797	0.005365
H	-2.590557	1.759518	0.010577
C	-4.471088	0.759761	0.004986

H	-5.021508	1.694313	0.009340
C	-2.311709	-0.388247	0.000016
C	5.416019	-2.060310	0.002322
H	5.891354	-3.036843	0.003377
C	-3.018177	-1.612012	-0.004973
H	-2.456644	-2.543445	-0.008889
C	-7.281595	0.561213	0.003001
H	-8.319599	0.224223	0.001079
H	-7.102177	1.174140	-0.888960
H	-7.103085	1.166686	0.900216

Supplementary Table 56. Cartesian coordinates of optimized MeOSQ in the ground state calculated at PBE0-D3/6-31G(d,p) level with the SMD model of ACN.

	X	Y	Z
O	1.652870	2.942803	0.000184
N	3.539906	1.678979	0.000136
H	4.091156	2.531047	0.000239
O	-6.462399	-0.606412	0.000149
C	5.580838	0.363811	0.000105
H	6.183551	1.268030	0.000221
C	2.180998	1.835066	0.000101
N	2.008994	-0.615896	-0.000170
C	1.423682	0.549793	-0.000047
C	4.003414	-1.953833	-0.000205
H	3.369504	-2.835919	-0.000320
C	-0.021747	0.672175	-0.000070
H	-0.399680	1.689837	-0.000113
C	4.186027	0.464435	0.000046
C	-4.378761	-1.651140	0.000316
H	-4.908187	-2.599162	0.000525
C	6.169216	-0.891010	0.000013
H	7.252362	-0.970776	0.000061
C	-0.852072	-0.393505	0.000012
H	-0.395709	-1.383575	0.000106
C	-5.122771	-0.463076	0.000076
C	3.378433	-0.694400	-0.000112
C	-3.068327	0.802855	-0.000241
H	-2.573780	1.770196	-0.000478
C	-4.455404	0.770532	-0.000205
H	-5.009727	1.702351	-0.000400

C	-2.303646	-0.375858	0.000011
C	5.382933	-2.053265	-0.000138
H	5.858606	-3.029194	-0.000204
C	-2.997176	-1.601798	0.000282
H	-2.429928	-2.529531	0.000469
C	-7.262150	0.566424	-0.000212
H	-8.297582	0.222887	-0.000208
H	-7.083711	1.173713	-0.895238
H	-7.083852	1.174164	0.894537

Supplementary Table 57. Cartesian coordinates of optimized MeOSQ in the excited (π,π*) state calculated at PBE0-D3/6-31G(d,p) level with the SMD model of ACN.

	X	Y	Z
O	-1.668900	2.977315	0.000076
N	-3.528440	1.684355	0.000025
H	-4.080944	2.534715	0.000062
O	6.426781	-0.591757	0.000026
C	-5.552603	0.359669	-0.000002
H	-6.156387	1.263715	0.000032
C	-2.155401	1.847356	0.000009
N	-1.989790	-0.635508	-0.000052
C	-1.394384	0.592571	-0.000014
C	-3.978181	-1.969662	-0.000091
H	-3.349282	-2.855534	-0.000123
C	0.007663	0.684738	0.000002
H	0.416702	1.690908	0.000026
C	-4.162384	0.468663	-0.000008
C	4.360292	-1.661103	-0.000034
H	4.903866	-2.601027	-0.000071
C	-6.146662	-0.896875	-0.000042
H	-7.229650	-0.977907	-0.000037
C	0.850485	-0.427316	-0.000018
H	0.376782	-1.406721	-0.000055
C	5.097288	-0.456627	0.000025
C	-3.324444	-0.703429	-0.000053
C	3.038780	0.802012	0.000068
H	2.537904	1.765139	0.000115
C	4.417725	0.777773	0.000077
H	4.969126	1.711506	0.000127
C	2.267351	-0.397042	0.000003

C	-5.352579	-2.061315	-0.000086
H	-5.830717	-3.036797	-0.000115
C	2.988562	-1.628621	-0.000044
H	2.427701	-2.559719	-0.000091
C	7.237541	0.578460	0.000079
H	8.268676	0.224375	0.000075
H	7.058232	1.182687	0.895988
H	7.058247	1.182755	-0.895786

Supplementary Table 58. Cartesian coordinates of optimized MeOSQ in the excited (n,π^*) state calculated at PBE0-D3/6-31G(d,p) level with the SMD model of ACN.

	X	Y	Z
O	1.665445	2.961272	-0.001509
N	3.543719	1.674588	-0.000908
H	4.103006	2.519721	-0.001451
O	-6.487744	-0.614881	-0.000988
C	5.587704	0.345926	-0.000602
H	6.182957	1.255452	-0.001298
C	2.194370	1.855601	-0.000744
N	2.085446	-0.508677	0.000889
C	1.368117	0.603327	0.000069
C	4.023484	-1.987321	0.001313
H	3.401819	-2.876166	0.002031
C	-0.036929	0.688787	-0.000222
H	-0.428543	1.701055	-0.001063
C	4.204104	0.451308	-0.000368
C	-4.396364	-1.657022	-0.002349
H	-4.924913	-2.605998	-0.004224
C	6.194548	-0.911035	0.000046
H	7.277262	-0.982422	-0.000186
C	-0.866582	-0.405769	0.000299
H	-0.409676	-1.394426	0.000499
C	-5.142843	-0.470465	-0.000343
C	3.401563	-0.726512	0.000607
C	-3.083169	0.792336	0.002672
H	-2.593050	1.762076	0.005141
C	-4.471956	0.759180	0.002266
H	-5.023823	1.692876	0.004167
C	-2.310342	-0.385669	0.000350
C	5.409634	-2.065073	0.000983

H	5.883096	-3.042571	0.001463
C	-3.014899	-1.610683	-0.001974
H	-2.451910	-2.541303	-0.003632
C	-7.280761	0.559632	0.000611
H	-8.318765	0.222957	-0.000539
H	-7.100144	1.170021	-0.892569
H	-7.101144	1.166933	0.896093

Supplementary Table 59. Cartesian coordinates of optimized MeOSQ in the ground state calculated at PBE0-D3/6-31G(d,p) level with the SMD model of MeOH.

	X	Y	Z
O	1.655158	2.943511	0.000239
N	3.536362	1.677189	0.000250
H	4.084652	2.532083	0.000518
O	-6.463648	-0.605245	0.000097
C	5.579489	0.366594	0.000329
H	6.180943	1.271670	0.000624
C	2.182863	1.828007	0.000125
N	2.009510	-0.616578	-0.000523
C	1.422709	0.549472	-0.000240
C	4.004175	-1.953961	-0.000472
H	3.372408	-2.837562	-0.000772
C	-0.022374	0.670731	-0.000445
H	-0.406236	1.686069	-0.001097
C	4.184958	0.463316	0.000105
C	-4.378434	-1.651010	0.000482
H	-4.909312	-2.598443	0.000716
C	6.168680	-0.887148	0.000166
H	7.251924	-0.966266	0.000346
C	-0.851865	-0.395448	0.000273
H	-0.396367	-1.385733	0.000971
C	-5.119469	-0.462378	0.000112
C	3.377708	-0.694893	-0.000298
C	-3.066919	0.802413	-0.000120
H	-2.572560	1.769866	-0.000296
C	-4.453902	0.770910	-0.000175
H	-5.007748	1.703022	-0.000415
C	-2.303318	-0.376873	0.000219
C	5.383133	-2.050332	-0.000226
H	5.860466	-3.025566	-0.000342

C	-2.996737	-1.602540	0.000555
H	-2.430329	-2.530819	0.000849
C	-7.263336	0.569317	-0.000424
H	-8.298557	0.224905	-0.000472
H	-7.083171	1.174365	-0.896395
H	-7.083457	1.174965	0.895199

Supplementary Table 60. Cartesian coordinates of optimized MeOSQ in the excited (π,π*) state calculated at PBE0-D3/6-31G(d,p) level with the SMD model of MeOH.

	X	Y	Z
O	-1.663454	2.976516	0.000368
N	-3.521801	1.685036	0.000188
H	-4.070764	2.538173	0.000182
O	6.424817	-0.588677	-0.000026
C	-5.549175	0.364886	-0.000227
H	-6.151131	1.270204	-0.000212
C	-2.153770	1.839286	0.000412
N	-1.990248	-0.637564	0.000055
C	-1.394783	0.590605	0.000232
C	-3.981466	-1.967806	-0.000264
H	-3.355276	-2.855661	-0.000288
C	0.009448	0.680050	0.000231
H	0.424707	1.683464	0.000360
C	-4.160181	0.468677	-0.000035
C	4.360029	-1.662185	0.000086
H	4.907035	-2.600267	0.000146
C	-6.146842	-0.890686	-0.000425
H	-7.230158	-0.968537	-0.000572
C	0.850263	-0.432252	0.000114
H	0.377338	-1.411830	0.000088
C	5.093710	-0.455860	-0.000016
C	-3.325631	-0.703264	-0.000056
C	3.036219	0.800534	-0.000077
H	2.534892	1.763355	-0.000178
C	4.414173	0.778439	-0.000109
H	4.964527	1.712739	-0.000214
C	2.266772	-0.400335	0.000053
C	-5.356233	-2.056207	-0.000440
H	-5.836516	-3.030745	-0.000599
C	2.988783	-1.631657	0.000113

H	2.428993	-2.563383	0.000196
C	7.235985	0.582943	-0.000095
H	8.266530	0.227226	-0.000055
H	7.054713	1.184671	0.896837
H	7.054740	1.184547	-0.897115

Supplementary Table 61. Cartesian coordinates of optimized MeOSQ in the excited (n,π*) state calculated at PBE0-D3/6-31G(d,p) level with the SMD model of MeOH.

	X	Y	Z
O	1.668718	2.968130	0.000308
N	3.540869	1.672834	0.000315
H	4.096421	2.520997	0.000057
O	-6.486963	-0.617487	0.000019
C	5.585342	0.342617	-0.000298
H	6.181078	1.251956	-0.000303
C	2.190361	1.854202	0.000300
N	2.087866	-0.501046	0.000377
C	1.367109	0.611202	0.000326
C	4.020746	-1.990038	-0.000204
H	3.398677	-2.878558	-0.000177
C	-0.038805	0.691478	0.000264
H	-0.432473	1.703067	0.000246
C	4.202388	0.450666	0.000011
C	-4.392893	-1.658352	0.000549
H	-4.921518	-2.607485	0.000929
C	6.191708	-0.914382	-0.000548
H	7.274485	-0.985912	-0.000772
C	-0.865035	-0.403570	0.000258
H	-0.407424	-1.392046	0.000404
C	-5.137531	-0.472055	0.000000
C	3.400824	-0.729465	0.000058
C	-3.081938	0.792379	-0.000471
H	-2.592920	1.762665	-0.000956
C	-4.470413	0.758438	-0.000522
H	-5.023126	1.691621	-0.000996
C	-2.309359	-0.385271	0.000127
C	5.406970	-2.067776	-0.000492
H	5.879803	-3.045650	-0.000675
C	-3.011309	-1.610923	0.000611
H	-2.447756	-2.541203	0.001051

C	-7.281609	0.558193	-0.000622
H	-8.318893	0.219171	-0.000547
H	-7.100360	1.164388	-0.896175
H	-7.100517	1.165259	0.894372

Supplementary Table 62. Cartesian coordinates of optimized DPASQ in the ground state calculated at PBE0-D3/6-31G(d,p) level with the SMD model of *n*-hexane.

	X	Y	Z
O	-4.480672	-2.706441	-1.183699
N	3.861310	0.079758	0.034314
N	-6.302737	-1.465606	-0.642581
H	-6.882208	-2.227126	-0.973829
C	-0.372891	0.163218	0.061160
C	-1.819691	0.242398	0.097325
H	-2.237632	1.164349	0.501547
C	-2.697586	-0.699047	-0.311453
H	-2.373571	-1.649794	-0.722488
C	-6.591042	1.879076	0.812575
H	-5.910656	2.655035	1.149600
N	-4.663908	0.568826	0.240827
C	1.715284	-0.965776	-0.469676
H	2.234843	-1.821403	-0.888370
C	4.605047	1.272938	-0.132418
C	-8.279274	-0.169801	-0.077181
H	-8.928684	-0.969334	-0.424092
C	6.358965	-2.625444	-0.425454
H	7.220499	-2.837748	-1.052232
C	5.944938	-3.550566	0.530326
H	6.483368	-4.484733	0.658419
C	0.384217	1.235068	0.563355
H	-0.132843	2.097568	0.976852
C	1.768232	1.214705	0.565362
H	2.323893	2.052243	0.973934
C	0.334562	-0.938343	-0.455740
H	-0.206946	-1.782627	-0.871821
C	2.460391	0.110645	0.045599
C	5.667352	-1.432504	-0.599639
H	5.983409	-0.716689	-1.352086
C	4.233510	2.210647	-1.102718
H	3.367100	2.016555	-1.727382

C	6.092764	3.619675	-0.471805
H	6.669645	4.530108	-0.603453
C	-4.948784	-1.669792	-0.732446
C	4.555381	-1.143004	0.199034
C	-6.892327	-0.325382	-0.145983
C	4.969712	3.378942	-1.260460
H	4.670133	4.098489	-2.017176
C	6.469348	2.680843	0.485961
H	7.340020	2.858679	1.110831
C	-6.027473	0.699295	0.298979
C	5.728959	1.517974	0.664120
H	6.016172	0.791212	1.417653
C	-8.805974	1.005649	0.434332
H	-9.883659	1.127257	0.487918
C	4.146905	-2.066921	1.167772
H	3.290299	-1.839059	1.794547
C	-4.133622	-0.523176	-0.234419
C	-7.963601	2.033008	0.880426
H	-8.389850	2.948286	1.278813
C	4.834051	-3.265404	1.321151
H	4.505277	-3.974116	2.075962

Supplementary Table 63. Cartesian coordinates of optimized DPASQ in the excited (π,π*) state calculated at PBE0-D3/6-31G(d,p) level with the SMD model of *n*-hexane.

	X	Y	Z
O	-4.408485	-2.448812	-1.663520
N	3.888760	0.070570	0.055628
N	-6.234247	-1.332190	-0.914979
H	-6.797897	-2.027458	-1.385572
C	-0.354023	0.192391	0.126184
C	-1.786668	0.277987	0.182749
H	-2.212510	1.104991	0.746522
C	-2.663733	-0.594327	-0.410398
H	-2.311966	-1.444282	-0.988968
C	-6.587601	1.691076	1.133392
H	-5.927573	2.403779	1.618937
N	-4.634095	0.537922	0.356356
C	1.725295	-0.862475	-0.601918
H	2.240395	-1.638553	-1.160289
C	4.605412	1.248747	-0.187370

C	-8.227716	-0.182538	-0.142530
H	-8.859842	-0.914121	-0.640632
C	6.360228	-2.669628	-0.196352
H	7.268747	-2.904613	-0.742423
C	5.821274	-3.595903	0.697343
H	6.316441	-4.548114	0.859789
C	0.429927	1.161595	0.801844
H	-0.076702	1.944340	1.359632
C	1.808498	1.128163	0.782906
H	2.385771	1.871513	1.324612
C	0.347483	-0.822696	-0.576787
H	-0.206824	-1.580897	-1.119637
C	2.475408	0.111849	0.077629
C	5.731818	-1.453866	-0.413972
H	6.132902	-0.746703	-1.132303
C	4.083370	2.216627	-1.064234
H	3.144395	2.025186	-1.572444
C	6.002818	3.622878	-0.651577
H	6.545323	4.545750	-0.831228
C	-4.866422	-1.501862	-1.025038
C	4.545073	-1.147047	0.276083
C	-6.842579	-0.307352	-0.222821
C	4.784300	3.390631	-1.290000
H	4.380825	4.126611	-1.978601
C	6.518371	2.665906	0.223741
H	7.456686	2.848081	0.738738
C	-5.979481	0.633026	0.416931
C	5.830310	1.487108	0.462276
H	6.215074	0.757112	1.166615
C	-8.788946	0.871965	0.571861
H	-9.869165	0.964815	0.631864
C	3.998992	-2.083816	1.171584
H	3.088967	-1.840550	1.709440
C	-4.081448	-0.476196	-0.329622
C	-7.963019	1.807913	1.209126
H	-8.406471	2.628950	1.765417
C	4.639725	-3.295392	1.375887
H	4.218378	-4.008043	2.078144

Supplementary Table 64. Cartesian coordinates of optimized DPASQ in the excited (n,π*) state

calculated at PBE0-D3/6-31G(d,p) level with the SMD model of *n*-hexane.

	X	Y	Z
O	-4.460694	-2.606855	-1.385697
N	3.891574	0.078908	0.044070
N	-6.288227	-1.412316	-0.751451
H	-6.866108	-2.147026	-1.136661
C	-0.357199	0.185426	0.088010
C	-1.791328	0.270119	0.133671
H	-2.199487	1.160101	0.609359
C	-2.677184	-0.659324	-0.359376
H	-2.338783	-1.571056	-0.839478
C	-6.642827	1.837295	0.958735
H	-5.990616	2.604285	1.360964
N	-4.757281	0.472080	0.236291
C	1.731597	-0.912927	-0.528487
H	2.247284	-1.740353	-1.006074
C	4.629568	1.266186	-0.156716
C	-8.287408	-0.176068	-0.097106
H	-8.917754	-0.960363	-0.508399
C	6.372289	-2.657049	-0.323146
H	7.252905	-2.880890	-0.918875
C	5.910071	-3.578280	0.614275
H	6.429405	-4.520582	0.760485
C	0.416879	1.215600	0.664032
H	-0.091447	2.051168	1.139364
C	1.799248	1.183478	0.660645
H	2.363437	1.986390	1.124832
C	0.350770	-0.882185	-0.508204
H	-0.192176	-1.695013	-0.981077
C	2.484004	0.116995	0.060127
C	5.705695	-1.453907	-0.521294
H	6.060697	-0.744400	-1.262172
C	4.206161	2.213884	-1.097192
H	3.307455	2.024543	-1.675832
C	6.093798	3.623820	-0.555398
H	6.660713	4.536933	-0.709828
C	-4.944040	-1.614295	-0.861635
C	4.568305	-1.145761	0.235881
C	-6.909940	-0.310949	-0.170447
C	4.930727	3.384850	-1.284328

H	4.588523	4.109406	-2.018056
C	6.520865	2.677819	0.373890
H	7.421895	2.853040	0.955205
C	-6.063971	0.704042	0.361693
C	5.793837	1.511856	0.582151
H	6.122465	0.783209	1.316599
C	-8.850641	0.953430	0.498249
H	-9.929559	1.050329	0.551333
C	4.110028	-2.068638	1.184616
H	3.232269	-1.831168	1.777478
C	-4.072147	-0.528327	-0.290910
C	-8.024170	1.948134	1.019903
H	-8.462128	2.827031	1.483318
C	4.773577	-3.276833	1.361576
H	4.404712	-3.981812	2.101429

Supplementary Table 65. Cartesian coordinates of optimized DPASQ in the ground state calculated at PBE0-D3/6-31G(d,p) level with the SMD model of dioxane.

	X	Y	Z
O	-4.479296	-2.705890	-1.184692
N	3.860802	0.079807	0.034341
N	-6.301490	-1.465308	-0.643133
H	-6.881956	-2.226135	-0.974538
C	-0.372682	0.164160	0.061175
C	-1.819226	0.243590	0.097519
H	-2.236597	1.165751	0.501915
C	-2.696812	-0.698029	-0.311322
H	-2.371554	-1.648376	-0.722222
C	-6.590462	1.878653	0.813259
H	-5.910771	2.655076	1.150891
N	-4.663062	0.569013	0.241191
C	1.714998	-0.965004	-0.470085
H	2.234083	-1.820551	-0.889777
C	4.604714	1.272579	-0.132339
C	-8.277900	-0.170236	-0.077569
H	-8.927061	-0.969831	-0.424903
C	6.359165	-2.624537	-0.422593
H	7.222463	-2.836011	-1.047343
C	5.942378	-3.551027	0.530506
H	6.480370	-4.485517	0.658679

C	0.384480	1.235644	0.563796
H	-0.132244	2.098152	0.977845
C	1.768375	1.215092	0.565669
H	2.323876	2.052410	0.975163
C	0.334401	-0.937269	-0.456272
H	-0.206894	-1.781418	-0.873074
C	2.460145	0.111073	0.045579
C	5.668353	-1.431270	-0.596849
H	5.986714	-0.714731	-1.347767
C	4.230665	2.212336	-1.099532
H	3.362474	2.020210	-1.722487
C	6.092848	3.618689	-0.471833
H	6.669979	4.529038	-0.603536
C	-4.947783	-1.669290	-0.732830
C	4.554201	-1.143052	0.199068
C	-6.890976	-0.325367	-0.146165
C	4.967210	3.380282	-1.257299
H	4.665715	4.101526	-2.011734
C	6.471790	2.677756	0.482781
H	7.344614	2.853766	1.105282
C	-6.026570	0.699262	0.299295
C	5.731321	1.515080	0.660997
H	6.020566	0.787047	1.412654
C	-8.804874	1.004744	0.434265
H	-9.882660	1.126095	0.487732
C	4.142812	-2.068277	1.165163
H	3.284824	-1.841392	1.790563
C	-4.132780	-0.522908	-0.234496
C	-7.962916	2.032108	0.880898
H	-8.389508	2.947166	1.279613
C	4.829413	-3.266942	1.318594
H	4.498447	-3.976774	2.071493

Supplementary Table 66. Cartesian coordinates of optimized DPASQ in the excited (π,π*) state calculated at PBE0-D3/6-31G(d,p) level with the SMD model of dioxane.

	X	Y	Z
O	-4.411128	-2.495968	-1.592081
N	3.884331	0.071758	0.053021
N	-6.235846	-1.358921	-0.872456
H	-6.801182	-2.067382	-1.321073

C	-0.356431	0.197397	0.118051
C	-1.787686	0.284256	0.172991
H	-2.213162	1.126229	0.714516
C	-2.665366	-0.605113	-0.395502
H	-2.312553	-1.470847	-0.949386
C	-6.586777	1.725700	1.083048
H	-5.926807	2.453500	1.545956
N	-4.634185	0.549476	0.339642
C	1.722371	-0.874271	-0.585782
H	2.236604	-1.661922	-1.128539
C	4.605526	1.249984	-0.181700
C	-8.227892	-0.186500	-0.133121
H	-8.860501	-0.933072	-0.607886
C	6.354460	-2.667726	-0.217534
H	7.260385	-2.900973	-0.768817
C	5.821307	-3.595736	0.677498
H	6.318057	-4.547952	0.835619
C	0.427920	1.181639	0.771888
H	-0.078434	1.976449	1.312765
C	1.805782	1.147182	0.754531
H	2.382396	1.901767	1.281225
C	0.345223	-0.833271	-0.562405
H	-0.208560	-1.602258	-1.090557
C	2.473853	0.114553	0.072711
C	5.723999	-1.451927	-0.429519
H	6.121263	-0.742859	-1.148285
C	4.094940	2.218945	-1.063174
H	3.161143	2.030045	-1.582114
C	6.013358	3.620175	-0.630252
H	6.560092	4.541812	-0.803861
C	-4.868279	-1.530867	-0.980516
C	4.541510	-1.147545	0.267570
C	-6.842890	-0.313571	-0.211117
C	4.800765	3.391746	-1.280646
H	4.405928	4.129338	-1.972685
C	6.518159	2.660758	0.248291
H	7.452686	2.839377	0.771541
C	-5.979087	0.646073	0.398771
C	5.824575	1.483538	0.479117
H	6.201644	0.751046	1.185211

C	-8.788328	0.889266	0.549014
H	-9.868553	0.983908	0.607323
C	4.002011	-2.084766	1.165707
H	3.096093	-1.842426	1.711194
C	-4.082130	-0.485056	-0.317011
C	-7.961857	1.844525	1.156411
H	-8.405007	2.682252	1.687617
C	4.644241	-3.296729	1.363898
H	4.227745	-4.010442	2.068132

Supplementary Table 67. Cartesian coordinates of optimized DPASQ in the excited (n,π*) state calculated at PBE0-D3/6-31G(d,p) level with the SMD model of dioxane.

	X	Y	Z
O	-4.461141	-2.609296	-1.383793
N	3.890830	0.079067	0.043947
N	-6.287867	-1.412841	-0.750126
H	-6.867458	-2.146901	-1.134352
C	-0.357435	0.184917	0.087678
C	-1.791534	0.269380	0.133221
H	-2.199949	1.159797	0.608028
C	-2.676540	-0.660970	-0.359091
H	-2.337007	-1.572859	-0.838097
C	-6.639618	1.838423	0.957316
H	-5.986831	2.605406	1.358867
N	-4.755201	0.471567	0.235897
C	1.731377	-0.913419	-0.527949
H	2.246887	-1.741044	-1.005603
C	4.628549	1.266254	-0.156569
C	-8.285553	-0.174598	-0.096561
H	-8.916552	-0.958823	-0.507075
C	6.372709	-2.655449	-0.321366
H	7.254737	-2.878343	-0.915491
C	5.908606	-3.577996	0.613670
H	6.427823	-4.520462	0.759770
C	0.416297	1.215499	0.663062
H	-0.092002	2.051384	1.137999
C	1.798613	1.183682	0.659676
H	2.362298	1.987008	1.123986
C	0.350601	-0.882857	-0.507832
H	-0.191842	-1.696240	-0.980493

C	2.483430	0.116985	0.059933
C	5.706488	-1.452193	-0.519335
H	6.063140	-0.742068	-1.258962
C	4.203084	2.215569	-1.094354
H	3.303263	2.027617	-1.671884
C	6.092286	3.624008	-0.554720
H	6.659084	4.537311	-0.708955
C	-4.944036	-1.615794	-0.860325
C	4.567363	-1.145443	0.235625
C	-6.908205	-0.310532	-0.169975
C	4.927532	3.386527	-1.281230
H	4.583754	4.112408	-2.013017
C	6.521274	2.676381	0.371862
H	7.423760	2.850419	0.951406
C	-6.061779	0.704375	0.361212
C	5.794615	1.510264	0.579849
H	6.124997	0.780761	1.312794
C	-8.847762	0.955669	0.497851
H	-9.926698	1.053326	0.550972
C	4.107039	-2.069624	1.181955
H	3.228187	-1.833319	1.773834
C	-4.071569	-0.530141	-0.290868
C	-8.020752	1.950277	1.018576
H	-8.458130	2.829899	1.481328
C	4.770464	-3.277793	1.358751
H	4.400067	-3.983869	2.096898

Supplementary Table 68. Cartesian coordinates of optimized DPASQ in the ground state calculated at PBE0-D3/6-31G(d,p) level with the SMD model of THF.

	X	Y	Z
O	4.474060	-2.661279	1.273246
N	-3.860310	0.078421	-0.037425
N	6.301993	-1.442555	0.697959
H	6.888149	-2.187714	1.057979
C	0.372896	0.161904	-0.072188
C	1.819535	0.244895	-0.107612
H	2.229857	1.164517	-0.525043
C	2.699572	-0.687512	0.319729
H	2.371551	-1.627670	0.751801
C	6.601645	1.847754	-0.879586

H	5.927457	2.614021	-1.250960
N	4.669157	0.559425	-0.270441
C	-1.714230	-0.978191	0.440454
H	-2.230859	-1.843074	0.843930
C	-4.604467	1.270500	0.140717
C	8.281675	-0.171085	0.097771
H	8.925031	-0.959610	0.479114
C	-6.356623	-2.629844	0.416295
H	-7.211248	-2.849281	1.050382
C	-5.955399	-3.542593	-0.557639
H	-6.497410	-4.474045	-0.692444
C	-0.385049	1.242790	-0.555474
H	0.131107	2.112566	-0.955059
C	-1.769281	1.222797	-0.553292
H	-2.323844	2.068566	-0.946622
C	-0.333195	-0.950404	0.425060
H	0.208055	-1.804617	0.821545
C	-2.460870	0.108843	-0.051974
C	-5.660472	-1.439920	0.598334
H	-5.966689	-0.735094	1.365512
C	-4.239238	2.193293	1.127745
H	-3.378187	1.990187	1.757419
C	-6.094819	3.611884	0.503973
H	-6.672913	4.520435	0.645070
C	4.950027	-1.641816	0.786663
C	-4.557365	-1.142184	-0.209821
C	6.893227	-0.321334	0.162960
C	-4.977005	3.359620	1.297697
H	-4.683239	4.067834	2.067550
C	-6.464742	2.687174	-0.470930
H	-7.330991	2.874393	-1.099535
C	6.033533	0.687715	-0.325294
C	-5.722685	1.526362	-0.660617
H	-6.004789	0.812017	-1.428256
C	8.813153	0.983929	-0.453868
H	9.891535	1.102194	-0.505088
C	-4.161540	-2.052893	-1.196403
H	-3.313104	-1.818036	-1.832135
C	4.136523	-0.515027	0.242901
C	7.975165	1.996299	-0.944013

H	8.406069	2.895408	-1.373650
C	-4.853090	-3.248426	-1.358369
H	-4.535176	-3.946986	-2.127459

Supplementary Table 69. Cartesian coordinates of optimized DPASQ in the excited (π,π*) state calculated at PBE0-D3/6-31G(d,p) level with the SMD model of THF.

	X	Y	Z
O	4.424285	-2.625631	1.358642
N	-3.862514	0.076253	-0.044744
N	6.248582	-1.429999	0.743504
H	6.821100	-2.173977	1.123713
C	0.371189	0.213672	-0.091031
C	1.792963	0.307033	-0.138482
H	2.212385	1.199508	-0.598046
C	2.681599	-0.635546	0.344459
H	2.325640	-1.547730	0.815427
C	6.601369	1.821304	-0.928463
H	5.945808	2.591981	-1.324489
N	4.646888	0.586153	-0.290249
C	-1.707006	-0.928467	0.498696
H	-2.217329	-1.766066	0.964033
C	-4.607879	1.254319	0.154495
C	8.239745	-0.199919	0.110504
H	8.868549	-0.989020	0.516191
C	-6.326324	-2.659688	0.333222
H	-7.207216	-2.889552	0.925363
C	-5.845518	-3.584104	-0.594876
H	-6.357242	-4.531343	-0.736058
C	-0.417443	1.261379	-0.643176
H	0.088130	2.105331	-1.105101
C	-1.791714	1.224810	-0.629203
H	-2.364614	2.028088	-1.081790
C	-0.333094	-0.883631	0.483133
H	0.219111	-1.699899	0.937268
C	-2.467053	0.123792	-0.057308
C	-5.676237	-1.448458	0.521860
H	-6.035437	-0.737466	1.258999
C	-4.167934	2.214654	1.078609
H	-3.266673	2.031103	1.654787
C	-6.076403	3.595302	0.543758

H	-6.647804	4.506170	0.694968
C	4.883305	-1.609557	0.837279
C	-4.530219	-1.149931	-0.231028
C	6.853917	-0.329886	0.177883
C	-4.902500	3.377252	1.264996
H	-4.561934	4.111736	1.988799
C	-6.513574	2.637818	-0.372576
H	-7.420684	2.806075	-0.945523
C	5.989492	0.684055	-0.342510
C	-5.787451	1.472811	-0.573878
H	-6.113982	0.737378	-1.302175
C	8.801994	0.931960	-0.472482
H	9.882451	1.029457	-0.523271
C	-4.046511	-2.077072	-1.166990
H	-3.171760	-1.833082	-1.761351
C	4.090549	-0.507240	0.273617
C	7.976510	1.941662	-0.991678
H	8.422180	2.822528	-1.445589
C	-4.704836	-3.286270	-1.340717
H	-4.331271	-3.995348	-2.073542

Supplementary Table 70. Cartesian coordinates of optimized DPASQ in the excited (n,π*) state calculated at PBE0-D3/6-31G(d,p) level with the SMD model of THF.

	X	Y	Z
O	4.463474	-2.605154	1.397776
N	-3.891087	0.079097	-0.045443
N	6.291784	-1.409345	0.760229
H	6.880441	-2.136561	1.148182
C	0.358810	0.177998	-0.093235
C	1.795032	0.262044	-0.139235
H	2.202768	1.150395	-0.619135
C	2.678087	-0.666627	0.359959
H	2.335448	-1.574995	0.844431
C	6.633361	1.833966	-0.969591
H	5.978625	2.596233	-1.377776
N	4.751670	0.466466	-0.242435
C	-1.732023	-0.915574	0.527428
H	-2.247058	-1.741424	1.009073
C	-4.625893	1.267959	0.159127
C	8.284621	-0.168644	0.102600

H	8.914474	-0.949640	0.520744
C	-6.376217	-2.653835	0.323629
H	-7.255822	-2.877150	0.921411
C	-5.917543	-3.574746	-0.616879
H	-6.438886	-4.516164	-0.763401
C	-0.413577	1.208131	-0.672283
H	0.095837	2.041840	-1.150086
C	-1.796917	1.177795	-0.667683
H	-2.358528	1.980943	-1.135255
C	-0.350128	-0.887489	0.506202
H	0.191195	-1.700788	0.980810
C	-2.483096	0.114191	-0.063324
C	-5.706882	-1.451466	0.521964
H	-6.059867	-0.744139	1.266286
C	-4.199025	2.211935	1.102291
H	-3.301446	2.019469	1.682157
C	-6.083459	3.629588	0.564895
H	-6.647682	4.544188	0.722017
C	4.950538	-1.614520	0.869020
C	-4.570598	-1.144066	-0.237839
C	6.906244	-0.308266	0.173868
C	-4.920292	3.385115	1.293147
H	-4.575430	4.106679	2.028875
C	-6.513963	2.686873	-0.367175
H	-7.414809	2.866252	-0.947875
C	6.059070	0.701569	-0.366379
C	-5.790047	1.518776	-0.578842
H	-6.122174	0.794671	-1.316612
C	8.843455	0.959786	-0.498506
H	9.922508	1.060460	-0.549811
C	-4.115864	-2.066209	-1.189528
H	-3.240092	-1.830155	-1.786382
C	4.075161	-0.535673	0.292015
C	8.014946	1.949735	-1.028701
H	8.451177	2.827466	-1.496494
C	-4.781974	-3.273673	-1.367028
H	-4.415987	-3.977905	-2.109321

Supplementary Table 71. Cartesian coordinates of optimized DPASQ in the ground state calculated at PBE0-D3/6-31G(d,p) level with the SMD model of DCM.

	X	Y	Z
O	4.478334	-2.687905	1.221024
N	-3.859922	0.079282	-0.036613
N	6.303738	-1.452285	0.677411
H	6.889725	-2.201991	1.028349
C	0.373493	0.154608	-0.079999
C	1.820236	0.236820	-0.115991
H	2.229863	1.160490	-0.525010
C	2.700965	-0.699556	0.301812
H	2.373054	-1.644845	0.722826
C	6.601417	1.862126	-0.849098
H	5.927202	2.632492	-1.211876
N	4.669823	0.559905	-0.265535
C	-1.714836	-0.982677	0.434855
H	-2.232049	-1.847002	0.838707
C	-4.601466	1.274028	0.137893
C	8.282875	-0.167271	0.105564
H	8.925977	-0.960229	0.477966
C	-6.359710	-2.621886	0.442329
H	-7.209825	-2.837821	1.083629
C	-5.968760	-3.537468	-0.533332
H	-6.514456	-4.467618	-0.662157
C	-0.383539	1.237466	-0.560809
H	0.133444	2.106944	-0.959953
C	-1.767829	1.219665	-0.556583
H	-2.321422	2.066923	-0.947987
C	-0.333665	-0.957324	0.416973
H	0.206850	-1.812563	0.812263
C	-2.460703	0.106417	-0.054853
C	-5.658687	-1.433459	0.616589
H	-5.956738	-0.726340	1.384880
C	-4.236724	2.196986	1.124987
H	-3.377990	1.992362	1.757327
C	-6.086761	3.619770	0.493617
H	-6.662852	4.530056	0.631745
C	4.953005	-1.656967	0.755285
C	-4.561303	-1.140348	-0.201042
C	6.894400	-0.321544	0.162199
C	-4.971959	3.365629	1.291260
H	-4.678714	4.074095	2.061075

C	-6.456295	2.694745	-0.481340
H	-7.320144	2.883453	-1.112790
C	6.033966	0.692345	-0.314364
C	-5.716575	1.531615	-0.667228
H	-5.998184	0.816888	-1.434709
C	8.813634	0.997181	-0.426622
H	9.891884	1.119066	-0.471525
C	-4.175788	-2.053813	-1.189198
H	-3.331765	-1.822324	-1.831997
C	4.137869	-0.523920	0.229138
C	7.974877	2.014738	-0.905468
H	8.405391	2.921110	-1.319970
C	-4.871984	-3.247841	-1.343550
H	-4.562305	-3.948624	-2.113967

Supplementary Table 72. Cartesian coordinates of optimized DPASQ in the excited (π,π*) state calculated at PBE0-D3/6-31G(d,p) level with the SMD model of DCM.

	X	Y	Z
O	4.426112	-2.637271	1.336644
N	-3.861557	0.076636	-0.044427
N	6.249327	-1.433683	0.736641
H	6.821472	-2.179583	1.113887
C	0.371798	0.209954	-0.095012
C	1.793135	0.303292	-0.142740
H	2.211602	1.198427	-0.597944
C	2.683005	-0.641581	0.335340
H	2.326727	-1.556400	0.801062
C	6.603397	1.826978	-0.917217
H	5.948625	2.599368	-1.311257
N	4.648306	0.586336	-0.290176
C	-1.706820	-0.933271	0.491725
H	-2.217322	-1.772281	0.954202
C	-4.606142	1.256181	0.153336
C	8.241046	-0.198374	0.116086
H	8.868935	-0.989300	0.519551
C	-6.328514	-2.654929	0.347168
H	-7.206587	-2.883044	0.944145
C	-5.854951	-3.580067	-0.584122
H	-6.369576	-4.526118	-0.722764
C	-0.416667	1.260880	-0.642275

H	0.089257	2.106191	-1.101327
C	-1.790775	1.225518	-0.626952
H	-2.363315	2.031121	-1.075725
C	-0.333006	-0.889567	0.475115
H	0.218954	-1.708027	0.925606
C	-2.466930	0.122429	-0.058895
C	-5.674652	-1.445012	0.532412
H	-6.028181	-0.733101	1.271400
C	-4.169115	2.213983	1.081312
H	-3.270711	2.028020	1.661191
C	-6.073517	3.598050	0.540192
H	-6.644536	4.509324	0.690473
C	4.885182	-1.615445	0.823820
C	-4.532380	-1.148978	-0.226980
C	6.855144	-0.329608	0.178630
C	-4.903098	3.377359	1.266511
H	-4.565012	4.110126	1.993212
C	-6.507895	2.642655	-0.379827
H	-7.412334	2.812960	-0.956376
C	5.990870	0.685970	-0.338688
C	-5.782165	1.476960	-0.579849
H	-6.106339	0.742717	-1.310408
C	8.803917	0.937137	-0.459482
H	9.884395	1.035992	-0.506735
C	-4.056103	-2.076447	-1.166325
H	-3.184464	-1.833789	-1.765794
C	4.091584	-0.511462	0.266332
C	7.978713	1.948799	-0.975880
H	8.424817	2.832386	-1.424032
C	-4.717981	-3.284392	-1.336686
H	-4.350322	-3.993900	-2.072075

Supplementary Table 73. Cartesian coordinates of optimized DPASQ in the excited (n,π*) state calculated at PBE0-D3/6-31G(d,p) level with the SMD model of DCM.

	X	Y	Z
O	4.470269	-2.623748	1.371650
N	-3.890741	0.079453	-0.044146
N	6.294767	-1.412811	0.753040
H	6.883927	-2.140697	1.138984
C	0.359528	0.168006	-0.099209

C	1.796137	0.250508	-0.146120
H	2.203728	1.140543	-0.623088
C	2.679548	-0.679978	0.349077
H	2.337820	-1.591249	0.829067
C	6.629994	1.841153	-0.958343
H	5.973868	2.602854	-1.365340
N	4.751558	0.461204	-0.244368
C	-1.732870	-0.921912	0.523180
H	-2.248812	-1.747164	1.004843
C	-4.621610	1.271330	0.159543
C	8.285263	-0.160474	0.110539
H	8.915979	-0.941564	0.527202
C	-6.385039	-2.643855	0.336480
H	-7.263015	-2.863388	0.938040
C	-5.934073	-3.566950	-0.605840
H	-6.459875	-4.506271	-0.749888
C	-0.411474	1.200590	-0.675951
H	0.099135	2.033824	-1.153295
C	-1.794977	1.173147	-0.669438
H	-2.355412	1.978152	-1.135188
C	-0.350746	-0.896955	0.499697
H	0.189714	-1.711819	0.972640
C	-2.482731	0.110395	-0.065051
C	-5.709797	-1.444090	0.531603
H	-6.056731	-0.735021	1.277125
C	-4.194140	2.212273	1.105498
H	-3.299274	2.015321	1.688068
C	-6.070991	3.638355	0.563149
H	-6.631998	4.555094	0.719370
C	4.954166	-1.625609	0.853225
C	-4.575486	-1.141539	-0.233218
C	6.907080	-0.306062	0.175848
C	-4.911262	3.388348	1.295314
H	-4.566079	4.107665	2.033082
C	-6.502256	2.698466	-0.371653
H	-7.400355	2.882255	-0.955212
C	6.058191	0.703543	-0.362759
C	-5.782174	1.527601	-0.582224
H	-6.114562	0.805551	-1.321908
C	8.841907	0.973378	-0.482613

H	9.920757	1.078629	-0.529034
C	-4.128641	-2.065717	-1.186775
H	-3.254554	-1.833308	-1.787522
C	4.076810	-0.546434	0.282534
C	8.011569	1.962765	-1.011254
H	8.446067	2.844596	-1.472907
C	-4.800427	-3.270682	-1.361089
H	-4.440552	-3.976544	-2.104815

Supplementary Table 74. Cartesian coordinates of optimized DPASQ in the ground state calculated at PBE0-D3/6-31G(d,p) level with the SMD model of ACN.

	X	Y	Z
O	4.474480	-2.672198	1.250589
N	-3.858932	0.079171	-0.037921
N	6.302955	-1.443932	0.698003
H	6.890996	-2.187439	1.059936
C	0.373965	0.154742	-0.086202
C	1.820680	0.238279	-0.121651
H	2.227983	1.162099	-0.532805
C	2.701585	-0.696137	0.300976
H	2.372153	-1.638532	0.727410
C	6.605991	1.850111	-0.872844
H	5.934297	2.616335	-1.249229
N	4.671928	0.555601	-0.278393
C	-1.713755	-0.987909	0.419664
H	-2.229675	-1.856702	0.815578
C	-4.601040	1.273171	0.140637
C	8.283471	-0.166800	0.116811
H	8.923860	-0.955162	0.503222
C	-6.357368	-2.622976	0.442672
H	-7.203970	-2.841451	1.087777
C	-5.972669	-3.533982	-0.540021
H	-6.519981	-4.463000	-0.670459
C	-0.383554	1.241995	-0.556805
H	0.132880	2.115110	-0.948678
C	-1.767902	1.224287	-0.550440
H	-2.321162	2.075546	-0.933629
C	-0.332506	-0.962337	0.400649
H	0.208117	-1.822127	0.785956
C	-2.460429	0.106258	-0.058136

C	-5.654340	-1.435731	0.618616
H	-5.947365	-0.732527	1.392538
C	-4.238238	2.191725	1.132525
H	-3.380494	1.985060	1.765655
C	-6.089023	3.615719	0.505032
H	-6.666264	4.524781	0.646646
C	4.952587	-1.647627	0.773411
C	-4.561469	-1.139764	-0.204007
C	6.894493	-0.320023	0.169812
C	-4.974970	3.358991	1.303269
H	-4.683674	4.064129	2.076901
C	-6.456344	2.694901	-0.475012
H	-7.319425	2.885787	-1.106887
C	6.036379	0.687611	-0.323995
C	-5.715172	1.533119	-0.665077
H	-5.994949	0.822122	-1.436797
C	8.816421	0.990127	-0.429389
H	9.894899	1.111235	-0.471643
C	-4.182012	-2.048410	-1.198906
H	-3.341844	-1.814368	-1.845932
C	4.138905	-0.521728	0.229673
C	7.979784	2.001483	-0.925905
H	8.412317	2.901903	-1.351235
C	-4.880201	-3.241221	-1.355301
H	-4.575825	-3.938320	-2.131187

Supplementary Table 75. Cartesian coordinates of optimized DPASQ in the excited (π,π*) state calculated at PBE0-D3/6-31G(d,p) level with the SMD model of ACN.

	X	Y	Z
O	4.423926	-2.649891	1.304595
N	-3.856740	0.077273	-0.044169
N	6.249406	-1.442230	0.720208
H	6.823532	-2.191885	1.088292
C	0.374366	0.214869	-0.093206
C	1.792579	0.310367	-0.139377
H	2.208374	1.213252	-0.581828
C	2.685937	-0.641998	0.326714
H	2.328212	-1.562322	0.780298
C	6.609874	1.838054	-0.895510
H	5.958338	2.617596	-1.281226

N	4.651590	0.594012	-0.283956
C	-1.703288	-0.941433	0.472573
H	-2.212424	-1.788978	0.920613
C	-4.606444	1.256189	0.148093
C	8.242692	-0.203884	0.114810
H	8.867471	-1.001608	0.509522
C	-6.320232	-2.654454	0.370093
H	-7.192312	-2.882639	0.975821
C	-5.857894	-3.577996	-0.568501
H	-6.375291	-4.523100	-0.703713
C	-0.416093	1.275697	-0.622007
H	0.088905	2.129108	-1.067031
C	-1.789155	1.239444	-0.606216
H	-2.361598	2.052429	-1.041423
C	-0.330436	-0.896256	0.457536
H	0.221502	-1.722356	0.894050
C	-2.465831	0.124577	-0.058391
C	-5.662970	-1.445251	0.550524
H	-6.008139	-0.733916	1.294101
C	-4.184625	2.210017	1.086293
H	-3.293978	2.023870	1.678150
C	-6.086762	3.590147	0.526795
H	-6.663219	4.498556	0.674070
C	4.885795	-1.623226	0.804543
C	-4.529248	-1.149807	-0.220709
C	6.856328	-0.332537	0.175267
C	-4.924998	3.370441	1.267274
H	-4.599260	4.100951	2.001912
C	-6.506274	2.637265	-0.402762
H	-7.404454	2.806464	-0.989388
C	5.993765	0.691231	-0.330530
C	-5.773622	1.474781	-0.598559
H	-6.086249	0.741860	-1.335567
C	8.808412	0.937016	-0.447211
H	9.889138	1.033993	-0.492977
C	-4.064777	-2.074186	-1.168219
H	-3.200992	-1.830044	-1.778553
C	4.091729	-0.511288	0.259764
C	7.985509	1.957053	-0.952188
H	8.433969	2.844747	-1.389800

C	-4.729541	-3.281629	-1.333749
H	-4.371491	-3.989471	-2.075523

Supplementary Table 76. Cartesian coordinates of optimized DPASQ in the excited (n,π*) state calculated at PBE0-D3/6-31G(d,p) level with the SMD model of ACN.

	X	Y	Z
O	4.469351	-2.620722	1.378214
N	-3.890565	0.079914	-0.045494
N	6.294983	-1.411157	0.757750
H	6.887545	-2.136257	1.145316
C	0.359982	0.166408	-0.102937
C	1.797207	0.248714	-0.150020
H	2.204706	1.137470	-0.629646
C	2.679428	-0.680984	0.348905
H	2.335933	-1.590399	0.831462
C	6.627585	1.838956	-0.962707
H	5.971255	2.599152	-1.372458
N	4.749070	0.460543	-0.246821
C	-1.732854	-0.921840	0.521695
H	-2.248355	-1.746508	1.004997
C	-4.620767	1.271790	0.160160
C	8.283760	-0.159121	0.113492
H	8.913338	-0.939352	0.533472
C	-6.384335	-2.644033	0.336759
H	-7.261881	-2.863820	0.938882
C	-5.933561	-3.567142	-0.605952
H	-6.459221	-4.506618	-0.749715
C	-0.410650	1.198730	-0.680911
H	0.100101	2.031184	-1.159482
C	-1.794475	1.171792	-0.673507
H	-2.354214	1.976649	-1.140518
C	-0.350386	-0.897747	0.497513
H	0.189632	-1.712718	0.970921
C	-2.482492	0.110193	-0.067272
C	-5.709263	-1.443856	0.531421
H	-6.056323	-0.735420	1.277583
C	-4.190257	2.213048	1.104591
H	-3.293953	2.016595	1.685218
C	-6.068999	3.639232	0.567793
H	-6.629550	4.556015	0.725577

C	4.955491	-1.624248	0.857385
C	-4.575473	-1.141024	-0.234218
C	6.905170	-0.305218	0.177991
C	-4.906842	3.389328	1.296499
H	-4.559337	4.108794	2.033067
C	-6.503308	2.698912	-0.365475
H	-7.403273	2.882500	-0.946253
C	6.056357	0.702488	-0.364206
C	-5.783796	1.527821	-0.578031
H	-6.119000	0.806161	-1.316920
C	8.839657	0.973523	-0.482331
H	9.918572	1.079382	-0.528036
C	-4.128826	-2.065096	-1.188106
H	-3.255552	-1.832653	-1.790144
C	4.077335	-0.547497	0.282932
C	8.009258	1.961396	-1.014759
H	8.443776	2.842217	-1.478416
C	-4.800378	-3.270451	-1.362113
H	-4.440834	-3.976138	-2.106199

Supplementary Table 77. Cartesian coordinates of optimized DPASQ in the ground state calculated at PBE0-D3/6-31G(d,p) level with the SMD model of MeOH.

	X	Y	Z
O	4.478792	-2.651712	1.297969
N	-3.858357	0.078231	-0.038030
N	6.299872	-1.431260	0.718186
H	6.886609	-2.171015	1.091678
C	0.373424	0.153728	-0.082055
C	1.819493	0.237781	-0.116868
H	2.226098	1.159304	-0.533516
C	2.700602	-0.693491	0.312792
H	2.366244	-1.630879	0.746150
C	6.602975	1.836876	-0.903426
H	5.932332	2.598308	-1.291213
N	4.670245	0.550788	-0.287066
C	-1.714373	-0.988764	0.422121
H	-2.230866	-1.857328	0.818236
C	-4.600263	1.271537	0.144313
C	8.281135	-0.166336	0.115540
H	8.921328	-0.949157	0.513432

C	-6.358109	-2.625176	0.424139
H	-7.207116	-2.846106	1.065467
C	-5.969795	-3.532578	-0.560281
H	-6.516622	-4.461286	-0.695992
C	-0.383484	1.240808	-0.554105
H	0.133128	2.113726	-0.946443
C	-1.767571	1.223344	-0.548651
H	-2.320599	2.074269	-0.933371
C	-0.333336	-0.963230	0.404680
H	0.206371	-1.823319	0.790813
C	-2.460153	0.105396	-0.056673
C	-5.655988	-1.438588	0.606814
H	-5.951827	-0.738259	1.382496
C	-4.236478	2.186874	1.138584
H	-3.378625	1.977914	1.771104
C	-6.087179	3.612956	0.516537
H	-6.664202	4.521832	0.661246
C	4.955010	-1.628797	0.798086
C	-4.560306	-1.139887	-0.210783
C	6.892450	-0.315815	0.171674
C	-4.972782	3.353671	1.313210
H	-4.680868	4.056533	2.088867
C	-6.455376	2.695187	-0.465846
H	-7.318948	2.888228	-1.096629
C	6.033299	0.683011	-0.335888
C	-5.714774	1.533863	-0.659825
H	-5.995240	0.825366	-1.433815
C	8.813551	0.981337	-0.448882
H	9.892025	1.101684	-0.494594
C	-4.177123	-2.044707	-1.207499
H	-3.334974	-1.808063	-1.851280
C	4.137084	-0.519127	0.239166
C	7.976210	1.985367	-0.959772
H	8.409201	2.878844	-1.399335
C	-4.874587	-3.236893	-1.370558
H	-4.567432	-3.931295	-2.147954

Supplementary Table 78. Cartesian coordinates of optimized DPASQ in the excited (π,π*) state calculated at PBE0-D3/6-31G(d,p) level with the SMD model of MeOH.

	X	Y	Z

O	4.424765	-2.645045	1.317729
N	-3.856789	0.076896	-0.044169
N	6.245154	-1.439236	0.722315
H	6.817547	-2.190138	1.091609
C	0.371939	0.213362	-0.088731
C	1.792125	0.309329	-0.134854
H	2.206540	1.211181	-0.580347
C	2.683353	-0.640029	0.333413
H	2.320313	-1.557135	0.789141
C	6.608809	1.835507	-0.901495
H	5.958860	2.615916	-1.288194
N	4.649883	0.594776	-0.283308
C	-1.705245	-0.940675	0.478307
H	-2.215209	-1.786158	0.929601
C	-4.605693	1.255522	0.148422
C	8.240010	-0.206423	0.110432
H	8.863993	-1.004377	0.506024
C	-6.320876	-2.653801	0.361575
H	-7.195037	-2.882537	0.964257
C	-5.854532	-3.577158	-0.575138
H	-6.371263	-4.522510	-0.712115
C	-0.416227	1.272562	-0.621400
H	0.089393	2.124443	-1.068752
C	-1.789542	1.237077	-0.607046
H	-2.361435	2.048297	-1.046592
C	-0.332289	-0.895477	0.464613
H	0.218527	-1.720523	0.904635
C	-2.465843	0.124030	-0.056760
C	-5.665057	-1.444492	0.544117
H	-6.012519	-0.733544	1.287191
C	-4.181595	2.209396	1.085558
H	-3.290832	2.022583	1.677286
C	-6.083866	3.589274	0.527717
H	-6.659826	4.498093	0.675300
C	4.887034	-1.612057	0.808191
C	-4.528363	-1.149660	-0.222964
C	6.854398	-0.330450	0.172690
C	-4.921378	3.369775	1.266965
H	-4.594773	4.100587	2.001054
C	-6.505218	2.636387	-0.400917

H	-7.403948	2.806180	-0.986703
C	5.992449	0.691270	-0.332918
C	-5.773796	1.473535	-0.596945
H	-6.087057	0.740893	-1.334154
C	8.806885	0.932148	-0.455293
H	9.887736	1.027219	-0.503282
C	-4.059424	-2.073864	-1.168454
H	-3.193987	-1.829171	-1.776470
C	4.091840	-0.509737	0.265049
C	7.984607	1.951599	-0.960715
H	8.433530	2.837740	-1.401286
C	-4.723442	-3.281058	-1.336281
H	-4.362779	-3.989106	-2.076727

Supplementary Table 79. Cartesian coordinates of optimized DPASQ in the excited (n,π*) state calculated at PBE0-D3/6-31G(d,p) level with the SMD model of MeOH.

	X	Y	Z
O	4.481266	-2.632546	1.378827
N	-3.887185	0.080312	-0.044084
N	6.295673	-1.410333	0.750929
H	6.887595	-2.138417	1.134759
C	0.361006	0.161768	-0.094699
C	1.798889	0.242453	-0.139706
H	2.206978	1.133845	-0.614340
C	2.678688	-0.689732	0.354428
H	2.334130	-1.600941	0.833112
C	6.617635	1.845850	-0.962048
H	5.958427	2.605518	-1.368035
N	4.749480	0.452300	-0.240368
C	-1.732919	-0.929410	0.517059
H	-2.249950	-1.757583	0.992873
C	-4.617690	1.272617	0.159336
C	8.280822	-0.151351	0.104162
H	8.913431	-0.931438	0.520176
C	-6.384213	-2.640040	0.335759
H	-7.261022	-2.860167	0.939051
C	-5.937228	-3.560813	-0.610818
H	-6.465164	-4.498883	-0.756454
C	-0.406430	1.200104	-0.664492
H	0.105969	2.035992	-1.135441

C	-1.790218	1.175522	-0.658902
H	-2.347909	1.985178	-1.120244
C	-0.350660	-0.906563	0.495323
H	0.187410	-1.726565	0.962392
C	-2.480139	0.109225	-0.063506
C	-5.706428	-1.441889	0.532664
H	-6.050173	-0.735008	1.282083
C	-4.193165	2.210334	1.109535
H	-3.301186	2.011448	1.696298
C	-6.067784	3.638121	0.564174
H	-6.629385	4.554584	0.721025
C	4.955586	-1.627046	0.854192
C	-4.573985	-1.139538	-0.234535
C	6.903518	-0.302491	0.173411
C	-4.910784	3.386062	1.300054
H	-4.568416	4.103329	2.041336
C	-6.496034	2.700934	-0.374796
H	-7.392190	2.886582	-0.961009
C	6.052015	0.707159	-0.364928
C	-5.775381	1.530409	-0.585939
H	-6.105428	0.810920	-1.329503
C	8.832666	0.984046	-0.490093
H	9.911310	1.092241	-0.538397
C	-4.131076	-2.060802	-1.192417
H	-3.259065	-1.827715	-1.796352
C	4.077473	-0.559202	0.288801
C	7.999119	1.971054	-1.017285
H	8.430039	2.854314	-1.479851
C	-4.805323	-3.264196	-1.368648
H	-4.449056	-3.968334	-2.115947

Supplementary Table 80. Cartesian coordinates of optimized MeMQ in the ground state calculated at PBE0-D3/6-31G(d,p) level with the SMD model of *n*-hexane.

	X	Y	Z
C	-2.878208	-1.774723	-0.000092
C	-1.591793	-1.023355	-0.000013
C	-1.709855	0.455091	0.000183
N	-0.509789	1.144589	-0.000002
C	0.709143	0.486905	0.000012
C	0.704666	-0.926072	-0.000045

N	-0.466074	-1.655164	-0.000095
C	1.932437	1.172580	0.000100
C	3.121888	0.460184	0.000096
C	3.125506	-0.938413	0.000001
C	1.922271	-1.619922	-0.000063
O	-2.797490	1.020280	0.000133
H	-3.478606	-1.506042	-0.875723
H	-2.680880	-2.847534	-0.000125
H	-3.478688	-1.506089	0.875492
H	1.956348	2.256055	0.000211
H	4.061747	1.004405	0.000176
H	4.064288	-1.482929	-0.000014
H	1.876789	-2.704655	-0.000123
C	-0.561798	2.595171	-0.000138
H	-0.072484	2.994974	0.892812
H	-0.071910	2.994787	-0.892851
H	-1.611187	2.884136	-0.000479

Supplementary Table 81. Cartesian coordinates of optimized MeMQ in the excited (n,π*) state calculated at PBE0-D3/6-31G(d,p) level with the SMD model of *n*-hexane.

	X	Y	Z
C	2.926838	-1.798988	0.000137
C	1.670904	-1.001309	-0.000034
C	1.724296	0.445781	-0.000283
N	0.512766	1.128489	-0.000089
C	-0.723055	0.481487	-0.000076
C	-0.734944	-0.955121	-0.000044
N	0.460669	-1.532610	0.000066
C	-1.936566	1.157141	-0.000061
C	-3.150617	0.456026	-0.000058
C	-3.154334	-0.934305	-0.000028
C	-1.962672	-1.645429	-0.000003
O	2.814090	1.033325	0.000214
H	3.535146	-1.567084	0.881788
H	2.693679	-2.865496	0.000169
H	3.535304	-1.567157	-0.881422
H	-1.948456	2.240937	-0.000085
H	-4.083047	1.010292	-0.000066
H	-4.095022	-1.477011	-0.000003
H	-1.943233	-2.729388	0.000047

C	0.552277	2.575757	0.000068
H	0.055238	2.972339	-0.892007
H	0.055215	2.972146	0.892214
H	1.595644	2.886420	0.000112

Supplementary Table 82. Cartesian coordinates of optimized MeMQ in the excited (π,π*) state calculated at PBE0-D3/6-31G(d,p) level with the SMD model of *n*-hexane.

	X	Y	Z
C	2.940336	-1.736135	-0.001753
C	1.650672	-0.990683	0.000478
C	1.709108	0.440648	0.001810
N	0.461050	1.158402	-0.003061
C	-0.717286	0.478588	-0.002454
C	-0.659187	-0.975430	-0.001824
N	0.493633	-1.682666	-0.001557
C	-1.963836	1.152197	-0.000552
C	-3.153066	0.437399	0.000853
C	-3.105951	-0.965542	0.002057
C	-1.884967	-1.650710	0.001602
O	2.744730	1.110407	0.005319
H	3.555127	-1.497304	0.875992
H	2.732039	-2.808174	-0.003464
H	3.554339	-1.494455	-0.879251
H	-1.985629	2.236029	-0.001913
H	-4.103481	0.958938	0.000999
H	-4.030862	-1.535005	0.003321
H	-1.853335	-2.735220	0.003405
C	0.504659	2.603093	-0.001281
H	0.007057	3.001431	-0.892715
H	0.009319	3.000265	0.891958
H	1.551922	2.899540	-0.002169

Supplementary Table 83. Cartesian coordinates of optimized MeMQ in the ground state calculated at PBE0-D3/6-31G(d,p) level with the SMD model of dioxane.

	X	Y	Z
C	-2.877936	-1.774571	-0.000086
C	-1.592143	-1.022747	-0.000015
C	-1.709881	0.455648	0.000167
N	-0.509781	1.144529	-0.000010
C	0.708894	0.486596	0.000009

C	0.704313	-0.926219	-0.000051
N	-0.466553	-1.654969	-0.000101
C	1.932168	1.172180	0.000106
C	3.121369	0.459649	0.000102
C	3.124894	-0.938843	0.000001
C	1.921756	-1.620200	-0.000069
O	-2.797615	1.021230	0.000156
H	-3.478676	-1.507311	-0.875965
H	-2.680286	-2.847420	-0.000117
H	-3.478749	-1.507356	0.875754
H	1.956802	2.255677	0.000221
H	4.061339	1.003807	0.000188
H	4.063677	-1.483500	-0.000013
H	1.876823	-2.705040	-0.000131
C	-0.559744	2.595064	-0.000143
H	-0.070052	2.994277	0.892891
H	-0.069447	2.994092	-0.892920
H	-1.608316	2.886674	-0.000503

Supplementary Table 84. Cartesian coordinates of optimized MeMQ in the excited (n,π*) state calculated at PBE0-D3/6-31G(d,p) level with the SMD model of dioxane.

	X	Y	Z
C	2.926331	-1.799024	0.000128
C	1.671159	-1.000658	-0.000029
C	1.724209	0.446268	-0.000260
N	0.512692	1.128606	-0.000076
C	-0.722777	0.481261	-0.000071
C	-0.734492	-0.955109	-0.000035
N	0.461170	-1.532829	0.000069
C	-1.936401	1.156747	-0.000068
C	-3.150112	0.455393	-0.000065
C	-3.153692	-0.934791	-0.000030
C	-1.961879	-1.645541	0.000001
O	2.814131	1.034340	0.000190
H	3.535036	-1.568648	0.881972
H	2.692311	-2.865423	0.000156
H	3.535183	-1.568715	-0.881630
H	-1.949080	2.240587	-0.000095
H	-4.082691	1.009582	-0.000080
H	-4.094324	-1.477745	-0.000006

H	-1.942344	-2.729596	0.000051
C	0.550411	2.575827	0.000070
H	0.053155	2.971953	-0.892107
H	0.053099	2.971771	0.892295
H	1.593030	2.888840	0.000132

Supplementary Table 85. Cartesian coordinates of optimized MeMQ in the excited (π,π*) state calculated at PBE0-D3/6-31G(d,p) level with the SMD model of dioxane.

	X	Y	Z
C	2.927271	-1.756868	-0.000021
C	1.655977	-0.984961	0.000049
C	1.713680	0.448335	0.000117
N	0.462798	1.159997	-0.000123
C	-0.714870	0.479676	-0.000122
C	-0.655596	-0.973905	-0.000100
N	0.497736	-1.677259	-0.000067
C	-1.962385	1.151240	-0.000061
C	-3.148454	0.433952	-0.000025
C	-3.100486	-0.970011	0.000022
C	-1.880685	-1.652396	0.000022
O	2.742459	1.130041	0.000310
H	2.982088	-2.413976	0.878913
H	2.981972	-2.414014	-0.878932
H	3.795474	-1.096306	-0.000084
H	-1.987696	2.234867	-0.000115
H	-4.100120	0.953536	-0.000033
H	-4.025431	-1.539562	0.000058
H	-1.846768	-2.736887	0.000095
C	0.498219	2.605500	-0.000042
H	-0.000839	2.999622	-0.892453
H	-0.000763	2.999569	0.892435
H	1.542654	2.910292	-0.000067

Supplementary Table 86. Cartesian coordinates of optimized MeMQ in the ground state calculated at PBE0-D3/6-31G(d,p) level with the SMD model of THF.

	X	Y	Z
C	-2.880161	-1.772424	-0.000004
C	-1.593514	-1.021953	-0.000086
C	-1.708426	0.456534	-0.000191
N	-0.510249	1.144514	-0.000114

C	0.707888	0.485392	-0.000025
C	0.704015	-0.927554	-0.000198
N	-0.467846	-1.655538	-0.000239
C	1.931388	1.171886	0.000228
C	3.121023	0.459523	0.000217
C	3.125366	-0.940015	-0.000020
C	1.922178	-1.622118	-0.000206
O	-2.798365	1.021264	0.000492
H	-3.479670	-1.505328	-0.876982
H	-2.686075	-2.846201	0.000140
H	-3.479717	-1.505076	0.876860
H	1.955223	2.255447	0.000446
H	4.060743	1.004288	0.000409
H	4.064499	-1.484374	-0.000026
H	1.880794	-2.707455	-0.000337
C	-0.555513	2.596940	-0.000033
H	-0.063215	2.992274	0.892876
H	-0.062183	2.992341	-0.892335
H	-1.602290	2.893877	-0.000614

Supplementary Table 87. Cartesian coordinates of optimized MeMQ in the excited (n,π*) state calculated at PBE0-D3/6-31G(d,p) level with the SMD model of THF.

	X	Y	Z
C	-2.928623	-1.796922	-0.000094
C	-1.672175	-0.999765	-0.000081
C	-1.721236	0.446955	-0.000069
N	-0.512039	1.129853	-0.000069
C	0.722168	0.480668	-0.000027
C	0.732863	-0.955614	-0.000126
N	-0.463064	-1.536885	-0.000153
C	1.936923	1.156338	0.000138
C	3.150225	0.453824	0.000141
C	3.153543	-0.936992	0.000020
C	1.959700	-1.646843	-0.000088
O	-2.813236	1.035449	0.000245
H	-3.536831	-1.567361	-0.882772
H	-2.694997	-2.863509	0.000099
H	-3.537004	-1.567070	0.882386
H	1.949419	2.240369	0.000235
H	4.083057	1.008135	0.000241

H	4.093761	-1.481053	0.000024
H	1.939578	-2.731302	-0.000153
C	-0.546853	2.579332	0.000064
H	-0.048330	2.972820	0.892213
H	-0.047914	2.972954	-0.891790
H	-1.588339	2.895768	-0.000156

Supplementary Table 88. Cartesian coordinates of optimized MeMQ in the excited (π,π*) state calculated at PBE0-D3/6-31G(d,p) level with the SMD model of THF.

	X	Y	Z
C	-2.939387	-1.735093	-0.000212
C	-1.650692	-0.988945	0.000062
C	-1.708367	0.441518	0.000513
N	-0.462041	1.158374	-0.000245
C	0.715151	0.480295	-0.000136
C	0.660631	-0.977473	-0.000271
N	-0.491841	-1.683946	-0.000384
C	1.960928	1.154779	0.000167
C	3.147946	0.438967	0.000171
C	3.105139	-0.966770	0.000005
C	1.888998	-1.653495	-0.000150
O	-2.749024	1.106542	0.000736
H	-3.555194	-1.495978	-0.877950
H	-2.735014	-2.808321	-0.000378
H	-3.555345	-1.496244	0.877491
H	1.983026	2.238439	0.000252
H	4.098668	0.960791	0.000285
H	4.032233	-1.532859	-0.000003
H	1.863253	-2.738561	-0.000209
C	-0.500085	2.605065	-0.000207
H	-0.000128	2.998046	0.891885
H	0.000358	2.997983	-0.892046
H	-1.544070	2.910283	-0.000462

Supplementary Table 89. Cartesian coordinates of optimized MeMQ in the ground state calculated at PBE0-D3/6-31G(d,p) level with the SMD model of DCM.

	X	Y	Z
C	2.880396	-1.772089	-0.000059
C	1.593324	-1.022261	-0.000040
C	1.707418	0.455820	-0.000037

N	0.510688	1.144095	-0.000021
C	-0.707998	0.485296	0.000016
C	-0.704246	-0.927659	0.000021
N	0.467508	-1.655841	-0.000009
C	-1.931287	1.172385	0.000048
C	-3.121076	0.460227	0.000081
C	-3.125602	-0.939564	0.000084
C	-1.922681	-1.622121	0.000054
O	2.798229	1.020745	-0.000072
H	3.479376	-1.504522	0.877231
H	2.687495	-2.846070	-0.000133
H	3.479426	-1.504398	-0.877275
H	-1.954412	2.255908	0.000048
H	-4.060706	1.005090	0.000106
H	-4.064852	-1.483729	0.000108
H	-1.882036	-2.707504	0.000055
C	0.556823	2.597115	-0.000038
H	0.064182	2.992441	-0.892637
H	0.064261	2.992461	0.892595
H	1.603640	2.893700	-0.000087

Supplementary Table 90. Cartesian coordinates of optimized MeMQ in the excited (n,π*) state calculated at PBE0-D3/6-31G(d,p) level with the SMD model of DCM.

	X	Y	Z
C	-2.928748	-1.797398	0.000062
C	-1.671997	-1.000480	0.000036
C	-1.721636	0.445659	0.000028
N	-0.512510	1.129314	0.000013
C	0.722112	0.480857	-0.000023
C	0.734305	-0.955646	-0.000031
N	-0.462226	-1.535822	0.000004
C	1.936891	1.156849	-0.000044
C	3.150642	0.454928	-0.000076
C	3.154862	-0.935922	-0.000084
C	1.961140	-1.646521	-0.000060
O	-2.813931	1.034250	0.000097
H	-3.536411	-1.566587	-0.882706
H	-2.696348	-2.864206	0.000157
H	-3.536459	-1.566436	0.882756
H	1.948428	2.240883	-0.000038

H	4.083116	1.009851	-0.000094
H	4.095323	-1.479541	-0.000107
H	1.941568	-2.730979	-0.000063
C	-0.549314	2.579097	0.000037
H	-0.051278	2.973346	0.892030
H	-0.051325	2.973373	-0.891971
H	-1.591558	2.893309	0.000069

Supplementary Table 91. Cartesian coordinates of optimized MeMQ in the excited (π,π*) state calculated at PBE0-D3/6-31G(d,p) level with the SMD model of DCM.

	X	Y	Z
C	-2.940012	-1.733206	0.000060
C	-1.649673	-0.989505	0.000036
C	-1.707781	0.440171	0.000032
N	-0.462010	1.158305	0.000004
C	0.714868	0.480303	-0.000032
C	0.661261	-0.977567	-0.000034
N	-0.491338	-1.684968	-0.000002
C	1.960876	1.155432	-0.000057
C	3.147839	0.439726	-0.000081
C	3.105642	-0.966239	-0.000076
C	1.889514	-1.653470	-0.000049
O	-2.749516	1.104450	0.000110
H	-3.554998	-1.491997	-0.877762
H	-2.739139	-2.807123	0.000139
H	-3.555037	-1.491867	0.877818
H	1.982614	2.239010	-0.000060
H	4.098524	0.961603	-0.000102
H	4.033111	-1.531716	-0.000091
H	1.864247	-2.738495	-0.000041
C	-0.501139	2.605195	0.000042
H	-0.001355	2.998531	0.892009
H	-0.001390	2.998574	-0.891926
H	-1.545376	2.909480	0.000070

Supplementary Table 92. Cartesian coordinates of optimized MeMQ in the ground state calculated at PBE0-D3/6-31G(d,p) level with the SMD model of ACN.

	X	Y	Z
C	-2.881307	-1.770701	-0.000037
C	-1.593981	-1.021645	0.000053

C	-1.707137	0.456503	0.000299
N	-0.510598	1.144038	0.000111
C	0.707451	0.484603	0.000018
C	0.703826	-0.928399	0.000020
N	-0.468315	-1.656011	0.000016
C	1.930752	1.171965	-0.000060
C	3.120565	0.459825	-0.000116
C	3.125434	-0.940252	-0.000112
C	1.922482	-1.623044	-0.000049
O	-2.798510	1.021137	-0.000041
H	-3.479756	-1.503141	-0.877714
H	-2.689842	-2.845009	-0.000046
H	-3.479877	-1.503157	0.877559
H	1.953599	2.255513	-0.000057
H	4.060185	1.004757	-0.000166
H	4.064921	-1.484099	-0.000166
H	1.883389	-2.708584	-0.000061
C	-0.553688	2.597773	0.000007
H	-0.059284	2.990991	0.892506
H	-0.059432	2.990865	-0.892629
H	-1.599817	2.896806	0.000077

Supplementary Table 93. Cartesian coordinates of optimized MeMQ in the excited (n,π*) state calculated at PBE0-D3/6-31G(d,p) level with the SMD model of ACN.

	X	Y	Z
C	2.929500	-1.796339	0.000066
C	1.672520	-0.999693	-0.000163
C	1.720364	0.446509	-0.000350
N	0.512037	1.129859	-0.000028
C	-0.721806	0.480365	0.000053
C	-0.733088	-0.955937	0.000032
N	0.463414	-1.537869	-0.000012
C	-1.936914	1.156454	0.000111
C	-3.150305	0.453916	0.000153
C	-3.154188	-0.937128	0.000119
C	-1.959685	-1.647179	0.000045
O	2.813208	1.035600	-0.000102
H	3.537070	-1.566624	0.883250
H	2.696581	-2.863058	-0.000044
H	3.537418	-1.566502	-0.882843

H	-1.948659	2.240544	0.000134
H	-4.082932	1.008732	0.000221
H	-4.094418	-1.481250	0.000164
H	-1.939911	-2.731771	0.000024
C	0.546595	2.580340	-0.000023
H	0.047521	2.973282	-0.891809
H	0.047483	2.973259	0.891746
H	1.588065	2.896806	0.000009

Supplementary Table 94. Cartesian coordinates of optimized MeMQ in the excited (π,π*) state calculated at PBE0-D3/6-31G(d,p) level with the SMD model of ACN.

	X	Y	Z
C	2.938812	-1.734233	-0.000214
C	1.650073	-0.988715	0.000112
C	1.707558	0.440983	0.000608
N	0.462648	1.158208	-0.000235
C	-0.714005	0.480960	-0.000186
C	-0.661645	-0.978225	-0.000124
N	0.490491	-1.685146	-0.000235
C	-1.959457	1.156300	-0.000090
C	-3.145794	0.440440	-0.000006
C	-3.105352	-0.966569	0.000106
C	-1.891072	-1.654266	0.000107
O	2.750891	1.103979	0.000521
H	3.554795	-1.494830	0.877639
H	2.737455	-2.808102	-0.000601
H	3.554774	-1.494206	-0.877909
H	-1.981088	2.239876	-0.000207
H	-4.096467	0.962579	-0.000041
H	-4.033590	-1.530733	0.000171
H	-1.867701	-2.739510	0.000237
C	0.499712	2.605734	-0.000226
H	-0.001689	2.997258	-0.891860
H	-0.001531	2.997319	0.891480
H	1.542954	2.912628	-0.000314

Supplementary Table 95. Cartesian coordinates of optimized MeMQ in the ground state calculated at PBE0-D3/6-31G(d,p) level with the SMD model of MeOH.

	X	Y	Z
C	2.882587	-1.766469	0.000068

C	1.593497	-1.021100	-0.000101
C	1.700713	0.453777	-0.000486
N	0.512348	1.142410	-0.000187
C	-0.707668	0.483028	-0.000033
C	-0.703465	-0.929196	-0.000097
N	0.467722	-1.656547	-0.000102
C	-1.929314	1.172304	0.000156
C	-3.118857	0.460860	0.000221
C	-3.124025	-0.939475	0.000135
C	-1.922699	-1.623401	-0.000010
O	2.798596	1.020022	0.000205
H	3.480175	-1.498851	0.878753
H	2.694571	-2.841351	0.000242
H	3.480237	-1.499143	-0.878662
H	-1.952321	2.255809	0.000225
H	-4.058332	1.006112	0.000351
H	-4.063996	-1.482670	0.000203
H	-1.885241	-2.709005	-0.000039
C	0.553033	2.597326	0.000007
H	0.058077	2.988554	-0.892627
H	0.058592	2.988327	0.893028
H	1.596172	2.905071	-0.000257

Supplementary Table 96. Cartesian coordinates of optimized MeMQ in the excited (n,π*) state calculated at PBE0-D3/6-31G(d,p) level with the SMD model of MeOH.

	X	Y	Z
C	2.939628	-1.726850	0.000038
C	1.647252	-0.987757	-0.000112
C	1.703087	0.437063	-0.000386
N	0.461883	1.159061	0.000061
C	-0.712914	0.479934	0.000144
C	-0.662008	-0.978174	0.000048
N	0.491665	-1.688570	0.000078
C	-1.959763	1.155591	0.000247
C	-3.144767	0.439711	0.000210
C	-3.104838	-0.966639	0.000029
C	-1.888352	-1.653635	-0.000077
O	2.754154	1.098319	-0.000261
H	3.552391	-1.481638	0.878439
H	2.746402	-2.801969	0.000156

H	3.552459	-1.481829	-0.878368
H	-1.982144	2.239147	0.000399
H	-4.095595	0.961895	0.000329
H	-4.032596	-1.531594	-0.000005
H	-1.865443	-2.738916	-0.000227
C	0.496883	2.606843	-0.000043
H	-0.003556	2.999030	-0.892028
H	-0.003315	2.999149	0.892021
H	1.538078	2.920231	-0.000188

Supplementary Table 97. Cartesian coordinates of optimized MeMQ in the excited (π,π*) state calculated at PBE0-D3/6-31G(d,p) level with the SMD model of MeOH.

	X	Y	Z
C	2.926523	-1.801100	-0.000168
C	1.673192	-0.999273	0.000045
C	1.724551	0.441935	-0.000069
N	0.514707	1.126956	-0.000222
C	-0.720785	0.480765	-0.000203
C	-0.739452	-0.956568	-0.000098
N	0.460831	-1.527295	0.000060
C	-1.935516	1.156764	-0.000087
C	-3.149800	0.456749	0.000001
C	-3.157437	-0.933652	0.000109
C	-1.964668	-1.646722	0.000136
O	2.819865	1.033327	0.000368
H	3.533101	-1.570225	0.883405
H	2.693776	-2.867710	0.000023
H	3.532696	-1.570456	-0.884083
H	-1.945395	2.241004	-0.000146
H	-4.081158	1.013827	-0.000002
H	-4.098771	-1.475847	0.000194
H	-1.946543	-2.731257	0.000264
C	0.548891	2.577589	0.000041
H	0.050635	2.971256	-0.891886
H	0.050993	2.970978	0.892297
H	1.589971	2.895265	-0.000128

Supplementary Table 98. Cartesian coordinates of optimized MQH^+ in the ground state calculated at PBE0-D3/6-31G(d,p) level with the SMD model of DCM.

	X	Y	Z

C	-3.022658	1.334123	-0.000089
C	-1.732019	0.630408	-0.000041
C	-1.724122	-0.856566	-0.000040
N	-0.475741	-1.416316	-0.000021
C	0.711194	-0.722521	0.000012
C	0.660705	0.680616	0.000038
N	-0.594680	1.267692	0.000010
C	1.956375	-1.359465	0.000019
C	3.106298	-0.590506	0.000051
C	3.046618	0.812659	0.000079
C	1.824297	1.454872	0.000072
O	-2.760217	-1.497552	-0.000060
H	-3.599169	1.020359	0.877426
H	-2.900151	2.418527	-0.000260
H	-3.599258	1.020080	-0.877441
H	-0.445289	-2.431897	-0.000034
H	2.001030	-2.444168	-0.000005
H	4.072368	-1.085042	0.000052
H	3.961949	1.394434	0.000102
H	1.747357	2.538220	0.000089
H	-0.624275	2.288553	0.000025

Supplementary Table 99. Cartesian coordinates of optimized MQH^+ in the excited (π,π*) state calculated at PBE0-D3/6-31G(d,p) level with the SMD model of DCM.

	X	Y	Z
C	-3.092296	1.290767	-0.000076
C	-1.785947	0.600392	-0.000049
C	-1.711210	-0.838514	-0.000060
N	-0.431348	-1.417889	-0.000018
C	0.714103	-0.695363	0.000012
C	0.629741	0.718156	0.000040
N	-0.623754	1.311034	0.000020
C	1.986456	-1.330346	0.000019
C	3.152341	-0.574492	0.000057
C	3.059939	0.809623	0.000089
C	1.789854	1.457289	0.000081
O	-2.686988	-1.581262	-0.000093
H	-3.685087	1.012381	0.879909
H	-2.962620	2.375760	-0.000350
H	-3.685255	1.011954	-0.879806

H	-0.401738	-2.433072	-0.000035
H	2.018902	-2.416139	-0.000008
H	4.118291	-1.065738	0.000059
H	3.954568	1.423055	0.000117
H	1.739041	2.542203	0.000104
H	-0.672372	2.322608	0.000053

Supplementary Table 100. Cartesian coordinates of optimized MeOSQH$^+$ in the ground state calculated at PBE0-D3/6-31G(d,p) level with the SMD model of DCM.

	X	Y	Z
O	1.526579	2.878324	-0.068367
N	3.461913	1.688389	-0.076699
H	3.974569	2.563373	-0.134631
O	-6.472977	-0.545623	-0.057458
C	5.568397	0.473933	-0.074571
H	6.121615	1.406108	-0.140309
C	2.103795	1.803652	-0.038523
N	2.070300	-0.615051	0.084752
C	1.359143	0.506703	0.036505
C	4.113227	-1.923468	0.100544
H	3.534716	-2.840168	0.168697
C	-0.048533	0.585664	0.047420
H	-0.422312	1.603450	0.066315
C	4.173078	0.507518	-0.035826
C	-4.432615	-1.647966	-0.149291
H	-4.991565	-2.571946	-0.255445
C	6.219230	-0.748393	-0.026368
H	7.303892	-0.774420	-0.056444
C	-0.906247	-0.482145	0.000228
H	-0.499109	-1.491534	-0.067153
C	-5.143809	-0.439756	-0.028317
C	3.451900	-0.693196	0.050258
C	-3.067581	0.773119	0.125659
H	-2.548612	1.719649	0.238538
C	-4.447076	0.775754	0.113421
H	-4.981439	1.712945	0.215169
C	-2.333838	-0.427430	0.001865
C	5.496077	-1.945786	0.061057
H	6.019719	-2.895156	0.098013
C	-3.057652	-1.636726	-0.131434

H	-2.511248	-2.571574	-0.226754
C	-7.262016	0.635363	0.054882
H	-8.298866	0.303017	0.002083
H	-7.057985	1.326183	-0.770085
H	-7.088249	1.136653	1.012986
H	1.577675	-1.502592	0.154198

Supplementary Table 101. Cartesian coordinates of optimized $MeOSQH^+$ in the excited (π,π*) state calculated at PBE0-D3/6-31G(d,p) level with the SMD model of DCM.

	X	Y	Z
O	1.565626	2.910094	-0.069039
N	3.475519	1.688033	-0.041709
H	3.990575	2.563214	-0.054994
O	-6.468518	-0.533935	0.022099
C	5.566447	0.468849	0.014725
H	6.122866	1.401298	-0.005569
C	2.095022	1.808001	-0.046802
N	2.066440	-0.627996	0.008408
C	1.354023	0.544904	-0.027789
C	4.097491	-1.937230	0.072892
H	3.519240	-2.856181	0.096361
C	-0.045516	0.583556	-0.044051
H	-0.447246	1.592045	-0.031197
C	4.169207	0.510630	-0.002680
C	-4.439646	-1.651928	-0.113435
H	-5.009662	-2.573527	-0.174916
C	6.212730	-0.753819	0.059681
H	7.297566	-0.785761	0.073132
C	-0.900420	-0.509467	-0.081858
H	-0.491958	-1.516421	-0.143398
C	-5.143649	-0.430928	-0.000895
C	3.424289	-0.705854	0.025489
C	-3.056965	0.774529	0.050188
H	-2.530628	1.720861	0.118220
C	-4.433597	0.786225	0.079750
H	-4.960155	1.729698	0.168094
C	-2.324117	-0.440560	-0.059100
C	5.477643	-1.956413	0.089272
H	6.001122	-2.906292	0.124792
C	-3.068404	-1.651334	-0.140310

H	-2.530215	-2.591524	-0.226015
C	-7.260652	0.645649	0.141261
H	-8.295933	0.305422	0.140485
H	-7.093009	1.316515	-0.707907
H	-7.045379	1.167678	1.079611
H	1.558917	-1.505414	0.053890

Supplementary Table 102. Cartesian coordinates of optimized DPASQH^{+} in the ground state calculated at PBE0-D3/6-31G(d,p) level with the SMD model of DCM.

	X	Y	Z
O	4.351808	-2.766463	0.845805
N	-3.849411	0.082685	-0.032243
N	6.229512	-1.537920	0.502861
H	6.780938	-2.345135	0.775931
C	0.355658	0.238148	-0.081281
C	1.769388	0.352487	-0.109124
H	2.132866	1.348643	-0.362844
C	2.678275	-0.646885	0.162330
H	2.346310	-1.640139	0.443227
C	6.724713	1.927845	-0.609693
H	6.106744	2.772580	-0.900684
N	4.738131	0.592451	-0.217929
C	-1.703944	-1.014037	0.250597
H	-2.202036	-1.939097	0.517366
C	-4.641251	1.263592	0.081799
C	8.280954	-0.280366	0.149771
H	8.873741	-1.141587	0.443960
C	-6.244216	-2.651182	0.712611
H	-7.008603	-2.885879	1.447625
C	-5.970436	-3.543456	-0.322821
H	-6.524026	-4.474599	-0.399936
C	-0.429445	1.378303	-0.383269
H	0.072226	2.306136	-0.646949
C	-1.801905	1.340007	-0.368563
H	-2.369830	2.227813	-0.621161
C	-0.334172	-0.959810	0.238172
H	0.221738	-1.855921	0.495506
C	-2.479351	0.136961	-0.051475
C	-5.536141	-1.458741	0.818129
H	-5.740347	-0.760908	1.624649

C	-4.420028	2.156674	1.132943
H	-3.633236	1.952379	1.853032
C	-6.232801	3.535125	0.328675
H	-6.854017	4.420748	0.424652
C	4.877394	-1.711971	0.525753
C	-4.551076	-1.155366	-0.123818
C	6.888798	-0.377973	0.146999
C	-5.212276	3.294263	1.247094
H	-5.039338	3.986925	2.065665
C	-6.457808	2.633103	-0.709998
H	-7.251623	2.814949	-1.428647
C	6.115253	0.729285	-0.231338
C	-5.663673	1.498482	-0.839391
H	-5.829686	0.792882	-1.647959
C	8.881097	0.912075	-0.224638
H	9.963845	0.986620	-0.222277
C	-4.282229	-2.039168	-1.171803
H	-3.526678	-1.783468	-1.908626
C	4.073875	-0.511601	0.131564
C	8.106534	2.015060	-0.604793
H	8.587425	2.942864	-0.896651
C	-4.987696	-3.234658	-1.261721
H	-4.776783	-3.919923	-2.077585
H	4.207359	1.408143	-0.509831

Supplementary Table 103. Cartesian coordinates of optimized DPASQH$^+$ in the excited (π,π*) state calculated at PBE0-D3/6-31G(d,p) level with the SMD model of DCM.

	X	Y	Z
O	4.278393	-0.919352	2.767208
N	-3.951771	0.028076	-0.100440
N	6.157955	-0.517603	1.563338
H	6.695811	-0.794666	2.375625
C	0.284577	0.055186	-0.273012
C	1.724155	0.085828	-0.390191
H	2.090045	0.359496	-1.378498
C	2.614831	-0.206150	0.612420
H	2.259663	-0.507685	1.593089
C	6.704574	0.645465	-1.879990
H	6.100499	0.937988	-2.734498
N	4.694907	0.213556	-0.590536

C	-1.777245	-0.244977	0.987651
H	-2.294218	-0.469998	1.915832
C	-4.611909	1.241590	0.060835
C	8.223838	-0.117017	0.342304
H	8.803201	-0.414243	1.212071
C	-6.481602	-2.615013	0.384501
H	-7.416782	-2.767241	0.913663
C	-5.913301	-3.657006	-0.350893
H	-6.417465	-4.616063	-0.413174
C	-0.494411	0.333375	-1.415444
H	0.005083	0.568106	-2.351740
C	-1.879851	0.323214	-1.368993
H	-2.470808	0.543130	-2.253028
C	-0.393684	-0.235972	0.930724
H	0.165086	-0.461745	1.833061
C	-2.513967	0.034407	-0.163139
C	-5.844123	-1.390218	0.470794
H	-6.257102	-0.600429	1.088654
C	-3.958032	2.316282	0.699372
H	-2.955813	2.185931	1.090051
C	-5.909856	3.695595	0.351362
H	-6.413520	4.650008	0.466555
C	4.793471	-0.565532	1.710349
C	-4.614515	-1.192325	-0.195570
C	6.831998	-0.135418	0.415830
C	-4.615727	3.522777	0.851670
H	-4.117746	4.339016	1.364877
C	-6.543895	2.646389	-0.317411
H	-7.531939	2.788286	-0.743263
C	6.068627	0.246571	-0.701571
C	-5.908235	1.427428	-0.469929
H	-6.378868	0.633594	-1.038942
C	8.849679	0.280102	-0.833689
H	9.933827	0.292449	-0.884148
C	-4.023158	-2.258820	-0.903708
H	-3.069674	-2.117556	-1.399064
C	4.016288	-0.169690	0.527532
C	8.090913	0.661374	-1.943068
H	8.582270	0.971456	-2.859837
C	-4.682160	-3.471497	-0.987106

H	-4.234540	-4.282474	-1.552462
H	4.164544	0.513558	-1.398811

Supplementary Table 104. Cartesian coordinates of optimized DPASQH$^+$ isomer in the ground state calculated at PBE0-D3/6-31G(d,p) level with the SMD model of DCM.

	X	Y	Z
O	-4.556615	-2.401900	-1.713941
N	3.929759	-0.105475	-0.642744
N	-6.312436	-1.289660	-0.793127
H	-6.939221	-1.973448	-1.203903
C	-0.312584	0.141340	-0.285421
C	-1.756851	0.239149	-0.110982
H	-2.104998	1.061128	0.512973
C	-2.679245	-0.592560	-0.631066
H	-2.402540	-1.441651	-1.248868
C	-6.424101	1.732465	1.274555
H	-5.708995	2.435945	1.690661
N	-4.575024	0.546787	0.306492
C	1.676239	-0.868950	-1.242471
H	2.142002	-1.611908	-1.884098
C	4.664335	1.171881	-0.434951
C	-8.212587	-0.124351	0.166618
H	-8.898619	-0.847904	-0.265298
C	6.145054	-2.950385	0.324388
H	7.011193	-3.468771	-0.074640
C	5.588516	-3.340626	1.540999
H	6.023043	-4.167582	2.094224
C	0.510171	1.032884	0.419478
H	0.056330	1.777450	1.067049
C	1.895315	0.984844	0.311614
H	2.515537	1.684440	0.861084
C	0.298221	-0.806304	-1.127007
H	-0.306428	-1.500787	-1.700635
C	2.455704	0.027944	-0.518810
C	5.591943	-1.892030	-0.388743
H	6.022410	-1.571246	-1.333921
C	4.633239	2.086453	-1.479828
H	4.103156	1.853412	-2.399838
C	5.949386	3.588520	-0.126783
H	6.458818	4.539471	-0.004528

C	-4.977432	-1.469784	-1.036851
C	4.479252	-1.247677	0.135690
C	-6.837339	-0.261926	-0.047071
C	5.286882	3.303018	-1.319615
H	5.277187	4.025868	-2.129346
C	5.961713	2.656435	0.906903
H	6.478848	2.875959	1.835706
C	-5.925922	0.666009	0.505382
C	5.316000	1.429864	0.761049
H	5.331523	0.698845	1.561813
C	-8.674231	0.936116	0.928853
H	-9.741765	1.044734	1.096456
C	3.904813	-1.616888	1.343000
H	3.031747	-1.102027	1.729016
C	-4.108622	-0.438067	-0.406016
C	-7.783192	1.867656	1.484924
H	-8.163745	2.692322	2.079365
C	4.474817	-2.675402	2.046868
H	4.038363	-2.979237	2.993082
H	4.096012	-0.349145	-1.627414

Supplementary References

[1] Y. Tu, Y. Yu, D. Xiao, J. Liu, Z. Zhao, Z. Liu, J. W. Y. Lam, B. Z. Tang, *Adv. Sci.* **2020**, *7*, 2001845.

[2] C. Reichardt, *Chem. Rev.* **1994**, *94*, 2319.
